# Patient-Specific Inverse Modeling of In Vivo Cardiovascular Mechanics with Medical Image-Derived Kinematics as Input Data: Concepts, Methods, and Applications

**DOI:** 10.3390/app12083954

**Published:** 2022-04-14

**Authors:** Johane H. Bracamonte, Sarah K. Saunders, John S. Wilson, Uyen T. Truong, Joao S. Soares

**Affiliations:** 1 Department of Mechanical and Nuclear Engineering, Virginia Commonwealth University, Richmond, VA 23284, USA; 2 Department of Biomedical Engineering and Pauley Heart Center, Virginia Commonwealth University, Richmond, VA 23219, USA; 3 Department of Pediatrics, School of Medicine, Children’s Hospital of Richmond at Virginia Commonwealth University, Richmond, VA 23219, USA

**Keywords:** inverse models, data assimilation, cardiovascular imaging, image-based kinematics, biomechanics, tissue mechanics, hemodynamics, patient-specific models

## Abstract

Inverse modeling approaches in cardiovascular medicine are a collection of methodologies that can provide non-invasive patient-specific estimations of tissue properties, mechanical loads, and other mechanics-based risk factors using medical imaging as inputs. Its incorporation into clinical practice has the potential to improve diagnosis and treatment planning with low associated risks and costs. These methods have become available for medical applications mainly due to the continuing development of image-based kinematic techniques, the maturity of the associated theories describing cardiovascular function, and recent progress in computer science, modeling, and simulation engineering. Inverse method applications are multidisciplinary, requiring tailored solutions to the available clinical data, pathology of interest, and available computational resources. Herein, we review biomechanical modeling and simulation principles, methods of solving inverse problems, and techniques for image-based kinematic analysis. In the final section, the major advances in inverse modeling of human cardiovascular mechanics since its early development in the early 2000s are reviewed with emphasis on method-specific descriptions, results, and conclusions. We draw selected studies on healthy and diseased hearts, aortas, and pulmonary arteries achieved through the incorporation of tissue mechanics, hemodynamics, and fluid–structure interaction methods paired with patient-specific data acquired with medical imaging in inverse modeling approaches.

## 1. Introduction

The primary role of numerical modeling in cardiovascular biomechanics has been to predict the performance of medical devices and to estimate physiological and mechanical cues acting on tissues, such as pressure and flow-driven stresses. Given the vast experimental evidence of mechanical factors producing effects on cellular differentiation, signaling, communication, and function [1–5], in silico experiments have explored the role of mechanical stimuli on normal and pathological tissue growth and remodeling [6]. From a clinical research standpoint, the development of patient-specific biomechanical models could provide more accurate and detailed data leading to a better understanding of the onset and progression of cardiovascular disease [7]. In addition, computational modeling has also been proposed as a supporting tool for medical practice on a patient-specific basis, which could provide non-invasive assessments of tissue properties, structure, and mechanical loads as physiologically meaningful risk stratification factors. Such patient-specific analyses have the potential to bring immense benefits to clinical practice by supporting diagnosis, treatment planning, and predictions of the outcome of surgical procedures with minimum associated costs and risk to the patients [8].

However, for biomechanical models to provide low-risk patient-specific solutions, personalized non-invasive clinical studies must be readily available to quantify regional cardiovascular function. Current medical imaging technology, namely echocardiography and magnetic resonance imaging (MRI), offers not only anatomical information but also high-resolution kinematics data of tissue motion and blood flow [9–12]. Kinematic-derived quantities, such as peak and average strain on the myocardium and aortic walls, have shown a good correlation with clinical risk markers [13]. Nevertheless, kinematic information alone cannot provide insights about mechanical forces, stresses, and tissue material properties, which are necessary for a full understanding of healthy and pathophysiological phenomena [14].

The inverse method, or data assimilation method, is an approach that allows solving classic mechanics problems “backwards”; that is, retrieving material properties and dynamic information (stress and forces) using measured kinematic information and loading boundary conditions as input [15]. Several research groups have coupled computational-mechanics tools with medical imaging technology to retrieve relevant biomechanical and hemodynamic markers from normal and pathological human tissues and organs [16], including diverse cardiovascular components [17]. Inverse modeling approaches have the potential to become a valuable tool for the non-invasive assessment of patient-specific cardiovascular health by providing quantitative physiological metrics that cannot be directly measured in vivo but may be derived entirely from clinical evaluations and the application of biomechanical principles.

The relevance of patient-specific modeling and its potential impact on the future of personalized and predictive health care has been acknowledged by several funding agencies. In 2003, the Interagency Modeling and Analysis Group was formed from the collaboration of nine institutes of the National Institutes of Health (NIH) and three directorates of the National Science Foundation (NSF). This group released its first funding opportunity in 2004 under the title “Interagency Opportunities in Multiscale Modeling in Biomedical, Biological, and Behavioral Systems Solicitation”, which has been regularly reissued since then, and led to the creation of the Multiscale Modeling Consortium which includes over 100 projects on multiscale modeling of biological systems. The European Union initiated the “Structuring the Europhysiome” Consortium in 2006, which led to the Virtual Physiological Human project, an ongoing initiative that aims to bring together policymakers, regulatory agencies, funding bodies, industry, and research organizations towards the development of integrated computer models of the mechanical, physical, and biochemical functions of the living human body [7]. These initiatives have motivated the integration of multidisciplinary teams of biologists, physicians, and engineers who are faced with the challenge of bringing together field-specific nomenclatures, techniques, and analytical approaches.

**Figure 1.**
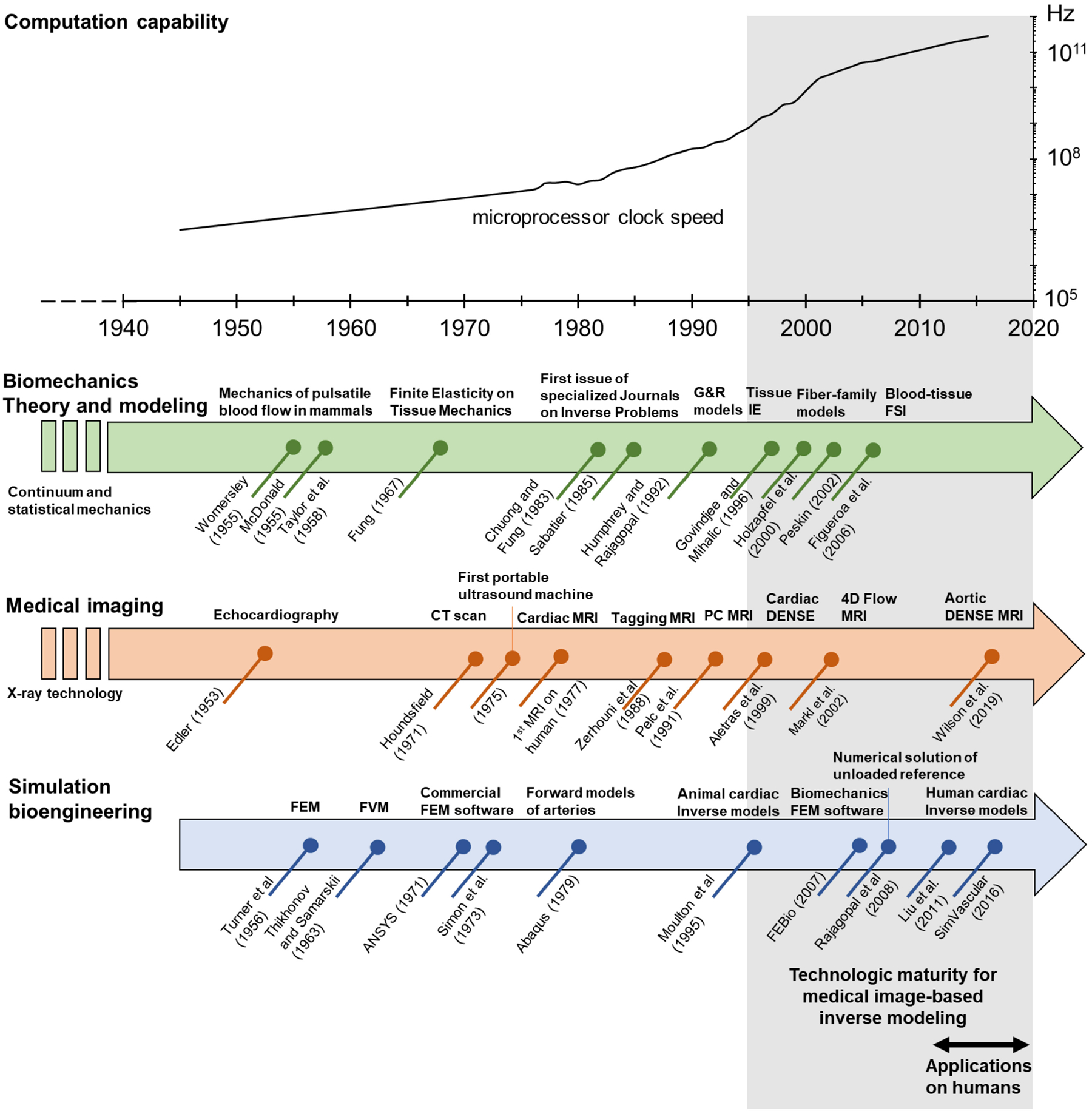
Timeline of microprocessor speed as a measure of computation capability, and relevant landmarks on the fields of biomechanics theory, medical imaging and simulation that make possible modern patient-specific image-based inverse modeling of the cardiovascular system. Acronyms: CT, computerized tomography; DENSE, displacement encoding with stimulated echoes; FEM, finite element method; FVM, finite volume method; IE, inverse elastostatics; FSI, fluid–structure interactions; PC, phase contrast; MRI, magnetic resonance imaging.

Inverse modeling of biomechanical systems requires the confluence of state-of-the-art techniques from several disciplines including clinical care, medical imaging, simulation engineering, data analysis, and computer science (Figure 1). Inverse methods have been developed on a highly specific basis and tailored to the available clinical data, tissue/pathology of interest, and available resources; thus, inverse modeling developments only share a general data processing pipeline, while differing on the clinical data source, imaging technique, and modeling approach. Therefore, the task of designing an inverse method pipeline requires a comprehensive understanding of the process at all stages, for which familiarity with fundamental concepts and terminology is a prerequisite. The latter can be challenging due to the multidisciplinary nature not only of the method itself but also of the clinically relevant phenomena to model.

Inverse modeling analyses can also be applied to in vitro experimental setups. The main advantage of this approach is that input data is not limited by the available clinical tests, mechanical loads can be precisely controlled, and kinematics can be measured with high-resolution instruments. Furthermore, the target of the inverse method can be defined not only in terms of kinematic information but also in terms of controlled mechanical loads and stress measurements [18]. Moreover, the outputs of inverse modeling can be validated with controlled experimental parameters. Inverse analyses of in vitro setups have been applied to explanted animal and human tissue, and to engineered tissue constructs. Notably, inverse modeling has been applied to resolve mechanics at a cellular level. The traction force microscopy (TFM) technique was introduced by Butler et al., to estimate the force that adherent cells exert on their surroundings by solving the traction field in a hydrogel of known properties, cultured with cells, and with embedded beads as visual markers [19]. By tracking the bead displacements through microscopy, and setting known boundary conditions, the traction field is resolved by an exact solution assuming a semi-infinite medium. Further development was introduced by Tambe et al., with the monolayer stress microscopy (MSM) technique, which allowed the inverse estimation of stress fields across monolayer cellular constructs under static and dynamic conditions by inducing controlled displacements of the boundary under a motorized microscope [20]. These, and other similar techniques, have been used to explore the response of cardiovascular cells (endothelial cells, cardiomyocytes, smooth muscle cells, etc.), cellular layers, and engineered tissue to mechanical stimuli in terms of cell proliferation, migration, expression, and synthesis of extracellular matrix components [21–23]. The detailed and accurate results that can be retrieved from inverse analyses of in vitro experiments can provide valuable insights into cardiovascular mechanobiology. These insights contribute to the understanding of how macroscopic biomechanical factors affect the healthy or pathological growth and remodeling of cardiovascular and engineered tissues. However, the replication of in vivo physiological conditions in vitro is cumbersome, and the results of in vitro experimentation can be challenging to extrapolate to patient-specific situations. As a result, the clinical application of inverse analyses of in vitro experiments remains limited.

This article aims first to serve as a referential document for concepts and methods from all involved disciplines on patient-specific in vivo inverse modeling; and secondly, to highlight the potential clinical application of patient-specific inverse modeling in the cardiovascular research field. Specifically, we review the fundamentals of cardiovascular tissue and blood biomechanics, modeling and simulation, and medical imaging, as they relate to the inverse modeling approach and its applications in cardiovascular medicine. In Section 2, we review the application of the principles of classical continuum mechanics to the study of blood and tissue motion, with special emphasis on the constitutive equations that have been proposed to describe the mechanical behavior of cardiovascular tissue and blood. Section 3 briefly summarizes the fundamentals and main features of the finite element method (FEM) and finite volume method (FVM), as the most popular formulations for the numerical solution of biomechanical models. Next, we review the general definition of inverse problems and the available alternatives to solve inverse mechanics problems in Section 4. In Section 5, we review the working principles and main features of ultrasound (US), magnetic resonance imaging (MRI), and computational tomography (CT) imaging, giving special attention to the available techniques for the resolution of tissue and blood kinematics. Finally, in Section 6, we present a comprehensive review of the applications of imaging-based inverse modeling approaches to patient-specific human cardiovascular mechanics, including the resolution of the unloaded configuration and the estimation of tissue properties and stresses. Reviewed applications include healthy and diseased heart valves, cardiac and arterial walls, and hemodynamics of large arteries. To highlight the potential application of the inverse-modeling approach in cardiovascular medicine, we focus herein mostly on developments made in human studies, with a few mentions of relevant and pioneering studies in animals.

## 2. Governing Principles of Biomechanics

Modern biomechanics consists of the formulation of governing equations describing balances of mass, linear and angular momentum, and energy to biological systems and physiological processes. The human body maintains a uniform and stable temperature through homeostatic thermoregulation. Thus, contributions due to temperature change in the internal energy of the material, heat fluxes, and heat supply are typically negligible to the energy balance, which is in turn reduced to the balance between deformational energy and stress power (thermodynamic work). In classical continuum mechanics of purely mechanical processes, the balance of angular momentum directly translates to the symmetry of the stress tensor, and therefore, the relevant governing equations for most cardiovascular mechanics applications consist only of the balances of linear momentum and mass. However, most biological systems are open, and continuously interact with their surroundings, and thus the conservation principles must be handled carefully, especially with respect to tissue growth and atrophy within relevant timescales.

Given that the resolution of most in vivo medical imaging is on the scale of millimeters, only phenomena occurring at the tissue level can be directly associated with these measurements. The assumption of material continuity is reasonable for the formulation of the governing principles at this scale, leaving any additional considerations dealing with the extracellular and intracellular micro-environments to be included ad hoc with additional modeling formulations and constitutive equations.

To apply these principles, it is necessary to relate the stress tensor to kinematic measures, which is in essence the description of the mechanical behavior of the material under study. This information is provided by a constitutive equation; these can be either phenomenological equations “arbitrarily” formulated to reproduce experimental observations, or analytical expressions inspired by theoretical interactions of the material constituents at the micro or molecular scale. The selection of adequate models to describe the phenomena of interest is key to the success of any engineering analysis. The selected model must be complex enough to describe the most salient observable features at the scale of interest, while ideally being simple enough to provide a rational interpretation of its parameters and results and render a computationally tractable numerical problem. After fitting these parametrized models to experimental data, the constitutive equation can provide an additional understanding of the underlying mechanisms associated with the mechanical response of the material. Models of increased complexity usually require a larger number of parameters to be fitted, and overparametrized models can lead to solution multiplicity which obscures its interpretation and validity. For the sake of generality, the constitutive equation must also be independent of the frame of reference, comply with the second principle of thermodynamics, and yield amenable mathematical treatment and systems of equations that are solvable [24].

After formulating the constitutive relation as a function of specific unknowns (e.g., displacement or velocity fields), the resulting system of governing equations is then particularized into specific problems by the definition of temporal and spatial domains of interest and the imposition of appropriate boundary and initial conditions. To obtain a unique solution, it is necessary to constrain the problem by assigning a first-order boundary condition on at least one of the boundaries (e.g., by prescribing a known displacement or velocity). On the rest of the boundaries, higher-order boundary conditions can be applied to impose distributed forces such as a known pressure. Once solved, the result of this forward formulation is the transient spatial distribution of displacement or velocity throughout the domain as a result of the specified loads and material properties. From this kinematic information, strain and strain-rate distributions can be numerically derived, and the stress distributions retrieved via the constitutive equation.

In the following subsections, we present a review of the main features of the mechanical behavior and composition of cardiovascular tissues and blood, as well as the constitutive equations that have been developed and applied to model such behaviors. Then, we review the algorithms implemented to model fluid–structure interactions and their importance in cardiovascular mechanics simulations. Finally, we briefly discuss the modeling of biological tissue growth and remodeling by the application of the constrained mixture theory.

### 2.1. Structural Mechanics of Cardiovascular Tissue

**Figure 2.**
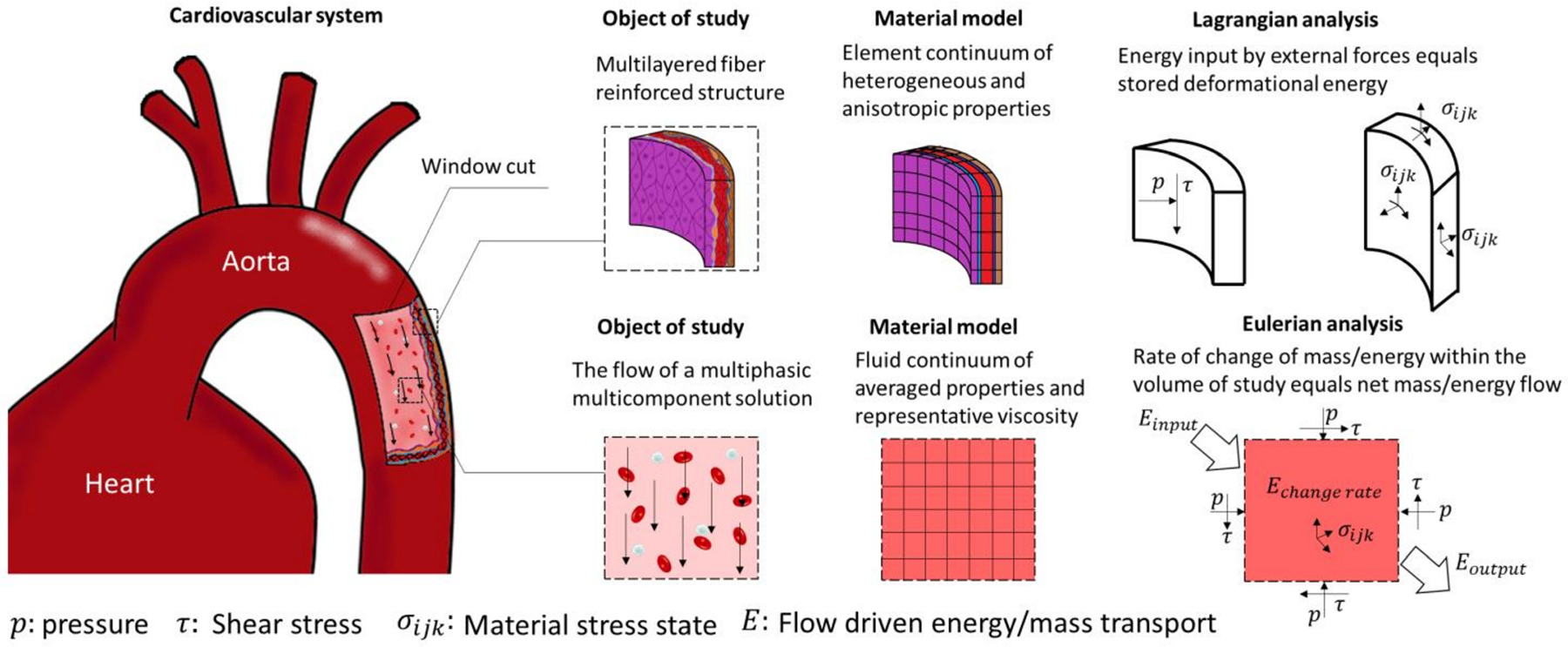
Representation of the modeling process of structural mechanics of cardiovascular tissue and fluid mechanics of the blood flow with a continuum mechanics approach. Structural mechanics of cardiovascular tissue are usually analyzed with a Lagrangian formulation that follows the deformation of a given portion of the tissue. Blood flow mechanics is usually analyzed with an Eulerian formulation, that is, analyzing the mass and energy balances on a fixed volume of interest through which the fluid flows.

The study of soft biological tissues under the framework of finite elasticity was initiated by Y.C. Fung and others in the late 1960s, setting the basis of modern biomechanics [25,26]. As in classical solid mechanics, the mechanical analyses of cardiovascular tissues are usually performed with a Lagrangian formulation of the governing principles (Figure 2). Applied forces are imposed as boundary conditions. For blood vessels, these are prescribed as transmural pressure differences that often assume a traction-free condition on the adventitial surface. More recently, however, growing attention to the role of perivascular and pericardial support and tethering has promoted the inclusion of restrictions to the displacement of the outer surface of the heart and vasculature [14,27,28]. In addition, more complex formulations of cardiovascular tissue mechanics which departs from classical elastic solids have been proposed to account for complex microstructural compositions, the inclusion of pre-stress/strains, chemically activated muscular tone, and viscous energy dissipation.

Cardiovascular tissues are comprised of multiple layers of cells and extracellular matrix (ECM) components. The ECM is a network of macromolecules that is continuously synthesized and degraded by active cells and functionally provides them with structural and biochemical support. Typically, collagen, elastin, and fibrillin are regarded as the main structural constituents responsible for the macroscopic mechanical behavior of cardiovascular tissues [29]. Healthy cardiovascular tissue retains residual stress even when unloaded (i.e., the tissue is pre-strained relative to a reference state of zero transmural pressure). Circumferential and axial pre-stress/strain in vascular conduits have been widely established by measuring how much these tissues recoil to an open configuration when excised and cut transversely and longitudinally to relieve the residual stress [30,31]. It has been hypothesized that pre-strain plays a relevant role in balancing higher stresses on the luminal surface of blood vessels and promoting a homogenized transmural stress distribution and homeostatic equilibrium of the vascular tissue [32]. Notably, residual strain is heterogeneous and has been shown to vary with patient age and health, likely as a consequence of heterogeneous growth and remodeling and/or damage.

Additionally, cardiovascular tissue is muscular in nature and actively contracts/distends. Thus, its mechanical behavior is affected by the activation of actin-myosin sliding filaments, which depends on ion-based chemical signaling and determines the muscular tone. In the myocardium, striated muscle activation is responsible for cardiac contraction. In large arteries, contraction of smooth muscle cells regulates downstream vascular resistance, blood flow, and propagation of the pressure-pulse wave along the vascular tree.

Of note, cardiovascular tissue also exhibits viscoelastic behavior, which has been established with stress relaxation, creep, and strain-rate experiments. It has been argued that viscous energy dissipation of healthy tissue, functioning at a regular physiological rate (~1 Hz), is negligible compared to stored strain energy [33]. Nevertheless, viscoelasticity may play a critical role under pathological conditions where the deformation rate is increased, such as in atrial fibrillation, or when dealing with highly dissipative structures such as lipid pools in atherosclerotic plaques [34,35]. Despite the relevance of viscoelastic properties to pathological conditions, standardized testing protocols have yet to be developed for the exploration of its relation to disease onset and progression [36]. Notably, if viscous dissipation and inertial effects are neglected, all temporal terms in the governing equations are canceled, rendering the problem a quasi-static process (which is the most common approach applied to vascular wall mechanics).

#### 2.1.1. Constitutive Equations

##### Passive Properties

Linearized elasticity falls short in describing the complex behavior of biological tissue, not only because the mechanical response is highly non-linear but also the material undergoes finite motions and deformations. In the case of non-linear behavior, it is usually convenient to employ the formulation of hyperelasticity and express the constitutive equations as the relation of a scalar stored energy density function to the deformation gradient tensor or the strain tensor (while other definitions of stretch or strain tensors are also possible and common). The scalar energy density function represents the amount of deformational energy stored per unit volume and is defined in such a way that the stress tensors can be obtained from their derivatives with respect to the strain or stretch tensor.

**Figure 3.**
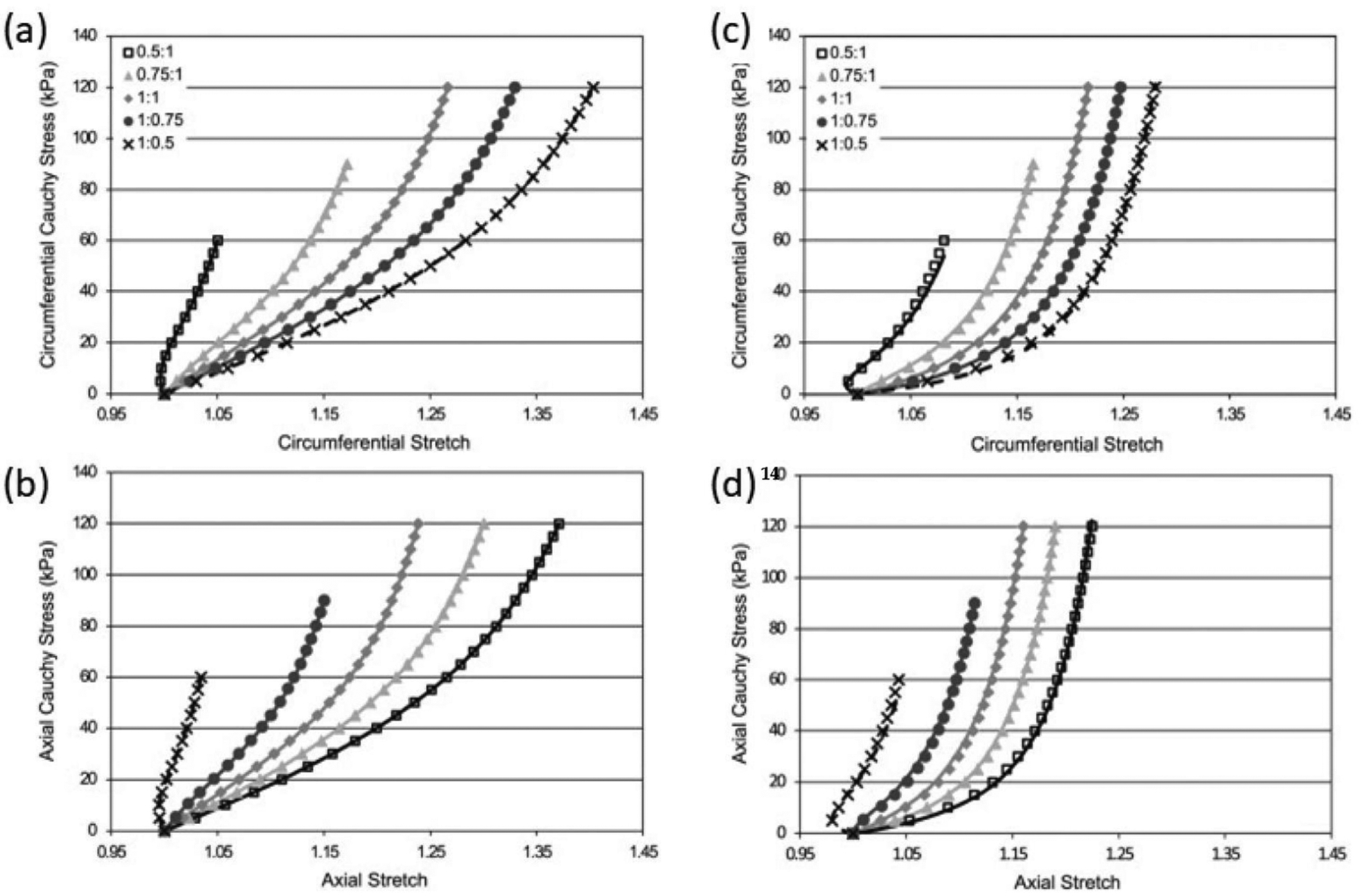
Representative biaxial stress-stretch behavior of healthy cardiovascular tissue. The stress and slope increase with the stretch/strain in any direction. This suggests that the cardiovascular tissue stiffens with stretch/strain which is hypothesized to be a consequence of the progressive engagement of ECM components to resist further deformation. This behavior is modeled by exponential functions of the deformation tensor components and/or invariants. Mark symbols in the figures show experimental biaxial test data of the human thoracic aorta for (**a**,**b**) young patients (20 to 35 years of age), and for (**c**,**d**) older patients (57 to 71 years of age). Solid lines represent the best-fit approximation with a four-fiber family constitutive equation. Reprinted/adapted with permission from Ref. [37], 2014, Elsevier.

The passive behavior of cardiovascular tissue is characterized by an increasing resistance to deformation with strain. This behavior is represented by an increasing slope in the stress versus strain/stretch and strain energy versus strain/stretch curves, with a close to zero slope at zero strain and rapidly increasing at physiological ranges. This behavior has been attributed to the structural characteristics of the ECM components. It has been suggested that the increasing resistance to deformation with strain is owed to the progressive engagement of wavy bundles of elastin and collagen fibers to support the mechanical loads (Figure 3).

Hyperelastic isotropic material models, such as the Neo–Hookean and the Mooney–Rivlin constitutive equations, can accurately describe the behavior of amorphous bodies such as lipid pools in atherosclerotic formations, and fit some portions of the pressure–volume relation of blood vessels. The symmetry of these material models allows its representation as to the linear combination of the deformation gradient invariants weighted by the material properties. This formulation allows the determination of unique material properties for a given mechanical behavior. However, these models fail to reproduce the characteristic highly nonlinear and anisotropic behavior of cardiovascular tissue (Figures 2 and 3). This was originally addressed by the use of phenomenological equations, e.g., the Fung orthotropic exponential model being one of the most commonly used. Its success relies on its relative simplicity, widespread numerical implementation, and accuracy in the prediction of stress-strain curves [24,26]. Guccione et al. proposed modifications to Fung’s orthotropic model based on myofiber structure and orientation to tailor myocardial tissue behavior [38]. These phenomenological equations usually consist of two terms contributing to the strain energy function. First, the contribution of volume changes is written as a function of the determinant (third invariant) of the deformation gradient tensor. The second term is the deviatoric contribution to the strain energy function, defined to be proportional to an exponential function of the components of the strain tensor. The proportional constant sets the scale of the material stiffness, and the function of the strain tensor components defines the material anisotropy. Part of the success of Fung-like equations in describing the stiffening of cardiovascular tissue with strain relies on the exponential functional form to quantify the effect of strain increments on deformational energy. However, the fitted constants of phenomenological equations lack physical interpretation which is desirable for studies aiming to relate material properties with pathological conditions [39,40].

In the past decades, many microstructure-inspired constitutive models have been proposed to specifically suit cardiovascular tissue behavior [39,41]. Fiber-family models have been particularly successful in reproducing the anisotropic behavior of the vascular wall while keeping physiological meaning to some of the fitting constants, the Holzapfel–Ogden model and its many variants being the most popular for cardiovascular tissue. These models assume that families of 1D fibers, each with specific mechanical behavior, orientation distribution, and volume fraction, are embedded within an isotropic continuum matrix. The isotropic component of the strain energy function is usually defined as a function proportional to the first invariant of the deformation gradient tensor. The contribution of fiber families is a weighted sum of exponential functions, the weighting factors being the fiber family material parameters. The exponential functions are defined depending on deformation tensor invariants and the relative orientation of fibers to strain, such that the contribution of a fiber family is maximized if the strain deformation occurs in the direction of the fibers [37]. Again, exponential functions are employed to mimic the stiffening effect of strain on cardiovascular tissue (Figure 3), this time being directly attributed to the ECM fiber components. Many improvements to these models have been proposed to account for different coupling effects such as inextensibility of fibers or cross-linking of the fiber ensembles [42–45].

##### Active Properties

Adequate modeling of active contraction is key for an accurate description of cardiovascular function in general, being particularly critical for modeling the heart. Conceptually, there are two possible approaches, the active stress models are the most common and they assume the stress tensor can be decomposed as the sum of a passive and active component, while active strain models assume a product decomposition of the deformation gradient.

Most active contraction models used in the inverse analysis of ventricular mechanics through continuum mechanics are simplifications of more complex bio-chemo-mechanical models such as the work of Hunter et al. [46]. The latter proposes a four-state variable model which includes the passive elasticity of myocardial tissue, the binding of calcium ions (Ca^2+^) to troponin C and its release, tropomyosin movement kinetics, the myofiber length, and the kinetics of cross-bridge tension build-up under perturbation of myofilament length. In practice, the detailed information required for the evaluation of this model is out of reach, so simplified models assume the active stress acts mostly lengthways the direction of myofibers, with a magnitude that is proportional to the fiber length and the activation status. The activation status is often expressed as a time-dependent spatially heterogeneous function ranging from 0 to 1 [47]. Active strain function will impose the relative shortening lengthways of myofiber directions as a function of location and time along the cardiac cycle.

Typically, the activation state is assumed to be instantaneously homogeneous within the region of study, however, it is known that cellular activation propagates as an electrical wave and the excitation-contraction coupling poses a complex electromechanical problem [48]. This propagation has been modeled macroscopically as a reaction-diffusion problem of the electric potential through the intracellular and extracellular domains, thus known as the bidomain model. The monodomain model is a simplification that assumes the same propagation anisotropy for both domains. Some interesting research has been developed to apply inverse modeling to fit the parameters of mono and bidomain equations using patient-specific electrocardiography [49,50]. These, however, fall out of the scope of this review, and the interested reader is encouraged to study the abundant literature on the inverse problem of electrocardiography [51,52].

The application of active contraction models requires the specification of the local myofiber orientations. Patient-specific myofibers orientation can be resolved via diffusion tensor MR imaging (DT MRI) [53]. Being a relatively novel technique, these scans are rarely available from medical records of cardiovascular-disease patients, although their relevance in the biomedical field is a growing topic of discussion [54]. Therefore, myofiber orientation is either assumed a priori with simplified models or obtained through diffeomorphic transformations with the employment of precomputed cardiac atlases [55]. Bayer et al. proposed a Laplace–Dirichlet rule-based algorithm for assigning myofiber orientation to computational heart models that showed good agreement with DT MRI measurements. This algorithm consists of the resolution of the Laplace equation on the simulation domain with appropriate Dirichlet boundary conditions constrained by the following rules: the longitudinal fiber direction is parallel to the endocardial and epicardial surfaces, the longitudinal fibers rotate clockwise throughout the ventricular wall from a positive helical angle at the endocardium to a negative helical angle at the epicardium (both imposed by the user), fibers in the papillary muscles and trabeculae are assumed parallel to the long axis of these structures, the transverse fiber direction is perpendicular to longitudinal fibers, and fiber orientation in the septum is continuous with the ventricular walls [55]. Similarly, Potse et al. proposed a rule-based algorithm to define myofiber orientations assuming that longitudinal fibers are orthogonal to the local vector pointing to the shortest path between endocardium and pericardium, with a clockwise varying helical angle [56]. Rijcken et al. derived an equation for longitudinal and transverse myofiber orientation by solving an optimization problem, which maximized the ejection while maintaining fiber strain as homogeneous as possible on idealized geometries [57].

### 2.2. Fluid Mechanics of Blood Flow

For the study of fluids, it is more practical to implement an Eulerian formulation of the governing equations. This formulation is obtained by applying the Reynolds transport theorem to the equations for mass and momentum balance. Thus, this formulation solves the relation between flow driving forces, flow velocity, and deformation rates (Figure 2). A simplifying assumption applicable to biological systems is the incompressibility of the fluids, as most of them are either liquids or gases moving at subsonic velocities. Additionally, it is convenient to decompose the stress tensor into a spherical tensor representing the hydrostatic pressure and a deviatoric stress tensor. With this decomposition, constitutive equations can be designed to specifically relate the deviatoric stress components to the viscous dissipation of momentum.

Blood flow is generally assumed to be laminar throughout the circulatory system. The main arguments for this assumption are the pulsatile nature of the flow, the reduced dimensions of the vessels, and relatively low velocities, each contributing to the viscous effects overcoming the inertial forces and preventing turbulent random motion. However, it has been argued that transition to turbulent flows could be achieved locally in stenotic arteries. The use of a laminar model to study those cases could lead to an underestimation of wall shear stress, and stress oscillation [58,59]. Unlike most conventional engineering flows, blood flow is pulsatile and contained by compliant conduits of complex geometry. Since the 1950s, Womersley [60], McDonald [61], Taylor [62], Pedley [63], and others, developed analytical and experimental studies of pulsatile flow in mammals, identifying the most relevant parameters and features of this type of flow, thus setting the bases for modern hemodynamics.

Besides the intricacies of pulsatile flow in distensible conduits, the blood itself is a complex fluid. Blood consists of a suspension of cells in an aqueous solution of proteins and minerals called plasma. Plasma occupies approximately 55% of the blood volume, the rest being mainly occupied by red blood cells, white blood cells, and platelets. The rheological behavior of blood depends on how its constituents interact with each other and with the vessel walls, in consequence, this behavior is non-linear and highly dependent on the volumetric composition of blood, the flow conditions, and vessel dimensions. Modeling the complex interactions of blood constituents is a challenging statistical mechanics problem [64]. Some researchers have shown that cell aggregation and disaggregation are relevant to accurately describing blood rheology, especially in capillary flows where the cell size is comparable to the vessel diameter. Multiscale approaches have been successful in coupling the behavior of single cells as elastic entities with the transport equations of fluid flow, which are relevant for the study of clotting, aggregation, and platelet activation [65,66]. These approaches are computationally expensive, making them impractical for the study of large vessels.

#### Constitutive Equations

In the study of large and medium vessels (the ones that can be feasibly resolved with standard medical imaging), blood is often assumed to be a single-phase continuum. This approximation is reasonable given the relatively small size of cell aggregates compared to the vessel dimensions (and thickness of the boundary layer), and the relative relevance of inertia on flow motion [67]. For these cases, phenomenological constitutive equations describing the macroscopic behavior of flow are often applied. The linear Newtonian fluid is the simplest and most commonly employed model, providing reasonable results in vessels with diameters down to 200 μm [67]. Constitutive equations, such as the Casson, Herschel–Bulkley, and Carreu–Yasuda, incorporate the shear-thinning effect on apparent viscosity by introducing a yield shear stress term [68]. To account for the effect of the volumetric share of cell suspension, recent works have included the hematocrit as an independent variable for the estimation of the effective viscosity [69].

### 2.3. Fluid-Structure Interactions (FSI)

Mechanics of the vascular wall and hemodynamics have been mostly studied as isolated problems; however, the function of the cardiovascular system is the result of complex interactions between blood, the actively contractile cardiac tissue, and the compliant vascular walls. The interaction of fluids and solids can conceptually be achieved by coupling the boundary conditions on the interface between the solid and the fluid, such that the field of displacements, velocities, and stresses are continuous and derivable at all points in a monolithic fully coupled approach. This, however, poses many implementation difficulties for complex 3D domains that can only be solved numerically. In addition, the typically large deformations of the cardiovascular walls cannot be handled by linearized methods used in conventional engineering applications.

The immersed boundary method, introduced by Peskin, was originally developed for the study of flow around heart valves and was rapidly adopted for many other applications [70]. In this approach, the Eulerian variables of fluid dynamics describing the surrounding flow are defined on a fixed computational grid, while the Lagrangian variables, accounting for the deformation of the tissue structures, are defined in a curvilinear computational grid that can be displaced with no conforming constraints in respect to the Eulerian grid. The moving solid boundary interacts with the fixed fluid domain by means of elastic body forces which are modulated by Dirac delta-like functions [71,72]. The fictitious domain method is a generalization of the immersed boundary method, which solves the coupling of the Lagrangian and Eulerian domains by the use of Lagrange multipliers instead of the concept of body forces [73]. This method is computationally less demanding as it does not require fitting the interface boundary at the cost of impaired accuracy near the interface.

Similarly, Figueroa et al. proposed the coupled momentum method, consisting of changing the non-slip condition on the fluid boundary to a traction condition, which is strongly coupled to the degrees of freedom of modified thin-membrane elements. This allows the formulation of the solid equations on the same Eulerian frame as in the fluid equations. In consequence, the fluid–solid interface mesh remains fixed, while the boundary nodes will have nonzero velocities [74]. Another well-established method for FSI simulation is the arbitrary Lagrangian–Eulerian (ALE) algorithm, which allows the arbitrary convective motion of the computational nodes of the discretization grid with respect to a fixed reference frame. Typically, the nodes on the fluid-solid interface are treated with a Lagrangian formulation. To deal with large or heterogeneous deformations of the interface, several implementations include the re-discretization of the computational domain to avoid the influence of ill-shaped deformed elements. The drawbacks of this method are the computational expense of re-meshing the domain, and the induced inaccuracies by transferring solutions from the degenerated mesh to the new one [75,76].

### 2.4. Growth and Remodeling Models by the Constrained Mixture Theory

One of the most relevant characteristics of living tissue is its capability to adapt in response to chemical and mechanical stimuli. This adaptation comes with microstructural reconfigurations, which alter the mass composition and the resulting contributions and properties of the tissue constituents. The understanding of the effect of mechanical stimulation on normal and pathological growth and remodeling of soft tissues is an active field of study that bridges biomechanics and mechanobiology.

In 1994, Rodriguez et al. proposed a general continuum formulation for the finite volumetric growth modulated by mechanical stress [77]. The theory of adaptation of living tissues was further developed by Humphrey and Rajagopal who proposed the constrained mixture theory, a mathematical framework to predict not only the growth but also the remodeling of biological tissues under transient mechanical and chemical stimulation [78,79]. The constrained mixture theory is based on the continuum theory of mixtures; that is, each component complies with a modified version of the governing principles of motion in the Eulerian formulation. The modification involves the addition of mass source/sink terms that account for the rate of synthesis or degradation of the constituent in its respective mass balance equation and the component-to-component interaction forces in the momentum balance equation. These source/sink terms respond to a series of constraints of physical and chemical nature and are dependent on the local distribution of strains, stress, and current composition. Constitutive equations must be defined for each constituent, and the overall properties of the construct can be calculated as a combination of its constituents, where simplified linearized forms weighted by their volume fraction are typically chosen [32,80].

### 2.5. Summary

In Table 1 we summarize the highlights of the governing principles of cardiovascular biomechanics through a continuum mechanics approach.

## 3. Numerical Methods

The above-described system of governing and constitutive equations can only be solved analytically for a reduced group of oversimplified cases. Thus, mechanical analyses of complex biological systems require the application of numerical methods to obtain approximate solutions. It has been claimed that the development of numerical methods was key to the foundations of modern biomechanics [81,82]. Many of the early simulation analyses of the cardiovascular system and components were developed with in-house codes, but the popularization of commercial software boosted the production of computational research in biomechanics [82]. More recently, open-source specialized software for numerical biomechanics, such as SimVascular and FEBio [83,84], have risen from the collaborative effort of academic groups aiming to incorporate relevant bio-chemo-mechanical models of biological systems into simulation pipelines. Many different options exist for the numerical solution of time-dependent 3D problems. Mesh-based methods are the most popular approaches, particularly the finite volume and finite element methods which are reviewed in the following pages.

### 3.1. Finite Volume Method

**Figure 4.**
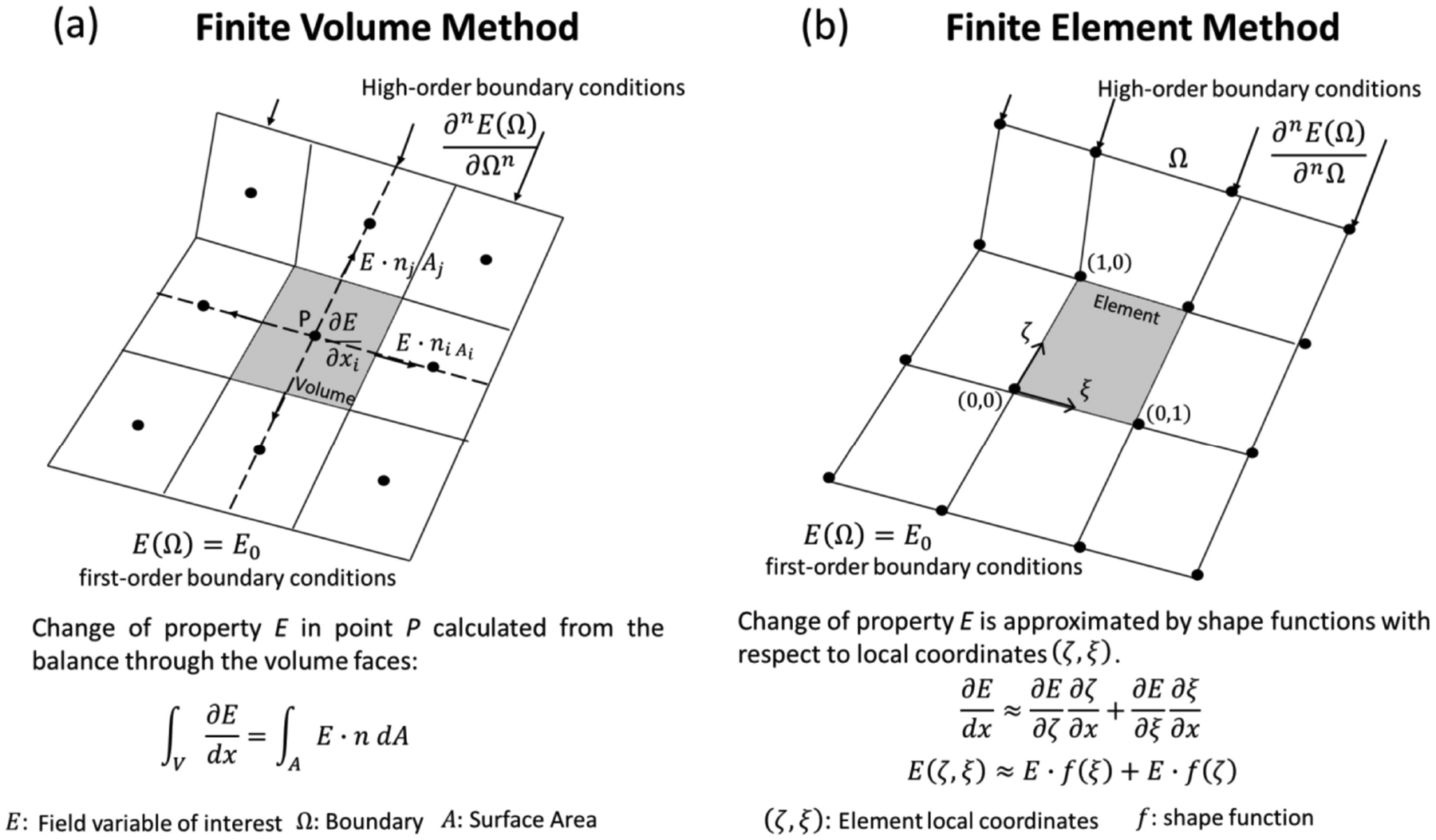
Diagram of finite volume (FVM) and finite element methods (FEM) approximation principles. (**a**) In FVM, the domain is discretized in finite volumes, and balance equations are solved at the center of each volume. (**b**) In FEM, the domain is discretized in finite elements, and the variables distribution is assumed to follow a prescribed shape function within each finite element.

The finite volume method (FVM) is conceptually straightforward. The domain of study is discretized in a series of non-overlapping finite volumes, and the governing equations, usually expressed in Eulerian formulation, are converted into algebraic expressions by integrating them over each discrete volume. The balance equations are applied on a node located in the center of the finite volume, while the flux terms are calculated at its faces (Figure 4a). This allows first and second-order approximations of derivatives. The surface flow for a given shared face is set identical and in opposite direction for the adjacent discrete volumes, and equal to a boundary condition at the edge of the domain. By doing so, the balance equations are held at the whole domain and within each finite volume, which is one of the most attractive features of the FVM. Additionally, since the calculation of properties happens in the center of each volume, it is relatively easy to implement boundary conditions of a higher order [85]. Numerical implementation of this method is also straightforward in the case of structured meshes, becoming more complex for unstructured meshes due to the bookkeeping necessary for the calculations of interface flux balances.

The use of FVM for the solution of convection-diffusion problems was first introduced in the early 1960s by Tikhonov and Samarskii [86]. Since then, FVM has been particularly successful in its application to computational fluid dynamics, as many of the current commercial computational fluid dynamics (CFD) software suites are based on this method. Biomechanical applications of this method mostly focus on hemodynamic and tracheobronchial airway simulations. However, this method can be applied to other boundary value problems such as electromagnetics and structural mechanics [87,88].

### 3.2. Finite Element Method (FEM)

The finite element method (FEM) consists of the discretization of the domain of study on simple geometrical elements (or finite elements), where the unknown fields are discretized as linear combinations of shape functions of any order, linear and quadratic being the most common. The shape functions are typically defined at each element depending on local and normalized coordinates (Figure 4b). The local governing equations for each element are then assembled and organized in a matricial system of algebraic equations. Finally, the solution is approximated by minimizing the weighted error associated with each element. Several weighting rules have been proposed, the Galerkin method and its variations being the most widely used [89]. By converging to the solution through the minimization of an error function and not through the exact solution of balance equations, FEM is said to be formulated in a “weak” form. However, the weak form is equivalent to the exact solution in the limit of refining the domain discretization. In fact, it has been widely shown that mesh-independent FEM solutions do not show any practical difference from the output of more conservative numerical methods such as FVM [90,91].

FEM was developed in the early 1950s to perform structural analysis for the aerospace industry and was soon applied to study the biomechanics of musculoskeletal and cardiovascular tissue [81,82]. As early as 1968, FEM was used to study the non-linear viscoelastic behavior of arteriole tissue [92]. This technique has been traditionally used for the solution of solid mechanics problems; however, it has also been used to solve the governing equations of other physical phenomena, including fluid mechanics [81]. Regarding the convenience of relying on a single solver engine, many multiphysics simulation software suites have introduced FEM formulations for fluid mechanics, [93] which also facilitates the implementation of FSI simulations [94].

### 3.3. Summary

In Table 2 we summarize the highlights of the principles and formulations of the numerical methods typically applied on cardiovascular biomechanics research.

## 4. Inverse Problems

**Figure 5.**
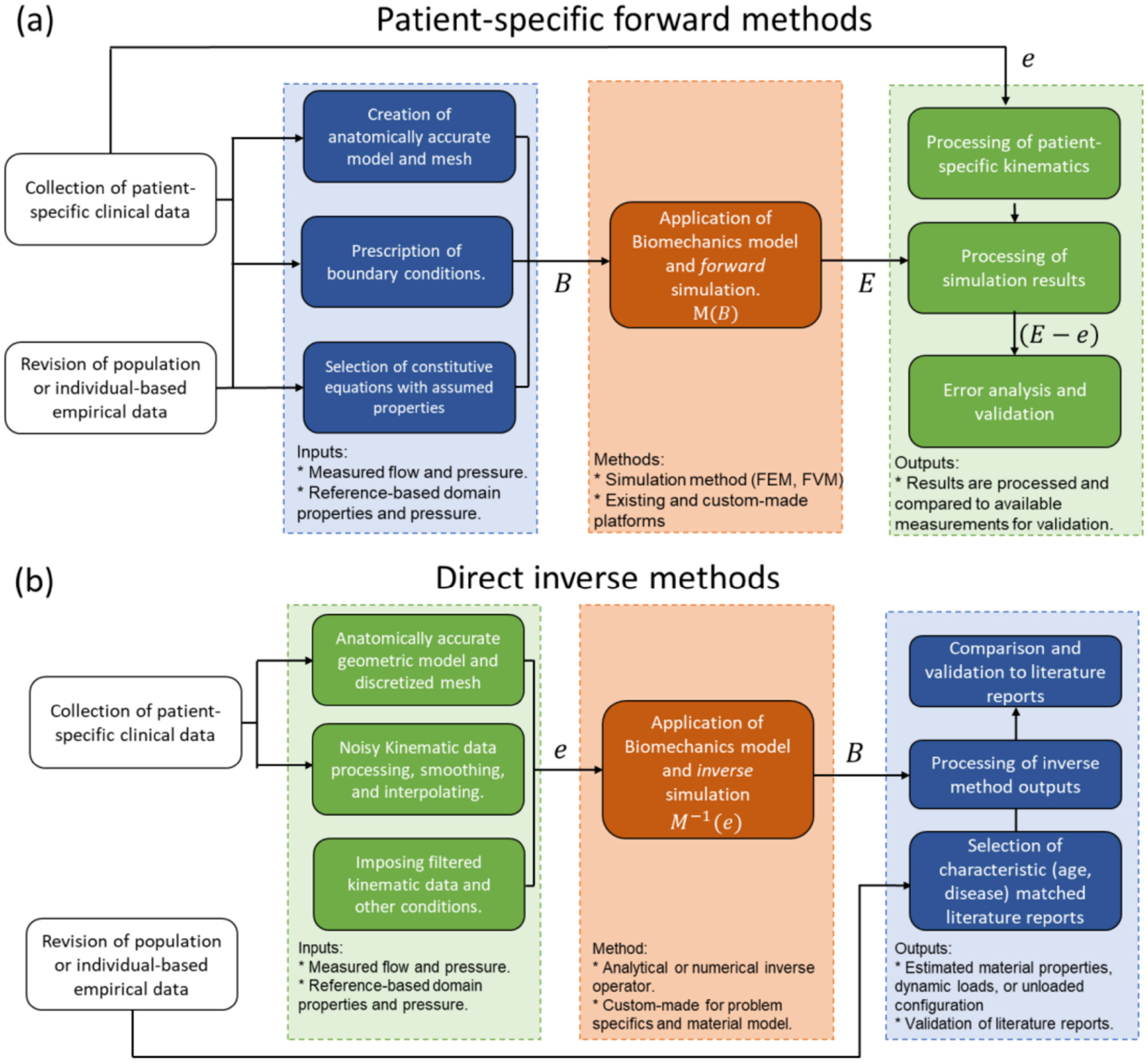
Data processing pipeline for patient-specific (**a**) forward problems and (**b**) direct inverse problems. Symbols, *B*: forward problem inputs, *E*: forward problem outputs, *e*: experimental data.

Modeling physical phenomena can be thought of as a mapping operation, where a set of inputs (*B*) is transformed into a set of outputs (*E*) by applying the model operator (*M*) such that *M*(*B*) = *E*. In the realm of physics, there must be a cause–effect relation between the inputs and outputs, and forward modeling consists of designing and applying a mapping function capable of producing outputs that closely follow experimental measurements (*e*), meaning that the difference *E* – *e* should be close to zero (Figure 5a) [95].

Conceptually, solving an inverse problem consists of using the measured effects to estimate the causes. That is, solving a problem of the type *B* = *M*^−1^(*e*), which could be straightforward if *M* was a bijective function, with *M* and *M*^−1^ being continuous and differentiable, and *e* was a continuous and smooth distribution (Figure 5b). The main difficulties with inverse problems are the possible nonlinearity of the inverse mapping function, the multiplicity of solutions, and the sparsity and noise of the measured effect data [15,95].

The development of advanced measuring techniques along with advances in computer science brought attention to the practical applications of inverse problems. A growing body of research has been built to address the afore-mentioned difficulties and to apply the inverse modeling methodologies to problems from many different engineering applications. To attend to the necessity of opening wide discussion of concepts, methodologies, and methods related to inverse formulations, specialized journals started circulating by the late 80s, e.g., the Inverse Problems and Inverse Problems in Engineering Journals (today Inverse Problems in Science and Engineering) among many others [95]. In this section, several solution methodologies for inverse problems in mechanics are reviewed, highlighting their respective advantages and drawbacks when incorporated into cardiovascular biomechanics analyses.

### 4.1. Direct Inverse Methods

The direct solution of inverse problems by the deduction of the inverse mapping function *M*^−1^) is only possible for oversimplified cases; however, specialized mathematics has been developed for the direct solution of some specific problems. In the case of finite elasticity, one relevant inverse problem is the retrieval of the mechanical properties of the domain of interest from the applied loads and measured displacement field. Several methods have been proposed to solve this problem directly, e.g., the reciprocity gap method which has been used to retrieve the distribution of elastic properties and to resolve the location of cracks in solid bodies from image-derived displacement fields. This method linearizes the inverse problem by assuming that the same elasticity tensor can resolve both the measured displacement field and a slightly perturbed version of it [15]. Another alternative for inverse elasticity is the application of the virtual work principle. This requires a complete description of the deformation field as a starting point, then the virtual work identity is defined by arbitrarily selecting a virtual field function. These functions can be tailored to specific constitutive equations to convert the virtual work identity into a set of algebraic equations from which the components of the elasticity tensor can be resolved [96,97].

Another relevant problem on inverse elasticity is the resolution of the unloaded geometric reference configuration with the applied loads, material properties, and deformed configuration as inputs. This problem has many applications in manufacturing engineering and is key to the study of patient-specific biomechanics. Govindjee and Mihalic proposed a finite element implementation for the direct solution to this problem [98,99]. The proposed method exploits the duality of the equations of finite hyperelasticity when the role of the reference and deformed coordinates are interchanged. In the absence of body forces and assuming material homogeneity, the FEM implementation of the inverse problem can be formulated similarly to the conventional FEM problem, requiring slight changes in the definition of elements and shape functions. The authors highlight that the application of the method to buckling problems can lead to multiple solutions for a given input and the resulting method was highly sensitive to input variations.

Direct solutions to inverse problems are computationally efficient; however, solution methods are not generalizable and need to be tailored for each specific inverse problem and each constitutive equation, making its implementation on existing numerical solvers a non-trivial process [100]. Another limitation of direct solutions is the requirement of a smooth continuous function of the measured effects (*e*), which cannot be satisfied by discrete empirical measurements affected by random error. The experimental variability and noise of the input data could be incompatible with the assumed model, which could dampen the convergence to a valid solution or any solution at all [95]. An option to deal with this issue is through preprocessing of the input data with smoothening and interpolation operations.

### 4.2. Iterative Inverse Methods

**Figure 6.**
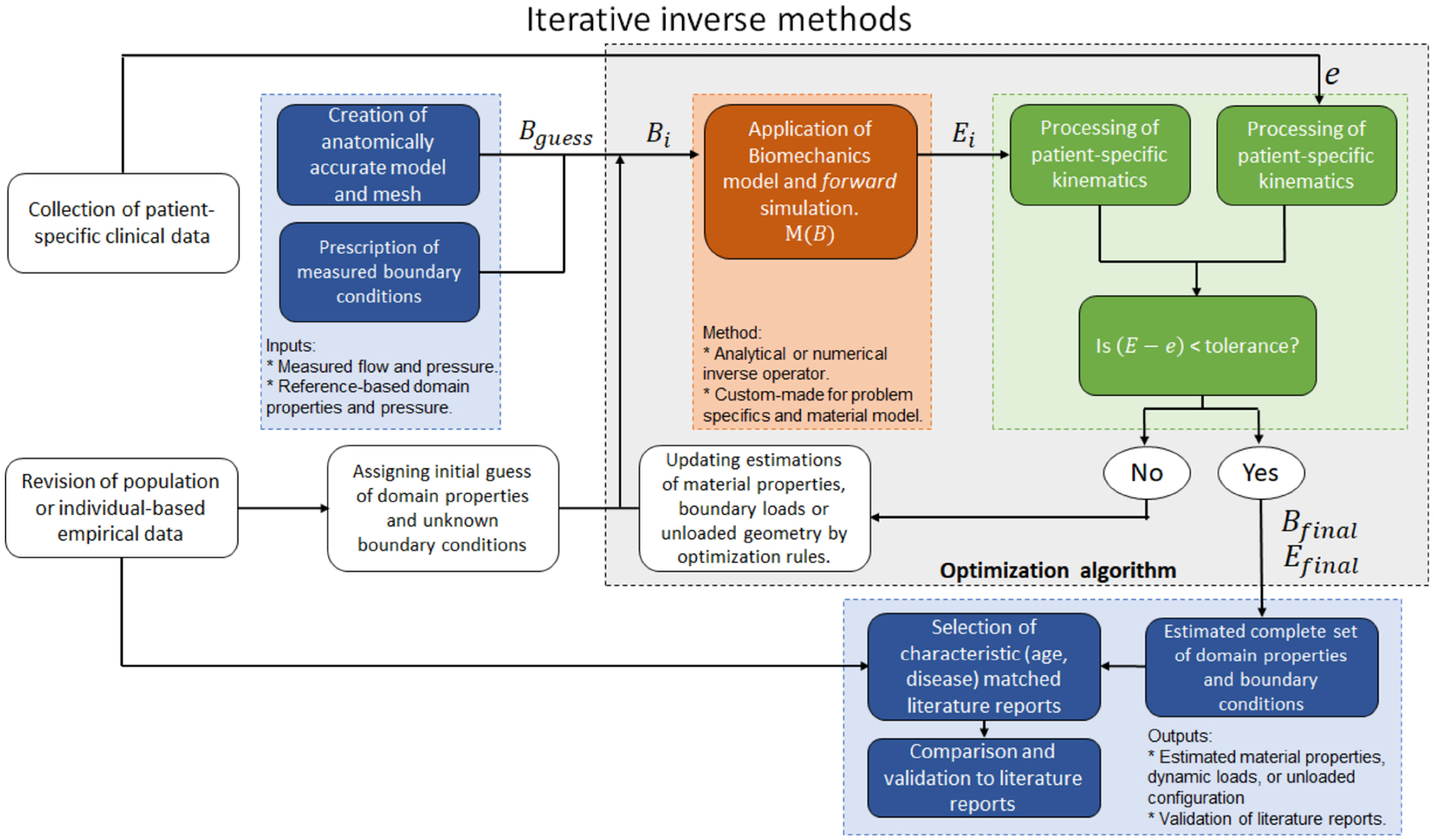
Data processing pipeline for patient-specific iterative inverse methods. Symbols, *B*: forward problem inputs, *E*: forward problem outputs, *e*: experimental data.

An alternative method for the solution of inverse problems is the iterative approach. This consists of optimization algorithms that iteratively solve the forward problem while varying the input parameters (*B*) until an error function defined as the difference between target experimental data (*e*) and the forward problem output (*E*) is minimized [101]. The advantages of this method are its easy generalization to any kind of inverse problem, its capability to operate on top of any existing solver for the forward problem, the existence of methods to reduce solution multiplicity, and its inherent capacity to handle scattered and noisy experimental data (Figure 6) [102]. All these advantages come with a detrimental increase in computational resource requirements, given by the repetitive solution of the forward problem. To reduce computational expense, some formulations have proposed the use of surrogate simpler models for the forward problem at the initial stages of the optimization process [103,104]. Some statistical tools based on Bayesian data analysis and inference have been implemented to improve the performance of iterative inverse methods for cases where the error distribution of the measured target is known or can be safely assumed [105].

The core concept of iterative inverse methods is the solution of an optimization problem that drives the solution into reproducing the target data with the smallest possible error. The definition of an optimization problem requires the selection of an appropriate target function to minimize, an optimization algorithm suited for the particular characteristics of the forward problem and optimization parameters, and the implementation of parameter constraints to point and restrict the convergence of the algorithm into desirable outputs. In this section, we briefly discuss the most common optimization target functions, algorithms, and constraints used on inverse problems in biomechanics.

#### 4.2.1. Target Function

Most inverse studies implement a single target function for optimization, although optimization of multiple targets is feasible. The target function is often defined in terms of an error between the forward problem output and experimental measurements. Since the target function is defined as an error to be minimized but not exactly reduced to zero in a point-wise fashion, the iterative inverse method can converge to reasonable solutions even if the measured data are scattered and affected by random error.

The selection of an adequate target function must comply with at least two conditions. First, the target function must be compatible with the solution space of the forward model; otherwise, convergence may never be achieved, e.g., pulse wave velocity is incompatible as a target with CFD models assuming rigid walls. Second, the target function must be representative of all the aspects of the modeled phenomena, e.g., a target function based solely on output flow estimation is not adequate for FSI models since the contribution of the elastic behavior of the wall can be miscalculated. In FSI studies, pulse wave velocity or multiple target functions dealing with pressure and flow velocity are more reasonable options [106].

##### Structural Tissue Mechanics

First attempts to assess patient-specific mechanical properties of blood vessels were based on the knowledge of pressure vs. volume or pressure vs. area changes. Nevertheless, this unidimensional information can only be used to fit simple models that assume material homogeneity and isotropy, which departs from the known complexity of myocardium and arterial tissue [107]. The least-squared error to a pressure-volume curve as an optimization target increases the number of comparison points and allows the fitting of non-linear material models [108]. However, pressure–volume curves can only be measured in practice through invasive catheterization and are not generally available on a patient-specific basis. Alternatively, such data could be obtained from other sources such as normalized models with self-similarity or statistically obtained atlases. For example, Klotz et al. found that normalized pressure–volume curves of the left ventricle (LV) have a consistent profile, regardless of etiology across large mammal species [109]. This normalized pressure–volume function has been extensively used for forward and inverse analyses of the LV when direct pressure measurements are unavailable [110].

Some studies use the high-resolution information from MRI or computed tomography (CT) to obtain accurate geometric models of arteries at diastole and systole. The diastolic configuration is discretized into the mesh, which is then mapped into the systolic geometry by incorporating kinematic assumptions such as negligible axial and torsional displacements [111]. Then, the target optimization function can be set to minimize the simulated to mapped displacement errors. Due to the non-uniform distributions of nodal displacements, this technique allows the estimation of heterogeneous distribution of stiffness from anisotropic material models [112]. To avoid the requirement of node-to-node correspondence, and the related displacement assumptions while using geometric (non-kinematic) information, some authors proposed the minimization of the least-square-error of the distance between the loaded/deformed simulation mesh to the surface of the segmented anatomy model at systole, or systolic shape matching [113,114].

The explicit displacement field distribution made available by ultrasound speckle tracking, MRI tagging, or DENSE MRI, allows for defining a more direct target function by minimizing the least-squared error of the nodal displacement between the simulation results and the image-based measurement. The direct comparison of simulation-to-measured displacements can be achieved by locating mesh nodes in the location of speckles/tags/voxels, or by interpolating the measured displacement field into the mesh [14]. To reduce the effect of noise, some authors prefer defining the target function in terms of a region-wise averaged strain, instead of displacement distributions [115]. By averaging the strain field over a region, the effect of the noise is dampened. However, this requires an adequate discretization of the domain on regions of similar boundary conditions and material properties, while keeping discretization regions small enough to produce a smooth distribution of strain estimates. The use of region-wise averages of strain is widely used in the analysis of heart mechanics, and there is even a standardized discretization of the left ventricle. However, defining adequate regions for smaller and thin vessels is challenging [13]. Furthermore, defining the target function solely on strain measurements rules out the effect of possible translational/rotational rigid body motions.

The use of stress fields as target data can potentially reduce solution multiplicity on the fitting of material parameters, and yield results that more accurately describe the mechanical behavior of the tissue under study. The definition of such targets, however, requires a priori knowledge of the boundary loads, and resulting stress distribution within the deformed domain of study. In practice, some controlled in vitro experiments have successfully applied inverse models with stress-based targets by having accurate measurements of forces and deformations in three orthogonal directions on samples of reduced size [18,116,117]. The definition of stress-based targets for patient-specific in vivo applications could be extremely beneficial to improve the accuracy and uniqueness of the solution. However, it would require the implantation of load sensors on and within the tissue of interest. Given that in vivo tissue samples are not isolated, as they are in controlled in vitro experiments, further assumptions on material behavior and boundary conditions are required.

##### Fluid Mechanics and FSI

Cardiovascular catheterization pressure measurement is considered the reference standard on patient-specific hemodynamics, as it constitutes a direct assessment of pressure and dimensions within the blood vessels or the cardiac cavities using high-accuracy transducers. When available, most inverse models of computational fluid dynamics use a least-square-error of the time-dependent pressure function as the optimization target, while image-based flow data is used as boundary conditions [103,118,119]. Models incorporating FSI can instead use the pulse wave velocity as an optimization target that accounts for both the hemodynamics and elastic properties of the vessel [120]. The carotid-femoral pulse wave velocity is considered the gold standard for systemic arterial stiffness assessment, which is calculated as the patient-specific distance between the carotid and femoral artery and the time delay between the pressure wave measured at those locations. Local estimations of pulse wave velocity can also be obtained from invasive catheterization and by flow-to-area ratios from doppler ultrasound or phase-contrast MRI [121]. However, CFD cardiovascular modeling often relies on rigid wall simplification which significantly reduces the computational cost of the forward problem. Furthermore, cardiac catheterization is an invasive procedure and may not be available, so target pressure data is either non-available or non-reproducible owing to model limitations. In these cases, either least-square-error of nodal velocity between simulation results and 4D flow MRI assessment or branch flow distributions have been used as target functions [122].

#### 4.2.2. Optimization Algorithms

The development of algorithms for numerical optimization is a broad and active field of research. It is not the aim of this article to carry out a comprehensive review of all the available optimization techniques, but rather to list the methods most commonly used in the field of biomechanics, providing a rationale for their selection with specific problems. In the following sections, we loosely follow the classification proposed by Kochenderfer and Wheeler based on the characteristics of the target function [123]. Given the nature and complexity of inverse biomechanics problems, we only consider optimization algorithms that deal with continuous variables and multiple optimization parameters.

##### Updating by Differentiation of Target Function

In those cases where the target function is continuous and derivable, derivative information can be used to estimate the descent path towards the minimum. First and second-order algorithms refer to optimization methods that incorporate numerical evaluations of the local Jacobian and the Hessian matrix, respectively. To reduce the risk of convergence to local minima, stochastic sampling of these derivatives is incorporated.

First-order algorithms can only deal with relatively simple problems and are not suited for inverse biomechanics. However, they are used in other relevant applications, such as the automation of image processing and segmentation for the generation of geometric models [11]. Second-order algorithms have been used in the solution of inverse arterial and myocardial mechanics for the estimation of anisotropic material constants. The most commonly used are the Levenberg–Marquardt [34,107], Broyden–Fletcher-Goldfarb–Shanno (BFGS), limited BFGS (L-BFGS), and sequential quadratic programming [5].

##### Updating with No Differentiation of Target Function

Some optimization algorithms do not require derivative information of the target function to operate. These methods are not as fast as gradient-based counterparts when applied to derivable functions. However, they are advantageous in cases where the functions are not derivable, there are regions with invalid solutions or singularities, the function response is noisy, or the target presents multiple local minima. Since the target functions on simulation-based inverse problems are not analytical functions, but instead, are the simulation outputs, it is prone to some numerical problems, e.g., non-valid solutions due to forward problem divergence. In consequence, most recent inverse method developments have incorporated gradient-free algorithms. The most common algorithms can be classified into two groups direct methods and population methods.

Direct methods incorporate deterministic algorithms based on patterns or geometrical constructs for sampling the domain and carry a direct comparison of the target function value. This comparison is then used to define the location of the next sampling point. Powell and Nelder–Mead algorithms have been particularly popular in biomechanics applications and inverse analyses [124,125].

The main feature of population methods, in contrast to direct methods, is that the initial seed is not a single point in the parameter hyperspace but a pool of candidate optimum solutions. On each iteration, a new pool of candidate solutions is generated by altering the input parameter values following different recombination rules from parent candidates and stochastic variations. Then, each new candidate is evaluated and a new pool is selected to build the next generation. These algorithms have proven to be particularly useful when dealing with noisy target functions, and with multiple local minima. The popular genetic algorithms and particle swarm methods stand out due to their multiple applications, including the solution of inverse problems [117,126–128]. These methods require intensive sampling, so they are contraindicated for the solution of inverse problems when the forward problem is computationally expensive [15].

##### Statistics-Based Methods

Some statistical methods applied in the field of biomechanics rely on the use of Bayesian inferences, also known as inverse probability. Unlike the methods described previously, Bayesian inference-based methods provide not only an estimation of the parameters to be fitted but also a confidence interval for such values. The method requires a set of measured data along with its probability distribution (which can be often assumed normal due to random experimental error), a predictive model, and a prior probability distribution for the model parameters. The latter can be estimated from previous experiments and published data or can be simply assumed as uniform within a given range. Then, a selection of parameter combinations is used to run the prediction model and compare it to the experimental data. Finally, the Bayes theorem is used to produce a map for the probability of the model that reproduces the experimental data in a parameter hyperspace. This map is used to update the prior parameter probability distribution to iteratively repeat the process [129].

There are many different computational implementations, some of the methods used on patient-specific inverse problems are the Gaussian process regression, Kalman filters in its many variations, and linear-quadratic-Gaussian estimations. These methods differ mostly on how the sampling is carried out, how the parameter probability distribution is assumed or calculated on each step, and how the model predictions and experimental measurements are weighted to determine the converged parameter solution [130–132].

#### 4.2.3. Constraints

A series of constraints can be implemented on the optimization algorithms to restrict solution spaces and parameter values. These constraints can be used to ensure the physiological and physical meaning of the results and to funnel down solution multiplicity. One of the most relevant constraints required for material parameter estimation on tissue mechanics is compliance with the second law of thermodynamics. One of the required conditions for this compliance is that the strain energy function must be positive convex, which restricts the relative value of material parameters [24,133].

The assumption of material incompressibility is another common constraint imposed on cardiac and arterial wall mechanics. Full incompressibility introduces singularities to the solution of numerical formulations; therefore, nearly incompressible behavior is enforced by restraining the relative values of material properties. However, experimental and in silico evidence have shown that cardiovascular tissues are compressible to some degree and that myocardial volume varies throughout the cardiac cycle [134]. The most recent in vivo measurements of myocardium compressibility in human and large-mammal animal models agree on estimating peak compressibility between 1% and 20% [134–136] Moreover, it has been shown that the accuracy of heart-mechanics models is significantly increased if this compressibility effect is considered [137]. Thus, the incompressibility constraint is a reasonable yet rough approximation that must be carefully considered in simulation analyses [134,138,139].

Microstructure-based models allow the introduction of physiologically and structural meaningful constraints to material parameters, e.g., maximum possible fiber stiffness, or maximum cellular volume fraction. Inequality type constraints can restrain material parameters within expected physiological ranges. Inequality relations between model parameters can also be introduced to address structural component differences, e.g., collagen fibers are typically stiffer than elastin fibers. In the study of FSI inverse problems, pulse wave velocity is constrained by the maximum possible speed of sound on the liquid media, and some authors have introduced constraints on the maximum volume change of the fluid-solid domain [140].

In addition, constraints can also be introduced to promote numerical stability of the solution, or to smooth the solution when the parameters to be fit are temporal or spatial distributions, e.g., the first-order Tikhonov regularization functional has been used in the estimation of heterogeneous material parameter distributions [141].

### 4.3. Summary

In Table 3 we summarize the highlights of direct and iterative solution methods of inverse problems.

## 5. Medical Imaging-Based Kinematics

Early attempts to use medical imaging to assess the stiffness of blood vessels relied on the measurement of the luminal area change between diastolic and systolic configurations. This area change is used in several clinical risk markers, such as the β-index, that have shown a good correlation with the occurrence of certain cardiovascular pathologies such as atherosclerotic damage, hypertension, diabetes, and Marfan syndrome, as well as to tobacco exposure, obesity, aging, and other risk factors [142–144]. However, the predictive capabilities of these factors are inconsistent among different arterial locations and pathologies, most likely due to the oversimplification of the problem without any account of vascular mechanics [142].

Multiple previous studies have considered inverse problems applied to in vitro markertracking kinematics of surgical and cadaveric tissue samples [145,146]. In these works, direct or fluid-driven mechanical loads are applied to the tissue sample to induce controlled deformation through an in vitro experiment setup. Physical or digital markers are fixed to the samples, and their displacement is captured by high-speed, high-resolution cameras. These studies are less affected by resolution limitations and noise than in vivo studies and can be applied to structures that are difficult to capture with medical imaging such as heart valve leaflets [147]. Some notable drawbacks of in vitro testing of explanted tissue include neglecting active contractility, loss of in vivo boundary conditions, potential tissue damage during excision, experimental setup and marker placement, and degradation of the living tissue after extraction.

In vivo medical imaging has evolved to provide not only anatomical geometric information but also detailed kinematics measurements. The accuracy and availability of these techniques are limited by image resolution, signal-to-noise ratio (SNR), the occurrence of artifacts, and practical obstacles related to testing costs and health hazards [122,148–151]. In the following subsections, we review the available techniques for assessing in vivo image-based kinematics for tissue deformation (Table 4) and blood flow (Table 5), fundamental principles, typical image resolution, and some specific applications.

### 5.1. Ultrasound Technology (US)

**Figure 7.**
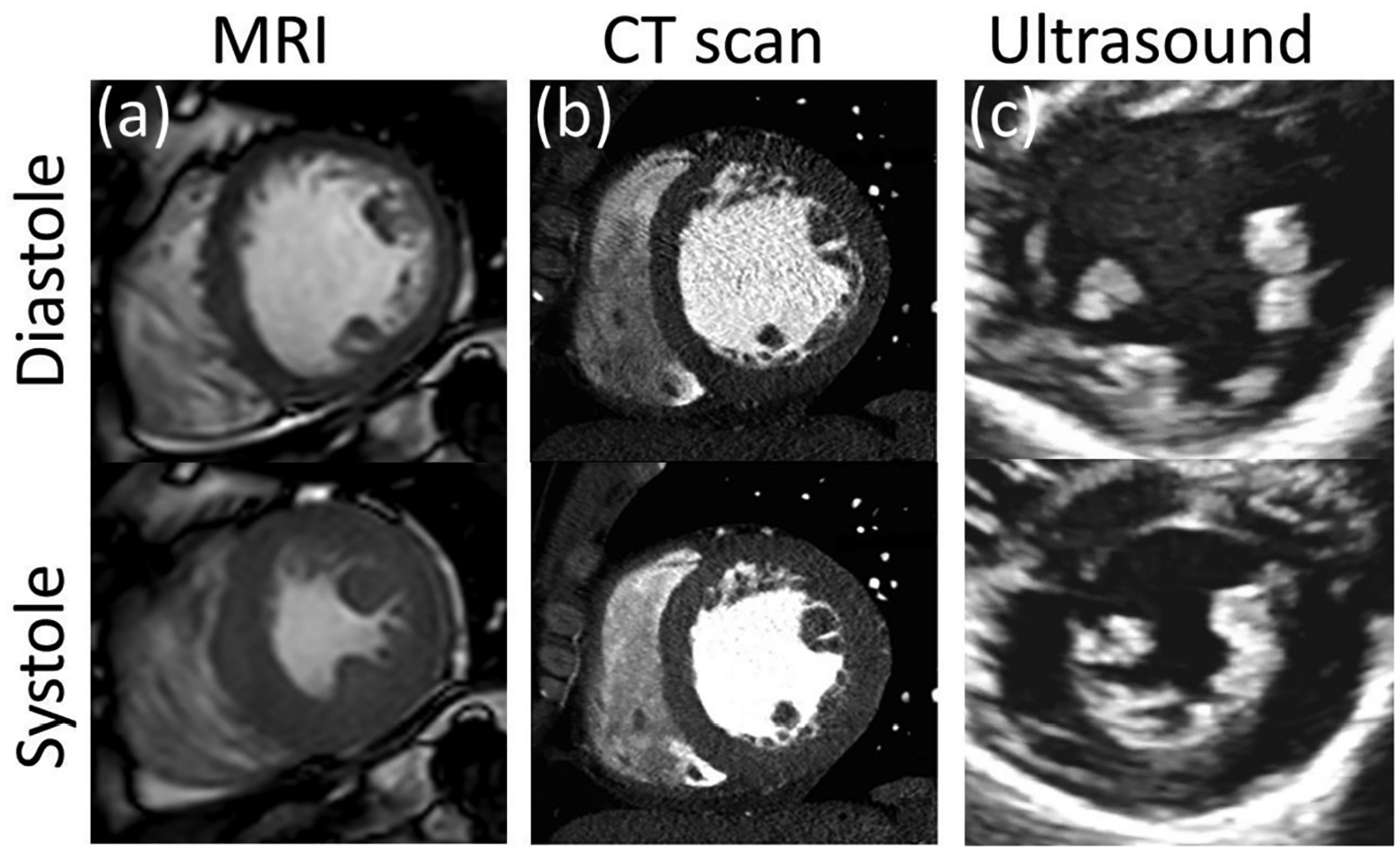
Resolution comparison of left ventricular myocardium at diastole and systole with clinical grade (**a**) MRI, (**b**) CT (Reprinted/adapted with permission from Ref. [158], 2019, Korean Society of Echocardiography, open access), and (**c**) 2D ultrasound.

Ultrasound (US) uses high frequency (2 to 15 MHz) acoustic waves to create real-time 2D in vivo images of tissues, organs, and blood pools using piezoelectric transducers. As in any wave, higher frequencies are associated with smaller wavelengths, higher penetration power, and improved image resolution [152,153]. Volume rendering from ultrasound images has led to three-dimensional, time-resolved ultrasound (4D US), and real-time imaging [154]. US is relatively inexpensive, portable, and safe, so it has become a customary tool in many clinical applications such as anesthesia, critical care, prenatal care, and pain management. Its application to cardiology, commonly known as echocardiography, was introduced in the 1950s, and currently is the more ubiquitous diagnostic tool to assess cardiovascular structure and function. With an approximate lateral resolution of 1 mm/pixel, this technique allows the estimation of heart chamber size, valve structure, identification of structural abnormalities such as seen in congenital heart defects (CHD), and determination of systolic and diastolic function (Figure 7c) [155]. Nevertheless, echocardiography presents some intrinsic limitations regarding accuracy and repeatability, particularly in patients with complex flow patterns related to congenital heart disease, aortic regurgitation, or dissection, in which case it is recommended to complement the study with other imaging techniques [156]. Intravascular ultrasound technology (IVUS) was developed following the principle that accuracy and resolution are improved as the transducer is closer to the tissue of interest. This technology involves placing a miniature ultrasound probe at the end of a catheter and then introducing the catheter into the vessel of interest in order to resolve the surrounding structures with greater detail than allowed by standard external US. This invasive technique is mostly used to study the conditions and progression of atherosclerosis in patients with coronary and carotid artery disease [157]. US technology can also provide blood flow and tissue kinematic information through the use of echo-Doppler and speckle tracking techniques.

#### 5.1.1. Echo and Vector Doppler

Echo Doppler estimates the velocity of blood and tissue through the use of the Doppler equation. By measuring the frequency shift from the original ultrasound wave and the reflected echo, the local velocity can be determined. The main shortcoming of this technique is its dependence on the angle between the original ultrasound wave (position of the transducer) and the displacement direction, which can introduce large intra- and inter-observer variability. Dependency on the transducer angle was solved by the introduction of vector Doppler techniques, which additionally provide in-plane velocity components. This is achieved by the simultaneous measurement of two doppler signals, either from two crossing beams from different transducers, or from a single transducer with two different in-plane receivers [159]. This technique is of great use in clinical practice for the qualitative assessment of blood flow and tissue displacement [160] and used in early studies of patient-specific hemodynamics to impose inlet and outlet flow boundary conditions [161,162].

#### 5.1.2. Speckle Tracking

**Figure 8.**
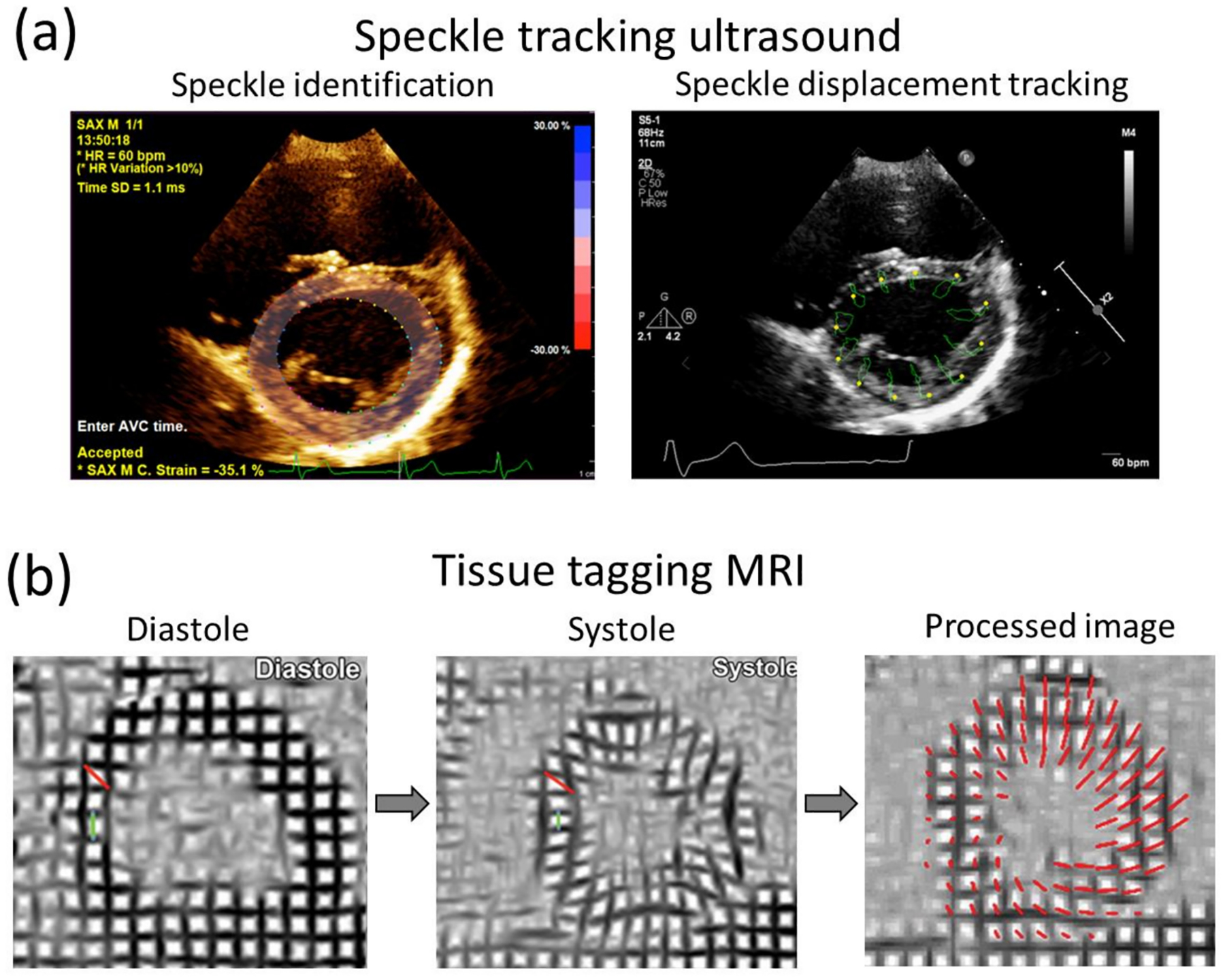

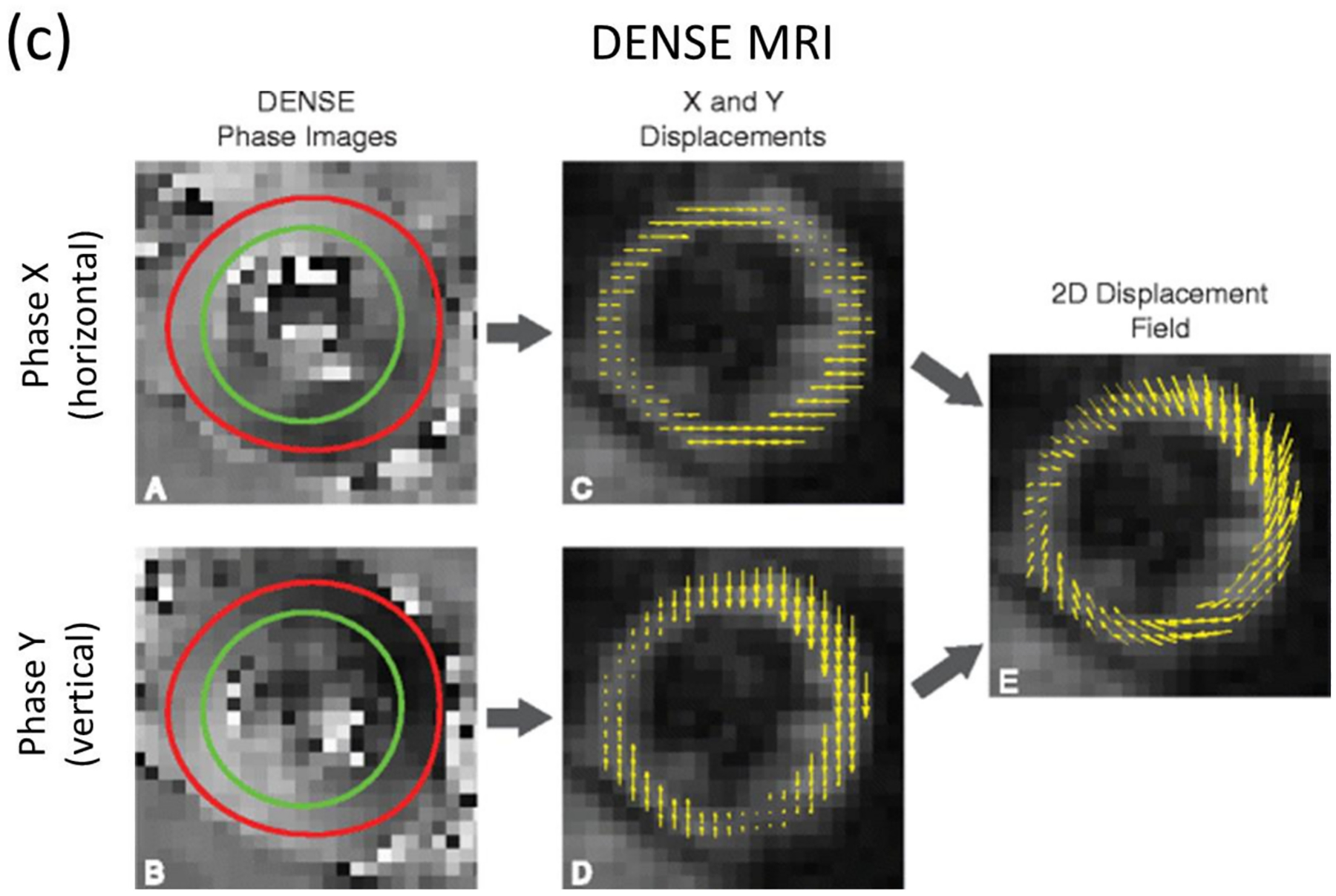
Examples of image-based kinematics of the left ventricular myocardium with (**a**) Speckle tracking ultrasound (Reprinted/adapted with permission from Ref. [169]. 2020, Alessandra M. Ferraro et al.; open access) (Left) Dots indicate speckle-kernel location and identification, (right) green lines indicate trajectory through the cardiac cycle. (**b**) Tissue tagging (Reprinted/adapted with permission from Ref. [170]. 2012, The Radiological Society of North America). Tissue tagging estimates kinematics by tag-to-tag tracking from diastole to systole. Transversal (green lines) and diagonal (red lines) tag-to-tag dimensions are measured at diastole (left column) and systole (middle column), their difference can be used to measure displacement and deformation (red lines in right column). (**c**) DENSE MRI (Reprinted/adapted with permission from Ref. [171]. 2015, Wehner et al.; licensee BioMed Central, open access). DENSE MRI resolves pixel-wise displacements by processing phase data for each direction. Red and green contours represent segmented luminal and adventitial boundaries, yellow arrows represent the phase-encoded displacement.

Speckle tracking is a relatively novel technique developed during the early 2000s for the measurement of tissue 3D displacement and deformation. Speckles are defined as image features/spots generated by the acoustic response of tissue fibers to ultrasound signals. Single speckles are analyzed in identifiable kernels that are followed along the cardiac cycle. Postprocessing techniques allow the averaging of kernel displacements over several cardiac cycles to reduce the effects of noise [163]. The spatial and temporal resolution of speckle tracking is remarkable, providing hundreds of frames per second for pixels of <1 mm size [164]. This resolution allows not only the study of the myocardium but also the mechanics of arterial walls and aortic aneurysms [165,166]. Displacement measurements are limited by kernel size (~1 mm) and show reproducibility issues common to any US-based technology (Figure 8a) [167,168].

### 5.2. Magnetic Resonance Imaging (MRI)

Magnetic resonance imaging (MRI) offers superior quantitative utility compared to ultrasound as it offers high-resolution 2D and 3D visualization of the heart and major arteries referenced to a fixed coordinate system, providing greater accuracy for anatomic and volumetric assessment of heart chambers and wall thickness [160]. In addition, various MRI sequences have been specially designed to assess other valuable data such as fiber orientation, tissue displacement, and blood velocity [172,173].

The fundamental principle of this technology consists of the use of magnetic fields to align hydrogen protons in the body. After the magnetic field is interrupted, the protons return to a lower energy state by emitting radio signals that can be captured and utilized to create imaging data. For clinical applications, base magnetic fields with strengths ranging between 1.5 to 7 T are used to excite the protons to a base level. Then, a second time-varying radiofrequency magnetic field is used to induce changes in tissue magnetization. Tissues with different hydrogen-protons content will respond to the oscillating radiofrequency with different characteristic responses, which can be used to resolve various tissue components [174].

The first reported human magnetic resonance image dates from 1977 and was a single image that required 5 h to capture [175]. Nowadays, an MRI image can be captured in a single breath-hold with a resolution under 1 mm/pixel and temporal resolutions of 25 frames per cardiac cycle (Figure 7a). This noninvasive technique generally poses minimal risk to patients unless they have non-compatible ferromagnetic implanted devices or other internalized materials, or they have complications related to the contrast agents needed for some of the MRI modalities. Due to the confined space within an MRI scanner, the test can generate anxiety and discomfort for patients with claustrophobia. Given the expense of the equipment, maintenance, and required staffing, it does not have as wide an availability as US, particularly in smaller medical facilities.

There are several options for MRI-based assessment of kinematics of both cardiovascular soft tissues and blood flow. Four prominent examples will be discussed below, including: MRI tagging and displacement encoding with stimulated echoes (DENSE) for tissue displacement, and phase contrast and 4D flow MRI for blood flow quantification.

#### 5.2.1. Tissue Tagging

This technique, first introduced by Zerhouni et al., in 1988, was specifically designed to quantitatively assess the transmural motion of the myocardium [176]. Image markers, or tags, are created by locally perturbing the magnetization of the tissue, either by selective radiofrequency saturation sequences or through modulation of the magnetization vector by gradient fields. Tags are created on a thin section orthogonal to the image plane at diastole, then followed by regular time-resolved imaging. Electrocardiographic gating is used to consistently apply the tagging radiofrequency at diastole. Early works reported decrement of tag resolution at systole as the magnetic saturation exponentially decays over time; nevertheless, this problem is palliated with the use of larger magnetization energy [177]. Ibrahim et al. showed that tag lines were still clearly identifiable at the end of the cardiac cycle on human hearts with a 7 T MRI scan [178]. Special radio-frequency sequences, such as spatial modulation of magnetization (SPAMM) and delays alternating with nutation for transient excitation (DANTE) [179,180], allowed the creation of 2D orthogonal tagging grids that facilitate the kinematic analysis. Tag sizes can be only as small as the pixel-size resolution (>1 mm) with typical tag spacing of about 5 mm (Figure 8b). The technique allows for 25 to 30 images per cardiac cycle, requiring about 20 s of scan time per tagging sequence [170,181]. Multiple studies on phantoms have shown this technique to be superior to US speckle-tracking in terms of accuracy and repeatability. Due to the spatial resolution limitations of this technique, it has only been successfully applied to study the kinematics of relatively thick tissues such as the myocardium, skeletal muscle, lung tissue, and the tongue [182].

#### 5.2.2. Phase-Contrast

Phase-contrast (PC) MRI utilizes the intrinsic phase of the magnetic signal to retrieve kinematic information. When a magnetic field gradient is applied to a body, the spins of the protons develop a phase shift that is proportional to its relative velocity. When two consecutive and opposing gradients are applied, stationary protons will show no phase shift. However, moving protons will show different degrees of phase shifting as they change their position with respect to the gradient [183]. This information can then be used to encode the velocity and displacement of protons.

Since the kinematic information is encoded in the phase information, and thus is independent of image markers, this technique allows measurement at scales below pixel-size resolution [12,149]. However, the technique is sensitive to Eddy currents, concomitant gradients (Maxwell terms), and nonlinearities in the gradient field. These effects increase the signal-to-noise ratio (SNR) and produce offset errors that are both spatial- and time-dependent. SNR has been shown to increase with the magnetization energy and has been estimated to range between 20 for 1.5 T scanners to around 60 for 7 T scanners. Offset error correction requires the implementation of rectification algorithms in the postprocessing stage [184,185].

##### 2D CINE PC-MRI and 4D Flow MRI

The application of consecutive opposite magnetic field gradients is known as a bipolar gradient. After a bipolar gradient is applied, the net phase shift of static protons is zero, so only the mobile protons will show a phase shift. From the latter, faster protons will experience a greater difference in applied gradients as they physically move longer distances than slower protons, which in turn produces greater phase shifts. The end result is that the phase shift is proportional to the proton velocity. However, because phase angles are limited (from 0 to 2 π), only a certain range of velocities can be directly quantified [183]. That is, for a given gradient, there is a maximal velocity that can be measured before aliasing occurs, called the encoding velocity (VENC). Encoding velocity is inversely proportional to the magnitude of the gradient; thus, by manipulating the strength of the gradient, it is possible to manipulate the range of velocities that can be encoded. Setting the encoding velocity is a tradeoff between the risk of aliasing and the minimum measurable velocity by the discrete scale [186].

Standard 2D Cine PC MRI, typically applied to estimate through-plane velocity, has become part of clinical practice in the treatment of cardiovascular disease, specifically, for the calculation of flow in large arteries and their main branches, cardiac output, and quantitative assessment of regurgitation and retrograde flows. This technique is also used for the qualitative assessment of flow patterns in large arteries and heart chambers. PC MRI data is usually recorded in DICOM format images with 8-bit or 16-bit pixels, that is 256 or 65,536 possible discrete levels, respectively. Phase data is typically encoded within 4095 values for the whole 0 to 2 π range, with pixel sizes around 1.5 mm [187].

**Figure 9.**
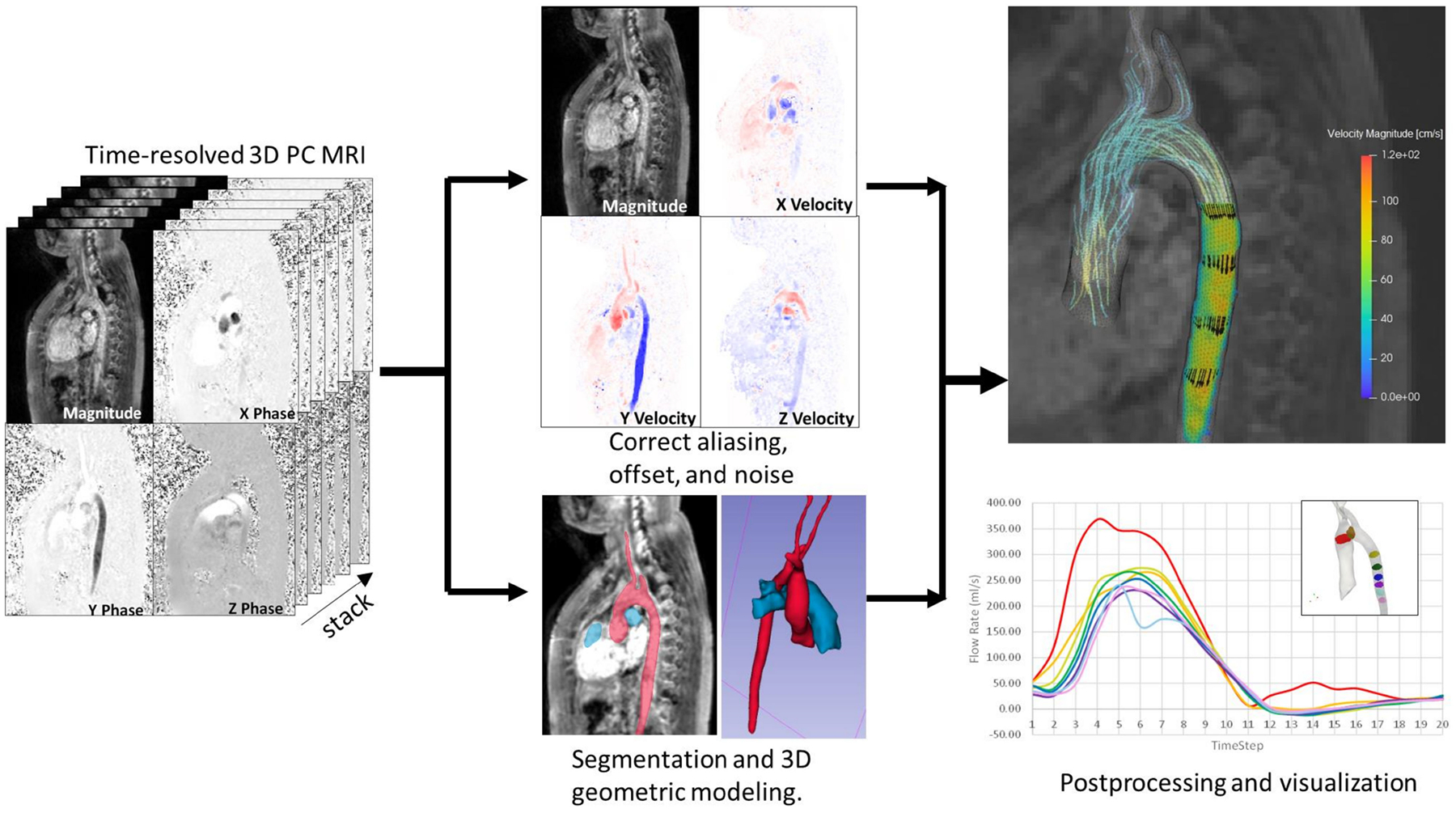
Processing pipeline of 4D flow MRI scans. (**Left**) Velocity-sensitive phase images are generated by 3D velocity-encoding subtracted from reference images. (**Middle**) Velocity estimations are corrected for errors due to noise, aliasing, and eddy currents. A 3D segment is created to define the region of interest. (**Right**) Velocity data are postprocessed to produce hemodynamic factors and useful plots and visualization.

However, standard 2D PC MRI can only provide the dimensional component of velocity perpendicular (through-plane) or parallel (in-plane) to the imaging plane, and thus is inadequate to estimate relevant hemodynamic metrics requiring three-dimensional flow information, such as vorticity and wall shear stress [188,189]. The logical evolution of this technique led to 4D flow MRI, which allows the volumetric and temporal resolution of three orthogonal components of velocity. This is achieved by applying consecutive bipolar gradients to three orthogonal directions on stacked planes. This requires the collection and processing of a significantly greater amount of data (three spatial dimensions and three velocity directions over several timesteps through the cardiac cycle), thus requiring special approaches to keep reasonable scanning times. Some hardware improvements include multi-receiver channels, phased-array coils, and parallel imaging technology. Other developments are related to improving the efficiency of data sampling, and averaging over several cardiac samples, namely radial undersampling, kt-GRAPPA, kt-BLAST, and kt-SENSE [190]. Additionally, the convex gradient optimization technique offers improved resolution and accuracy while maintaining the essential characteristics of velocity encoding [191]. For thoracic and abdominal applications, 4D flow scanning times range from 5 to 15 min, with voxel sizes of around 2.5 mm and temporal resolutions of 25 datasets per cardiac cycle (Figure 9). This technique has proven its value through many different in vivo patient-specific studies of normal and pathological hemodynamics in the heart [192], aorta [58,193], pulmonary artery, and complex single ventricle circulation [194–196]. Other applications include the evaluation of drug treatment effects [197] and surgical intervention outcomes [198–201].

##### Displacement Encoding with Stimulated Echoes (DENSE)

DENSE MRI is a modified version of PC MRI that improves phase contrast to measure slow velocity displacements while maintaining moderate gradient magnitudes, thus allowing the kinematic measurement of slow-moving tissue. This is achieved by manipulating the spin phase with stimulated echoes [149].

DENSE MRI was introduced in 1999 by Aletras et al., for the study of myocardial mechanics [149]. Since then, multiple developments have been proposed to optimize its assessment of human myocardial kinematics [202–204], minimize the effects of artifacts and breathing [205,206], and automate the unwrapping of DENSE MRI phase data [207]. Some potential clinical applications of DENSE MRI include the identification of biomarkers of early cardiac dysfunction [208,209], assessment of the response to cardiac resynchronization therapy [210,211], and identification of infarct transmurally for early postmyocardial infarction [212]. This technique has also been applied to assess the heterogeneous displacement, stretch, and circumferential strain around the aortic wall at different locations along its length [10,13,213,214]. Notably, a recent in vitro validation study of aortic DENSE MRI on wire-embedded polymer aortic phantoms revealed a final mean regional error in the quantification of the circumferential strain of <1% strain [215]. The potential for measuring in vivo shear and radial strains of the aortic wall has also been explored, though its repeatability is significantly less than the quantification of circumferential strain due to the thinness of the vascular wall [216]. Beyond the heart and aorta, other applications of DENSE MRI include the dynamics of the human brain and the cerebrovasculature [217].

The image resolution of typical cardiac DENSE MRI applications is around 2.5 mm/pixel; however, recent advances with the use of spiral k-space sampling DENSE MRI allow resolutions down to 1.3 mm/pixel to assess the kinematics of arterial walls [10,13]. Since the displacement is encoded in the MRI phase data and thus does not depend on tag-line parameters or tracking of image features, displacement is resolved at a scale below pixel size, with reported displacement uncertainties of approximately 0.09 mm [150]. Comparative studies on controlled in vitro experiments with gelatin phantoms, and in vivo strain measurements on human myocardium showed that DENSE MRI provides better accuracy and reproducibility than tissue tagging (Figure 8c) [181,183].

#### 5.2.3. Other Relevant MRI-Based Scanning Modalities

Magnetic resonance imaging can also provide relevant information for inverse modeling other than anatomic and kinematic information. In this section, we briefly review MRI sequences that allow the resolution of tissue structure. The image-based resolution of tissue structure and compositional heterogeneity can be used to tailor constitutive models to the volumetric share and orientation of fibrous structures. With regards to patient-specific modeling of cardiovascular disease, these techniques could help identify the location, extension, and severity of lesions, and thus more accurately divide the patient-specific anatomic models into unique regions with particular sets of mechanical properties. By supplying such patient-specific material heterogeneity as a prescribed input, the accuracy, convergence time, and solution multiplicity of inverse models of cardiovascular disease could be significantly improved.

Spin-to-lattice, and spin-to-spin relaxation times, also known as T1 and T2, respectively, are common MRI parameters typically used for highlighting the difference between fat and water. By definition, T1 is a shorter relaxation time than T2, so T1-weighted images highlight fat structures with large proton density, whereas T2 weighted images highlight both fat and water-rich structures. These sequences are commonly used in clinical practice to resolve scar tissue, blood pools, and edemas. Rapid T1 and T2 mapping combines both measurements to resolve an estimation of the extracellular volume fraction that has shown to be a robust marker for several cardiomyopathies, with a strong correlation to histological measurements [218].

Diffusion tensor MRI (DT MRI) is an imaging sequence that uses similar principles to PC MRI. With this technique, special magnetic gradients are designed to cancel out the signal from static water molecules while preserving the magnitude and orientation of moving molecules. Within tissues, water molecules diffuse by Brownian thermal motion, and in fibrous structures, this diffusion occurs preferentially in the fiber orientation. This technique has been mostly applied for the imaging of the white matter and axon orientation in the brain, and more recently to resolve myofiber orientation in the heart [53].

Other MRI-based techniques, such as gadolinium-enhanced MRI and perfusion tests, have been developed to specifically image cardiovascular scars, thereby allowing the quantification of lesion severity. Gadolinium is a contrast agent used to increase the SNR of MRI. The cellular membranes of healthy cardiomyocytes are almost impermeable to gadolinium contrast agents. As a result, following intravascular injection, gadolinium perfuses throughout the myocardium via the branches of the coronary arteries while being excluded from the intracellular space of viable cardiomyocytes due to the impermeability of cell membranes. For this reason, gadolinium can be used to measure the extracellular volume fraction of healthy myocardia from T1 mapping sequences. When myocardial cell membranes are ruptured, as is seen in infarction, a larger portion of gadolinium is accumulated. The contrast can now occupy the no-longer enclosed intracellular space, allowing the assessment of the location and severity of cell-rupturing injuries [219].

The MRI perfusion stress test can assess the severity of coronary artery insufficiency. The quality of blood perfusion into the cardiac wall is resolved through the use of contrast agents at rest and under stress conditions. Increased cardiac stress state can be induced by either exercise or the use of pharmacological stressors. Pharmacologically induced stress is preferred over exercise-induced stress as it renders more reproducible results and is easier to implement in clinical practice [220]. Typical pharmacological stressors include vasodilators (adenosine or regadenoson) or chronotropic inotropic agents (dobutamine). This technique exposes the patient to hazards associated with the use of contrast agents and pharmacological stressors and is generally reserved for patients with confirmed coronary artery disease [221].

### 5.3. Computerized Tomography (CT)

CT consists of a mobile X-ray source that rotates around a focal point to produce scans from different angles. The result is a high-resolution stack of 2D images that can be time-resolved. The use of intravascular contrast agents is common for studies of the vascular system to improve the visibility of the blood vessels. CT scans can provide better resolution than all the other techniques described above with pixel sizes of about 0.5 mm (Figure 7b). There is no special feature to assess kinematics from CT scans, although its superior temporal and spatial resolution has been used to measure the dynamic change in cross-sectional area and shape of blood vessels during the cardiac cycle, from which homogenized values of circumferential strain for a given cross-section can be estimated. From there, kinematics can be inferred from tracking a given anatomical feature or making reasonable assumptions about rigid body rotation and torsion [11,222].

The use of ionizing radiation makes this technique potentially hazardous; thus risk-benefit of a CT study should be seriously considered. This limits its use in serial follow-up, particularly in pediatric patients, to avoid repetitive exposure to radiation [223]. However, it avoids the risk of unknown or contraindicated implanted metallic object/devices associated with MRI and is typically capable of much shorter scan times than MRI, making it ideal for trauma or other acute emergencies.

### 5.4. Summary

In Table 6, we summarize the highlights of medical imaging techniques that provide kinematic data, and other useful information for inverse modeling.

## 6. Applications to Cardiovascular Medicine

One of the most relevant outputs of modeling in cardiovascular mechanics is the estimation of wall stress distributions, either in the vascular walls of major arteries or the myocardium of the heart. Mechanical stresses and strains, and their spatial and temporal evolutions, are measures of physiological significance because they may be potential indicators of myocardial and arterial function, may serve as risk stratification factors for tissue failure and rupture, and provide specific measures of the mechanical stimuli modulating biological adaptation. Applications of patient-specific models of cardiovascular mechanics include supporting diagnosis and risk stratification, providing visualizations and insights of deformation and loads on tissue structures, population-based analyses, and supporting and/or challenging mechanistic hypotheses of normal and pathological growth and remodeling.

For patient-specific forward problems in cardiovascular simulation, an anatomical geometric model is typically retrieved from medical imaging and discretized into a computational mesh over a domain of interest. Pulse pressure of blood is typically used as a load boundary condition acting on the luminal or endocardial surface, and any available image-based kinematic information is either used to prescribe velocity boundary conditions (for CFD analyses) or to validate the output of the simulations [224,225]. Forward simulations require the assumption of many parameters that cannot be directly measured or were not collected (e.g., myocardial and arterial wall composition and mechanical properties, blood properties, or focalized blood pressure measurements [226]). Due to these limitations, it has been argued that forward-simulation results should not be taken as absolute quantitative results, but instead, interpreted qualitatively and comparatively in terms of patterns, distributions, and trends of derived stresses and other relevant metrics [227,228]. This caution should especially be emphasized in pathological cases, where the normal physiological function is impaired and assumptions applicable to normal and healthy tissues do not hold [229]. Conversely, patient-specific data can be input into inverse methods to solve for the parameters that are unavailable or cannot be directly measured, potentially reducing the number of required assumptions and improving the ability of the model to fit the observed data. In the study of the cardiovascular system, inverse problems can provide patient-specific estimations of tissue properties, composition, local pressure gradients and stress distributions from image-derived wall deformation, and blood flow dynamics.

The development of patient-specific inverse analyses of cardiovascular mechanics has advanced considerably recently thanks to continuous technological improvements in imaging hardware and software, decreasing cost, increased imaging availability, improvements in image-based kinematics acquisition, and postprocessing, simulation engineering, and significant increases in computational power (Figure 1). Notably, the modern era of computationally robust image-based cardiovascular inverse modeling began with the study of animal models by the end of the 20th century. A pioneering work on in vivo image-based inverse modeling of cardiovascular tissue was published in 1995 by Moulton et al. This research on a canine animal model used a single slice MRI with radio-tagging to retrieve the anatomy and displacement of the short axis plane of the heart [107]. A non-linear error-gradient-based optimization algorithm minimized the least-square error of FEM simulated and MRI-derived strains, by iterating over the constants of the Fung material model considered without any muscular activation component. The boundary conditions were the trans-ventricular pressure measured from catheterization and the restriction of two degrees of freedom of a single computational node. An improved approach was proposed by Walker et al., who applied the inverse method to study the mechanics and properties of infarcted sheep hearts [230] and the effect of surgical intervention [231]. Therein, the authors employed MRI-based 3D models of the left ventricle and MRI tissue tagging to estimate the diastole-to-systole strain field. The latter was used as a target for fitting the material parameters through an iterative inverse formulation. The active contraction was simulated by a time-dependent homogeneous active stress model, and catheter measurements of ventricular pressure were used as boundary conditions. These studies found that fiber and cross-fiber stress are significantly larger at the infarct border zone relative to non-infarct regions. Additionally, the inverse model was employed to evaluate the benefits of diverse treatments and suggested that aneurysm plication decreases the myofiber stretch without compromising stroke volume, which the authors highlighted as one of the benefits delivered by such intervention.

These early works present all the elements of more recent medical image-based inverse analyses: an image-based kinematic target, an optimization algorithm, and a parametric function to be optimized to estimate in vivo case-specific information that cannot be directly assessed without an invasive procedure. These studies were limited by the available computational power at the time. Walker et al. reported a total of 16 h for each iteration of their forward cardiac model using a Silicon Graphics Octane II workstation with a capacity of about 250 MHz, which was a cutting-edge multiprocessor workstation at the time. Currently, the processing capacity of a desktop workstation is at least ten-fold greater (i.e., 3 to 4 GHz). Furthermore, many parallelization and cloud-computing options are now available to augment the speed of simulations. The technology is now mature enough for the evaluation of patient-specific inverse analyses on complex biomechanical models of clinical relevance.

**Figure 10.**
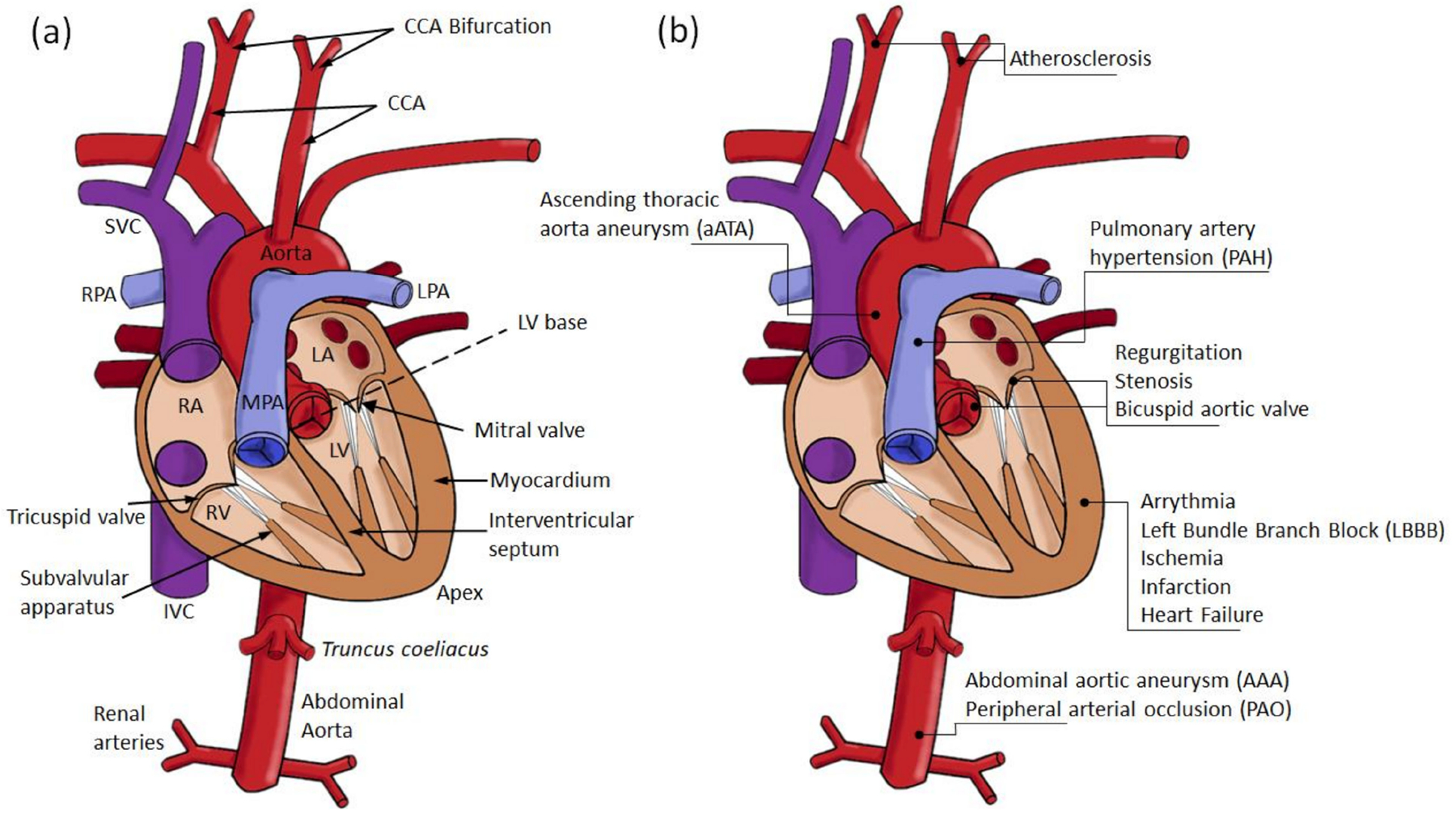
(**a**) Schematic representation of the human heart with anatomical references. (**b**) Location of human pathologies studied with inverse models. Acronyms: CCA, common carotid artery; IVC, inferior vena cava; LA, left atrium; LPA, left pulmonary artery; LV, left ventricle; MPA, main pulmonary artery; SVC, superior vena cava; RA, right atrium; RPA, right pulmonary artery; RV, right ventricle.

Though there are many instances of image-based inverse analyses on animal models and explanted tissues [137], in this review we aim to highlight the potential clinical applications of inverse methods. Thus, in this section, we present a detailed review of in vivo patient-specific inverse problems applied to elements of the human cardiovascular system along with a few pioneering and groundbreaking studies on animals. Figure 10 summarizes the anatomical references and location of focalized pathologies studied by the inverse-modeling applications reviewed herein.

### 6.1. The Unloaded Reference Configuration in Cardiovascular Mechanics

Blood vessels, in particular those of the arterial tree, function under physiological pressure load at all times and are axially pre-stretched; thus, none of the patient-specific configurations resolved by in vivo imaging is truly a stress-free or zero-strain configuration [232]. It is well established that image-based estimations of material properties and stress distributions are sensitive to the selection of the reference configuration. Furthermore, image-based in vivo estimations of material properties assuming the diastolic configuration as a zero-strain stress-free reference lead to significant disagreements with experimental measurements made on excised tissue [233]. That means an adequate selection of the reference configuration is key for the accurate solution of inverse problems of cardiovascular tissue mechanics.

The solution of an unloaded configuration from the deformed geometry, mechanical loads, and material properties is a classical inverse problem with existing direct and iterative solutions [234]. In patient-specific analyses, however, the material properties are also unknown. Thus, the solution to this problem requires the specification of at least two deformed and loaded states as input data [47]. In the case of myocardium, it is often assumed that the transition from unloaded to diastolic configurations is purely passive [235].

In this first subsection, we review previous contributions related to finding patient-specific unloaded and stress-free configurations without the estimation of mechanical properties. Since the methods described can be applied to any pressure vessel, we include developments regardless of the specific tissue application. Research works that incorporate the unloaded or stress-free configuration on the patient-specific estimation of tissue properties and loads are reviewed on the following sub-sections separated by the corresponding tissue of interest.

**Figure 11.**
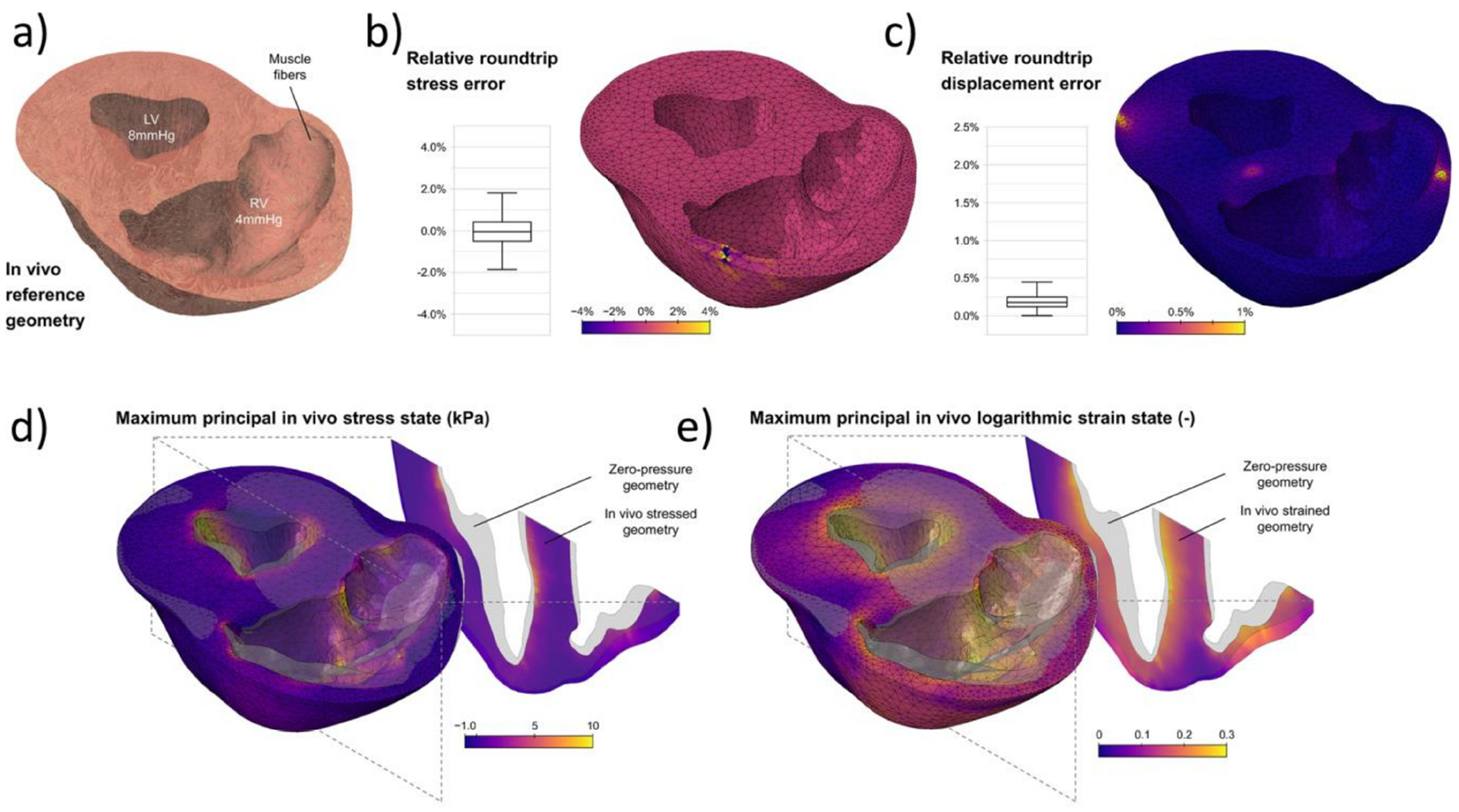
Results and accuracy of the direct inverse elastostatic problem implemented on a FEM solver and applied to a porcine biventricular model. (**a**) Reference loaded configuration reconstructed from MRI scans. (**b**) Relative stress error of the in vivo loaded configuration and solution of the forward inflation problem from the estimated unloaded configuration (roundtrip solution). (**c**) Relative displacement error the in vivo loaded configuration and solution of the forward inflation problem from the estimated unloaded configuration (roundtrip solution). (**d**) Colormap representation of the maximum principal stress distribution of the loaded configuration on top of the estimated unloaded configuration in gray shade. (**e**) Colormap representation of the maximum principal strain distribution on top of the estimated unloaded configuration in gray shade. (Reprinted/adapted with permission from Ref. [100]. 2018, Elsevier).

The direct inverse FEM formulation by Govindjee and Mihalic (c.f. Section 4.1) for the direct solution of the unloaded configuration was first applied to cardiovascular tissue by Lu et al. [111]. The inverse elastostatic approach was used to find the unloaded configuration of an abdominal aortic aneurysm (AAA), assumed to be loaded at a luminal pressure of 100 mmHg, and to behave as an isotropic hyperelastic material with population-averaged material constants. The authors concluded that the selection of diastole as the zero-stress reference leads to the overestimation of stress at systole. A similar approach was applied by Peirlinck et al., who incorporated the inverse elastostatic formulation as a module for the Abaqus FEM solver [100]. The method was applied to an iliac artery ideal model, an image-based porcine biventricular model, a human AAA, and a patient-specific 4-chamber heart model (Figure 11). The method was tested with hyperelastic and fiber-reinforced anisotropic material models. Material constants and pressure loads were imposed based on established reference values from the literature. The authors highlight the convenient modular implementation, computational efficiency, and solution uniqueness as the main advantages of their proposed method.

Several iterative methods have been specifically proposed to solve the zero-pressure configuration for blood vessels. One of the first contributions was proposed by Raghavan et al., who developed an optimization framework for an arbitrary parameter k such that the coordinates of the unknown zero-pressure reference geometry (***x***_0_) can be approximated by the difference of the in vivo deformed configuration (***x***_*i*_) minus k times the displacement produced by the pressure load on that configuration (𝒰), i.e., ***x***_0_ = ***x***_*i*_ – *k*𝒰. The main conceptual limitation of this method is the assumption that the backward deformation field is linearly related to the forward deformation field through the factor k. This method was then applied to estimate the unloaded configuration of a patient-specific AAA [236].

The backward displacement method was introduced by Rajagopal et al., in 2007, for breast biomechanics and by Bols et al., in 2013, for cardiovascular tissue [234,237]. This method solves the unloaded configuration using the fixed-point interactions proposed by Sellier et al. [100]. It consists in approaching the zero-pressure geometry by iteratively updating the reference configuration, calculated by subtracting the nodal displacement vector between the updated deformed configuration and the target in vivo configuration until a required error tolerance is reached. Rivero et al. successfully applied a similar pullback algorithm to 12 patient-patient specific models of AAA built from CT scans which were assumed to be at uniform diastolic pressure on the image-based deformed geometry. They tested isotropic and anisotropic material models, assuming material homogeneity with reference material constants from the literature [238]. Rausch et al. proposed an augmented Sellier’s method based on Aitken’s delta-squared process, by introducing an augmentation parameter to accelerate the convergence rate and increase the chances of convergence. The method was applied to find the unloaded geometries of a thrombus and heart valve leaflets from animal models with geometries and properties collected from previous studies [239]. More recently, Das et al. proposed the shrink-and-fit algorithm, that assumes the unloaded configuration is a shrink analogous to the loaded reference geometry. On each iteration step, the coordinates of each node are mapped into a smaller geometry affected by a circumferential and axial shrink factor, the new geometry is loaded by the reference pressure until the least squared error of the nodal coordinates of the reference and inflated model is minimized. The method was then applied to resolve the unloaded configuration of an ideal and a patient-specific artery model assuming Mooney-Rivlin hyperelastic behavior and employing the Nelder–Mead optimization algorithm [240].

A different iterative approach is to solve the strain and stress distribution that balances the applied loads acting on the image-derived anatomic configurations without the resolution of the unloaded geometry [232]. Some methods that fit this category are the backward incremental algorithm and the modified updated Lagrangian formulation. In the backward incremental algorithm, small increments of the pressure load are applied to the reference geometry, the resulting stress state is mapped to the reference geometry as the initial condition for the next pressure increment until static equilibrium is reached. This method was applied by de Putter et al., using patient-specific AAA geometries and pressure loads to determine the stress distribution at diastole while assuming isotropic Neo-Hookean material behavior with population-averaged constants [241]. Similarly, the modified updated Lagrangian formulation applies consecutive small loads increments on the image-based reference configuration to build up an incremental multiplicative update of an independent deformation gradient. This method was used by Gee et al., to study the diastolic stress distribution of three patient-specific AAA geometries derived from CT scans with population-averaged pressure loads and material constants for an isotropic Neo–Hookean constitutive equation. In this work, the outcomes of the modified updated Lagrangian formulation are compared to direct solutions with inverse FEM, concluding that both methods yield similar diastolic stress distributions although the latter seemed more prone to solution multiplicity and buckling [242,243]. However, iterative methods may require suboptimal convergence times, and on some occasions, convergence could fail altogether [100].

It is important to highlight that even at an unloaded configuration, cardiovascular tissue is not truly stress-free. This fact has been widely proven by opening angle experiments at different arteries and layers of the heart wall. The residual stress responsible for this recoil effect exists without any distending pressure, being the possible result of non-uniform growth and remodeling over the patient’s entire lifespan. The latter implies that residual stress cannot be resolved solely from load-deformation data. Indeed, the most common technique for the estimation of residual strain relies on the quantification of the opening angle after a stress-relieving cut. Some specialized studies have collected opening angle data from multiple locations of the cardiovascular system through experimental tests on human cadaveric tissue. These experiments have shown that the opening angle, and thus the preexisting residual stress, depends on specific tissue location and individual factors such as age and health conditions. Consequently, generic or averaged opening-angle derived residual stress can hardly be used for patient-specific analyses, especially, in pathological cases. The constrained mixture theory provides a consistent framework for the estimation of residual stress through the modeling of growth and remodeling and could be the key, along with the image-based resolution of tissue composition, for a truly patient-specific estimation of a stress-free reference configuration [32,244].

### 6.2. The Heart

The relatively large thickness of cardiac tissue allowed the resolution of image-based kinematics even at the early stages of this technology. For this reason, along with the key role of the heart as the driving element of circulation, the heart was the first physiological system subject to patient-specific inverse analyses. Sermesant et al. and Aguado-Sierra et al. proposed comprehensive patient-specific models for cardiac function including the resolution of the unloaded configuration, bioelectrical activity, passive and active tissue properties, and hemodynamics [245,246]. These authors evaluated the possibility of solving such inverse problems with data acquired with medical imaging and electrocardiography and concluded that such comprehensive models easily became overparametrized, and computationally expensive to be solved by the available resources at the time. In consequence, most inverse models focus on only one or a few of their constituents instead of the whole heart. In the following subsections, we classify the research approaches based on the variables chosen to be solved by the inverse method.

#### 6.2.1. Properties of the Healthy and Infarcted Ventricular Wall

The ventricular wall is a complex multilayered composite responsible for delivering the driving force to pump blood throughout the cardiovascular system. The myocardium is the functional layer of the ventricular wall, containing the myofibers responsible for the active contraction of the muscle and the structural collagen fibers that contribute to its bulk mechanical properties. An accurate understanding of myocardial mechanics is key for the diagnosis and treatment of diverse cardiac pathologies, and potentially, predicts and stratifies the risk of heart failure after infarct. Therefore, many studies have focused on the estimation of mechanical properties of healthy myocardium, and more interestingly, estimating the effects of ischemia, and quantifying the properties of infarcted cardiac tissue to yield a truly patient-specific risk assessment of cardiac failure. Most developments relied on FEM for the solution of a forward problem (summarized in Table 7).

##### Homogeneous Models

The assumption of material homogeneity is a common and convenient simplification for forward and inverse models. It limits the number of parameters to be fit while still reproducing the overall mechanics of the organ with reasonable accuracy. Even though the myocardium is highly complex and spatially heterogeneous, homogeneous models may be deemed to be adequate for the study of healthy hearts, or when the aim of the analysis is not centered on the study of focalized lesions. In the study of tissues with steep localized changes in structure and properties, as in infarcted myocardium, the material homogeneous models cannot reproduce the localized strain and stiffness distributions on the infarct itself and the infarct borderzone, providing only averaged estimations of local deformation and material properties. However, these averaged properties can still be used as a measure of lesion severity by comparative studies of healthy cases.

One of the first simplified inverse models of the left ventricle (LV) was introduced by Hassabalah et al., to study the compressibility of the myocardium [257]. An idealized truncated ellipsoid matching MRI-derived averaged dimensions of a human LV was used as a computational domain. Fiber orientations were assumed helical with the linear transmural distribution established from DT-MRI data from Helm et al. [258]. The myocardium was modeled as a homogeneous fiber-reinforced Ogden hyperelastic material. The active tension of myofibers was assumed to be proportional to the pressure load, the latter being prescribed as a boundary condition at the endocardial surface. A uniform elastic foundation was applied to the pericardium to simulate the interaction of the heart with the surrounding organs, and all displacements were fixed on a lateral node. All material parameters were fixed except for the bulk modulus, which was optimized to fit a measured pressure-volume curve. The authors conceptually divided the cardiac cycle into the following consecutive stages: atrial systole, isovolumetric contraction, rapid ejection, isovolumetric relaxation, rapid filling, and reduced filling. This study suggested that the volume of the myocardium changed slightly during the cardiac cycle. According to this, myocardium behaves as an incompressible tissue only during rapid and reduced ejection and isovolumetric relaxation stages, while showing some degree of compressibility in the atrial systole, isovolumetric contraction, and filling stages. These observations are in agreement with in vivo compressibility measurements in large mammals [134].

**Figure 12.**
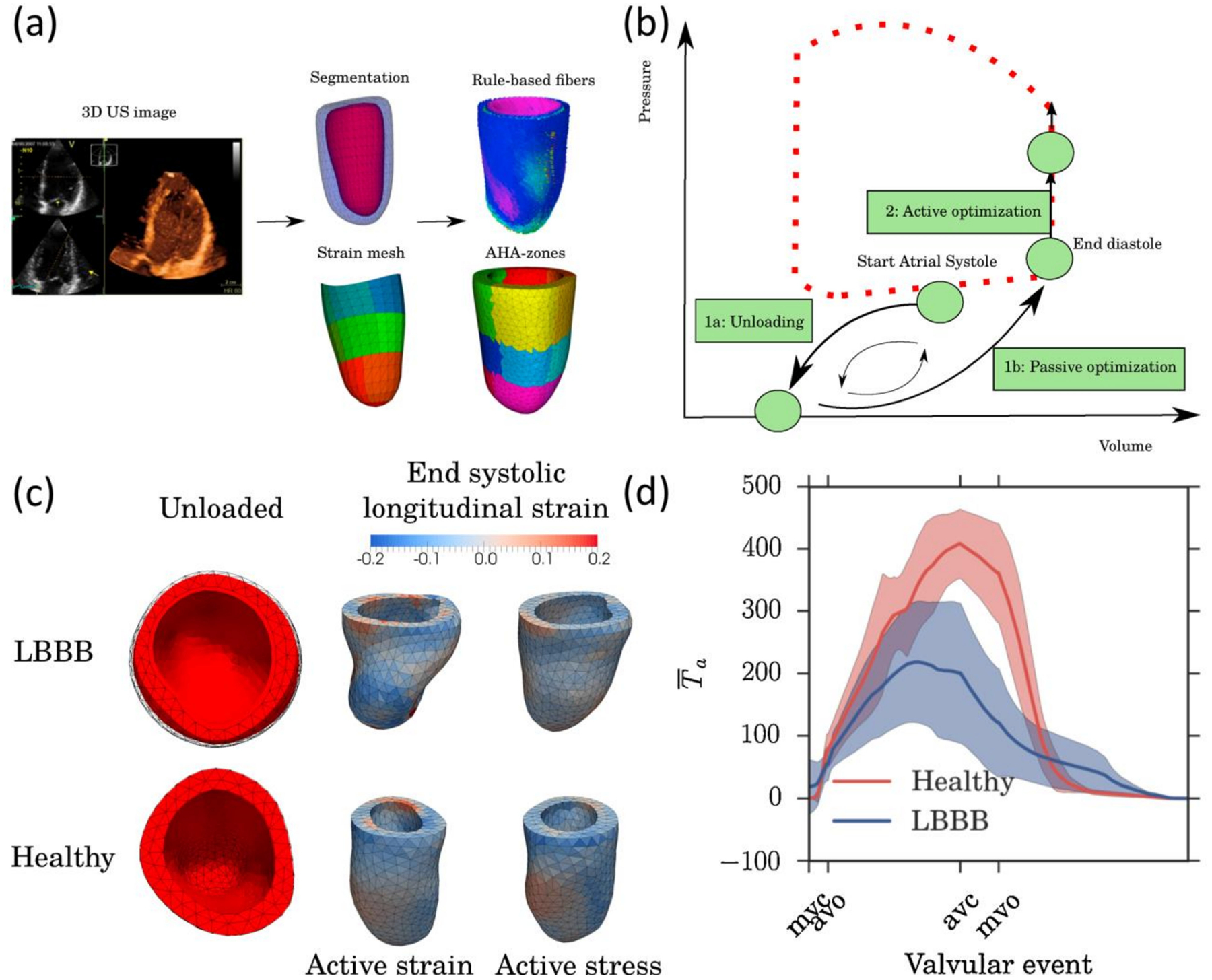
Inverse analysis of left ventricular mechanics. (Reprinted/adapted with permission from Ref. [235]. 2018, Elsevier). (**a**) Preprocessing pipeline, from left to right: medical image-based segmentation and kinematics, anatomic model generation, discretization, partition into 17 AHA standard regions. (**b**) Optimization loops, between unloaded and diastolic configuration for passive properties and between diastole and systole for active contraction parameters. (**c**) Resulting unloaded configuration and strain distributions for a healthy volunteer and an LBBB patient, using active stress and active strain approaches. (**d**) Comparison of activation parameters over time for a healthy individual and an LBBB patient showing the effect of impaired bioelectrical function. Acronyms: mvo, mitral valve opening; mvc, mitral valve closure; avo, aortic valve opening; avc, aortic valve closure.

A more sophisticated inverse analysis was presented by Xi et al., in two consecutive papers published in 2011, introducing patient-specific geometries [255,256]. MRI-based models of the LV at end-diastole were assumed as the zero-strain reference and discretized with Hermit-cubic finite elements. MRI tissue tagging was used to assess the diastole-to-systole displacement distribution and then interpolated into the nodes of the FEM mesh. The Fung–Guccione constitutive equation was selected to model the uniform passive properties of the myocardium. Myofiber orientations were assigned with a rule-based algorithm from Bayer et al. [55] based on canine serial histology from Usyk et al. [259]. Boundary conditions consisted of catheter measured ventricular pressure increments, zero traction at the epicardium, and apical and base displacement from tissue tagging. The least squared nodal displacement error was minimized using a reduced-order Kalman filter. The method was applied to one healthy heart and two patients with diastolic heart failure with impaired ejection fraction. The authors found a large difference in material parameters between healthy and heart failure patients, although the authors recognized the results were likely not unique for the given dataset. In addition, they found that passive behavior alone could not fully describe the deformation state at early diastole. They addressed this issue by introducing a time-dependent homogeneous active tension model and the backward displacement method to estimate the unloaded configuration [47]. Two different minimization problems are solved iteratively: first, the estimation of passive properties by consecutive simulation of deflation from early diastole to unloaded configuration followed by inflation to end-diastole; second the estimation of active properties by inflation from late diastole to systole (Figure 12b).

The target minimization function for both iterative loops was defined as the error in nodal coordinates between simulations and interpolated tissue-tagging measurements for their corresponding end of process configuration. According to their results, the residual activation state from early to end-diastole was larger for patients with heart failure, which may indicate that diastolic relaxation is impaired after cardiac failure due to the compensation mechanism to maintain cardiac function.

Genet et al. applied methods to define patient-specific anatomic models and define active and passive behavior similar to Xi et al. [47], although with no available patient-specific pressure data and the assumption of end-diastole as the stress-free reference [110]. Boundary conditions constrained all displacement on the basal plane of the ventricle and the dynamic boundary condition was a prescribed volume change, instead of the pressure increment. In the absence of pressure data, Genet et al. assumed a well-established normalized LV pressure-volume curve of Klotz et al. [109] as the optimization target. MRI tissue tagging measurements were used to validate the converged results, which showed good agreement with image-derived circumferential and axial strain. Regarding fiber stress distribution, the authors found that the end of diastole myofiber stress peaked near the subendocardial wall. They also highlighted that the transmural variation of the end-of-systole myofiber stress was nonmonotonic and was maximal at the mid-wall of the ventricle.

Solution multiplicity has been one of the main concerns about inverse methods, which motivated Nasopoulou et al. to explore how the definition of the optimization target functions can be designed to improve material property identifiability and solution uniqueness [252]. Sets of cine MRI and catheter pressure measurements were gathered from 7 cardiac resynchronization therapy (CRT) patients and one healthy volunteer. The configuration corresponding to the lower ventricular pressure was assumed to be stress-free. Patient-specific LV models were built at the reference configuration and a warping algorithm was used to estimate the displacement of the ventricular wall from cine MRI. The myocardium was assumed homogeneous and purely passive with a Fung–Guccione constitutive equation. Myofibers orientation was assumed to follow a linear transmural distribution following the findings of Streeter et al., on canine left ventricles [260]. Uniform pressure on the endocardium and image-derived displacements on the basal plane were imposed as boundary conditions. Two target functions were defined, one based on the displacement error, and the other defined as a normalized error of the pressure-energy input and stored strain energy. Two optimization processes were implemented consecutively to minimize the two error functions, which constrained the number of possible solutions. The authors concluded that a single purely geometric target function is unable to constrain the parameter space, while the application of the energy-based target function isolates one of the material parameters, that in conjunction with a geometry-based target provides a unique estimation of parameter sets.

Most of the inverse modeling approaches dealing with the heart, either constrain or prescribe measurement-derived magnitudes of displacements on the basal plane and/or the apex. Asner et al. highlighted the necessity of imposing more physiologically meaningful boundary conditions for the adequate assessment of cardiac mechanics. These authors proposed a method to impose consistent boundary conditions for ventricular mechanics based on non-invasive tests alone [250,251]. The proposed method was applied to synthetic datasets for validation generated in silico with idealized geometries and known material properties, motion, and loads. Then, the method was applied to patient-specific datasets from three healthy volunteers and three moderately dilated cardiomyopathy patients. Cine MRI, tissue tagging, and PC MRI were collected and used as imaging data either to set up the forward problem or as target data for minimization. End of diastole configuration was used to build anatomic models of the LV which were assumed to be at the zero-strain reference. Tissue tagging-derived displacements were interpolated to the FEM mesh to obtain a smooth displacement field and PC MRI was used to estimate the stroke volume. The myocardium wall was assumed to follow a reduced-order Holzapfel-Ogden constitutive equation with a time-dependent homogeneous active stress model. Myofiber orientations were assumed to follow a linear transmural distribution based on the work of Streeter et al., on canine ventricles [260]. Ventricular pressure–volume relation was assumed to follow the normalized Klotz LV pressure-volume curve, and the PC MRI-derived diastole-to-systole flow ratio was correlated to the pressure pulse amplitude. The authors proposed a data-based method for imposing boundary conditions through the use of Lagrange multipliers and the minimization of energy potentials. The endocardial boundary condition was defined in terms of volume change, while the basal plane and the epicardial node boundary conditions were defined in terms of a virtual force proportional to their correspondent displacements. A Shamanskii–Newton Raphson procedure was used to resolve the material properties and boundary condition multipliers. Parameter fitting was solved in two steps: first, the passive material parameters were solved by minimizing a displacement-based error function from tissue tagging data between early-and end-of-diastole; second, the active components were fitted by minimizing an error function defined as a weighted average of nodal displacement error and pressure-volume curve error. The authors highlight the potential bias introduced by hard displacement restrictions as boundary conditions, which prevents the reproduction of naturally occurring torsional modes of deformation. They also highlight that the direct imposition of noise MRI-derived displacement as a boundary condition can introduce computational issues associated with continuity and solution smoothness, concluding that the proposed weak formulation of boundary conditions is advantageous.

Palit et al. performed an inverse analysis of biventricular models with a microstructural material model [108]. Steady-state free precession (SSFP) cine MRI was used to build anatomical models, and to calculate the diastole-to-systole volume change of both ventricles from five healthy adult volunteers. A purely passive Holzapfel–Ogden material model was imposed with fiber orientation following the Laplace–Dirichlet rule-based algorithm by Bayer et al. [55]. Early diastole was assumed as the stress-free reference configuration. A normalized Klotz pressure–volume curve was set as the optimization target for a genetic algorithm. The authors introduced empiric constraints on the constitutive equation for the maximum absolute and relative values of shear-related terms to reduce the sampling space. In addition, they carried out a sensitivity analysis of the results on the assumed parameters. They concluded that variations within the normal ranges of interventricular pressure and fiber orientation did not produce significant changes in material parameters estimations.

Wang et al. explored potential differences in LV stiffness among heart failure patients with preserved and reduced ejection fractions [248]. Cine MRI and catheter pressure measurements were collected from 8, 11, and 5 individuals with reduced, preserved, and normal ejection fractions, respectively. Anatomical models of the LV were customized for all cine MRI images with an interactive guide-point modeling tool and volumes were matched to pressure measurements. The diastasis state (immediately after rapid filling) was assumed as the reference stress-free configuration. The Fung–Guccione model was used to describe myocardium mechanical behavior neglecting the active component. Myocardium fiber orientation was defined following the rule-based algorithm proposed by Nielsen et al., based on the fibrous structure of canine hearts [261]. Boundary conditions consisted of uniform pressure at the endocardium and constraints to the displacement of the basal plane. Image-based endocardial and pericardial surfaces were projected into finite element model predictions for each time step and the mean-squared error was minimized by fitting the passive material properties. Results showed no significant differences in ventricular stiffness between groups, although patients with reduced ejection fraction presented elevated diastolic stress levels.

Rumindo et al. explored the variability of in vivo estimations of passive and active properties of the LV in healthy individuals through inverse methods [247]. This retrospective study gathered cardiac MRI datasets from 21 volunteers with normal cardiac function. End systolic and diastolic volumes were calculated from MRI segmentation, and end-systolic configurations were assumed the stress-free reference and used for geometric modeling and meshing. The Fung–Guccione material equation and a time-dependent homogeneous active stress model were used to describe mechanical behavior. Myofiber orientation was assigned following the equations proposed by Rijcken et al., to optimize cardiac ejection [57]. Models were uniformly pressurized in the endocardium while constraining all displacements on the basal plane and assuming the epicardium to be traction-free. The Nelder–Mead algorithm was used to fit the material parameters by minimizing the least-squared error of the pressure–volume relation to the normalized Klotz curve. Population-based statistics were calculated showing that results were consistent within this population of similar characteristics. The authors highlighted the variety of reported Fung–Guccione material parameters from among different studies and discussed the relevance of the selection of a reference configuration for the estimation of passive properties.

##### Heterogeneous Models

Modeling of material heterogeneity can provide better fits to kinematic data, resolve property changes, and identify the location and severity of myocardial lesions. However, this comes with an increased modeling effort and computational expense. By assuming material heterogeneity on inverse methods, the number of parameters to be fitted increases, posing a burden on the optimization algorithm and complicating the solution of the forward problem. A common approach is to approximate spatial variations of myocardium properties and microstructure with region-wise heterogeneities. The simulation domain of the myocardium is divided into segments, each one with its own set of homogeneous material properties. The American Heart Association (AHA) proposed the division of the left ventricle into 17 standardized LV segments which have been adopted extensively in the study of myocardium mechanics (Figure 12a) [262].

One of the earliest inverse analyses of biventricular models with region-wise material heterogeneity was proposed by Marchesseau et al. [254]. The study gathered cine MRI datasets from 8 healthy volunteers and 3 heart failure patients with impaired ejection fractions. Cine MRI was used to estimate the volume change of both ventricles, to identify the location of the epicardial surface on several time-steps over the cardiac cycle, and to estimate displacements with a diffeomorphic free form deformation algorithm. End-diastole was used as the reference configuration and to build a deformable FEM mesh. Electromechanical behavior was modeled with a Bestel–Clément–Sorine model, which consists of a Mooney–Rivlin hyperelastic material matrix reinforced with fibers with passive elastic and time-dependent active components. Fiber orientation was assumed to follow the Laplace–Dirichlet rule-based algorithm by Bayer et al. [55]. The active component was assumed to have a viscous dissipation component and was modeled by a two-differential equation system solving for the time-dependent active stress and sarcomere stiffness as a function of an activation state variable. Parameter fitting is carried out by applying Kalman filters in two steps: first, a general fit is achieved with the overall pressure-volume curve, followed by a parameter refinement for each sector using sector-specific displacements and change of LV section volume. The model was able to locate the infarcted regions by assigning them lower contractility, while healthy patients converged to more homogeneous property distributions and normal active function.

In 2017, Gao et al. performed an inverse analysis on 27 healthy subjects and 11 patients with acute myocardial infarction [253]. Gadolinium-enhanced and cine MRI were applied to identify the location of infarcted regions and to calculate the volume change of the LV. Anatomic models were built at end-diastole, which was assumed as the zero-strain reference. The LV systolic blood pressure was approximated by the sphygmomanometer systolic measurements. The anatomic models were divided into the 17 standard AHA regions, and circumferential strains were calculated for each region through a b-spline deformable registration algorithm. Non-infarcted tissue was modeled as a Holzapfel–Ogden material with a sophisticated differential-algebraic model for active stress. Myofiber orientation was defined by the minimum-distance rule-based algorithm Potse et al. [56]. Infarcted tissue was assumed 50-fold stiffer than regular tissue with no active contraction. A Bayesian approach with Gaussian processes and automatic relevance determination algorithm was used to fit material properties and active contraction parameters by minimizing a weighted function of the volume error and region-wise circumferential strain error. Results showed that active tension was larger in infarcted hearts, which agrees with the early observations of Xi et al. and Marchensseau et al., for which the authors hypothesized the existence of a compensation mechanism for infarcted hearts to preserve stroke volume.

In 2018, Finsberg et al. compared the LV contraction between healthy adult volunteers and patients with blocked or delayed electrical activation impulses, a condition called left bundle branch block (LBBB) [235]. The study was carried out on a population of 7 individuals per group. Four-dimensional (4D) echocardiography was used to build patient-specific anatomic models and FEM meshes. Ventricular volume was measured at 10 different instances within the cardiac cycle. Ultrasound speckle tracking was used to estimate the piece-wise strain field, consisting of circumferential, radial, and longitudinal strain at each of the 17 standard regions. Direct pressure measurements were obtained through catheterization for the LBBB patients. The myocardium was assumed to follow a uniform Holzapfel–Ogden material model, while two models for active contraction (active strain and active stress) were tested. Myofiber orientation was assigned following the Laplace–Dirichlet rule-based algorithm by Bayer et al. [55]. Rigid-body translation and rotation were constrained by an elastic foundation boundary condition on the basal plane imposed as a collection of linear springs with uniform elastic constants. Two iterative inverse models were solved consecutively in each case: first, the passive isotropic material properties and unloaded configuration were estimated with a backward displacement algorithm using the geometric and pressure information at early and late diastole. Second, the active and anisotropic material properties were obtained by minimizing an error function defined as a weighted average of ventricular volume and strain error (Figure 12b). Minimizations were carried out with a sequential quadratic programming algorithm, and maximum value constraints were imposed on active model parameters. Results suggested that the myocardium wall was more compliant for the healthy group (Figure 12c) and that active contraction was significantly lower for the LBBB, which is consistent with an impaired propagation of the activation pulse (Figure 12d). Both the active stress and active strain models showed equivalent results. A similar methodology was later applied to 12 patients with pulmonary hypertension and 6 healthy human controls, using cine MRI and hyperelastic warping to estimate regional strains [249]. This study found that larger right ventricular contractility affected the right-to-left ventricle volume ratio, the latter being a clinical risk factor for pulmonary hypertension. The authors suggest that this mechanistic relation between ventricular contractility and interventricular volume ratio could provide further insights into pulmonary artery hypertension risk stratification.

Zhang et al. studied the local effect of ischemia with the segment-wise heterogeneity approach [17]. Five patients with functional mitral regurgitation associated with coronary artery disease and treated with percutaneous revascularization were retrospectively recruited. The population was complemented by one healthy volunteer. The treatment protocol included cardiac MRI and transthoracic echocardiography before and 3 months after revascularization. Gadolinium-enhanced MRI allowed the identification of infarcted scar tissue and an MRI stress perfusion test was used to assess the location and severity of ischemia. With this image-derived information, a normalized scale for infract and ischemia severity was assigned to each region. MRI-derived patient-specific 3D biventricular models at early diastole were used to define the geometrical model and assigned to be the zero-stress reference. MRI tissue tagging was used to estimate average strains in all 17 standard regions. Left and right ventricular pressure were estimated from sphygmomanometry and concomitant transthoracic echocardiography, respectively. The Fung–Guccione material model was used to describe passive behavior and a time-dependent heterogeneous-by-region active stress model was implemented. Myofiber orientation was prescribed following the Laplace–Dirichlet rule-based algorithm by Bayer et al. [55]. Measured left ventricular pressure was applied to the endocardium of the stress-free early diastolic model while constraining axial displacements on the basal plane. Boundary loads consisted of right ventricular pressure at the septum and a traction-free condition at the epicardium. Passive and active material parameters were defined in terms of a scale of the infarction and ischemia severity. This ischemia effect factor modulated different responses with regions identified with zero severity behaving like healthy tissue and becoming stiffer and less actively contractile with larger lesion severity. Material parameters and the ischemia effect factor were fitted for each one of the 17 regions by minimizing a weighted function of the mean square error of diastole-to-systole volume change and the region-wise average strain. Results agree with previous studies on predicting the stiffening of regions corresponding to infarcted tissue and border zone. Additionally, the model allowed the estimation of the ischemia effect on tissue stiffening and the recovery of compliance after revascularization treatment.

One of the main limitations of the above studies is the assumption of either spatial material homogeneity or segment-wise heterogeneity, however, material properties are likely to vary continuously throughout the myocardium. To address this, Balaban et al. proposed an iterative inverse method to resolve the heterogeneous distribution of mechanical properties on an LV model from a 64-year-old heart with systolic heart failure, LBBB, coronary artery disease, and chronic infarction in the inferior section of the LV [141]. 4D echocardiography was used to obtain the anatomic model and FEM mesh at early atrial systole. Speckle tracking was used to estimate systolic strain averaged over the 17 standard regions, and pressure was measured by catheterization. Gadolinium-enhanced MRI was used to identify the location of infarcted fibrotic tissue. The Holzapfel-Ogden material model was implemented allowing spatial variations of the scalar material parameters with a piece-wise linear representation with fiber orientation following the rule-based algorithm proposed by Bayer et al. [55]. Active tension was neglected, and end-diastole configuration was assumed stress-free. Rigid body motion was constrained by impeding axial displacement at the basal plane and apex, and by an in-plane elastic foundation at the base plane imposed as a collection of linear springs with uniform elastic constant. A sequential quadratic programming algorithm was applied to estimate the almost 3000 spatially distributed material parameters. To favor convergence to smooth distributions, optimization was constrained by a first-order Tikhonov functional. Results show that estimated strains were lower, and the material stiffer, in regions corresponding to infarcted tissue and its immediate surroundings identified by gadolinium-enhanced MRI.

### 6.3. Valves and Leaflets

Each one of the chambers of the heart is equipped with a discharge valve to ensure unidirectional blood flow, acting mostly passively to changes in transvalvular pressures. The atrioventricular valves are the mitral and tricuspid, for the left and right sides of the heart respectively. These valves typically define the basal plane and separate the atria from the ventricles (Figure 10a). They are structurally supported by the papillary muscles and chordae tendineae to hold the valves closed during systole and avoid ventricle-to-atria backward flow. The pulmonary and aortic valves regulate blood flow from the ventricles to their homonym arteries and are not supported by any subvalvular apparatus. The main element of heart valves are fibrous structures called leaflets or cusps, that flap to allow or impede blood flow. In normal conditions, only the mitral valve has two leaflets while the other valves have three [263].

Heart valve disease is mostly related to regurgitation, stenosis, and atresia. The former consists of backflow due to deficient closing, stenosis is the hardening and thickening of the leaflets, preventing the valve to open properly and result in increased load in the heart, while the latter is a congenital disease where the heart valve is partially or completely absent. Heart valve malfunction can lead to several complications such as heart failure, blood clotting, stroke, and death. Heart valve disease is most common on the left side, as the aortic and mitral valves are loaded with larger pressures, and in consequence, they have received more attention from the medical and scientific community. However, attention to right heart valves has significantly grown in the last two decades along with the awareness of pulmonary artery diseases [264].

There is a considerable body of research on the forward modeling of heart valve function accounting for structural and FSI mechanics, usually validated against in vitro experiments [265]. However, leaflets are typically thin structures (<1.5 mm) showing complex displacement patterns, which renders them extremely challenging to resolve through in vivo imaging techniques. Owing to this, most inverse analyses of valve mechanics are based on in vitro experiments on excised or synthetic valves, where the leaflet displacement is resolved with the use of physical markers [266–273], or with high-resolution cameras [147,274].

In vivo inverse modeling of ovine heart valves function has been achieved by the use of fluoroscopic markers implanted on the surface of mitral valve leaflets [275,276], a technique that cannot be pursued in human studies. More recently, Lee et al. applied ultrasound technology to assess the anatomy and displacement of the mitral valve of ovine animal models to explore the use of inverse modeling, and in vivo mechanical properties and stress distribution were successfully estimated [271,277].

Aggarwal et al. estimated the residual strain on human aortic valves by combining in vivo imaging with measurements on explanted tissues [278]. The authors collected in vivo transesophageal 3D echocardiographic images of the aortic valve from five open-heart transplant patients at three configurations: fully open, just-coapted, and fully loaded. Each aortic valve leaflet was excised during surgery and then imaged in a flattened configuration ex vivo. Strains were calculated between the ex vivo stress-free configuration and the three in vivo configurations from echocardiography segmentation by the application of a spline parametrization algorithm. Results suggest that leaflets are significantly pre-strained with respect to the excised reference even at the just-coapted configuration where the transvalvular pressure load is negligible. Results also showed that leaflet deformation is larger in the radial direction if compared to the circumferential direction, the latter being structurally stiffer due to the alignment of collagen fibers.

The work of Aly et al. stands out as one of the few in vivo works on human heart valves for the generation of transient anatomical models [279]. In this work, 4D ultrasound was collected from 28 patients, half with normal mitral valve anatomy and function, the other half with ischemic mitral valve regurgitation. An automatic inverse algorithm uses the manual identification of five key landmarks on the leaflet anatomy as input. Then, Kalman filter optimization is used to build anatomical models at different instants of the cardiac cycle. According to the authors, this algorithm could be used as the base for more comprehensive inverse modeling to assess leaflet material properties.

### 6.4. Arterial Wall

Changes in mechanical properties of arterial walls have been associated with the onset of multiple cardiovascular pathologies (e.g., atherosclerosis, dissection, stenosis) and remains an important predictor of cardiovascular morbidity and mortality in clinical practice. This motivated the development of early techniques for the non-invasive assessment of arterial stiffness through the evaluation of luminal area change and pulse wave velocity. These techniques, although useful, can only provide a gross estimation of material properties as they introduce many assumptions and simplifications related to homogeneity, perivascular support, and linearized behavior.

The image-based resolution of vascular tissue kinematics is technically challenging; the main reason being the relative thinness of vascular walls. For example, the ascending aorta has a typical thickness of about 2.5 mm, which decreases to about 1.5 mm at the abdominal aorta, and the pulmonary artery is only about 0.2 mm thick. These length scales are comparable to the highest resolutions available on imaging techniques, for which luminal area changes (either with or without contrast agents) remained the main input for early inverse analyses of arteries. However, recent developments in ultrasound speckle tracking and DENSE MRI techniques make available arterial wall displacement measurements on a meaningful number of pixels. Most approaches rely on FEM for the solution of the forward problem (summarized in Table 8).

One of the earliest works on inverse arterial mechanics was introduced by Taviani et al., in 2008 [288]. Cine MRI was used to assess the cross-sectional geometry and distension of the common carotid artery of three healthy volunteers, while applanation tonometry was utilized to gather pressure wave data. The wall was assumed to behave as a nearly incompressible linear-elastic isotropic material with the diastolic configuration as the unloaded stress-free reference. The luminal surface was loaded with the measured pressure increment, while the adventitial surface was assumed traction-free. An optimization algorithm iterated over the elastic modulus while minimizing the normalized distance between the simulated and measured lumen. The method was successfully validated with a silicon rubber phantom and provided consistent results among all healthy adult volunteers. This inverse model of the common carotid artery was improved by Franquet et al., who incorporated the effect of perivascular support by attaching the adventitial surface to a homogeneous compressible-elastic boundary with fixed properties and a third embedded body representing the superior vena cava [114]. A Levenberg–Marquardt optimization algorithm was used to minimize a shape-based error function that accounted for pixel-wise signal intensity to define the location of the lumen. Additionally, the authors studied the effect of variability on the luminal area and wall thickness estimations used to define the reference configuration. The method was again validated against a silicon rubber phantom and applied to two adult healthy volunteers showing good agreement with estimations of elastic moduli reported in classical literature.

To incorporate the effect of residual and pre-stresses on the loaded diastolic configuration, and to fit a more complex material model, Masson et al., proposed a semi-analytical approach [289]. Clinical data from two adult volunteers 33 and 64 years of age consisted of 2D ultrasound on the common carotid artery, which was used for the resolution of luminal area change and the thickness of the intima-media layers. Additionally, planar tonometry was used to estimate the pressure wave. The carotid artery was assumed to be a pre-stressed bi-layered idealized straight cylinder. The passive material properties were assumed to follow an incompressible four-fiber family elastic constitutive equation, and active tension was assumed to act on the circumferential direction according to a single-equation active stress model. Perivascular support was modeled as a uniform adventitial pressure that exponentially increases with area increments. The forward problem was formulated as the solution of the luminal pressure corresponding to area changes assuming purely radial displacements. A Levenberg–Marquardt optimization algorithm was used to minimize the least-squared error of the predicted and measured pressure waveform. The optimization algorithm fitted 14 parameters including pre-stress parameters (opening angle and axial pre-stretch), material parameters for the two material layers, and active tension constants. The method successfully reproduced the pressure waveform while adjusting the material parameters. The authors reported that prestretch and active stress constants were similar among both patients, but passive material parameters reflected stiffer material for the older subject.

One of the first uses of image-based kinematics to estimate the anisotropic mechanical properties of a realistic large artery model was introduced by Wittek et al., in 2013 [115]. 4D ultrasound records with speckle tracking of the abdominal aorta were retrospectively collected from five healthy adult volunteers in segments proximal to the *truncus coeliacus*. Diastolic and systolic pressures were measured at the brachial artery with a sphygmomanometer. Diastolic 3D models of about 50 mm in length were segmented from ultrasound images assuming a fixed wall thickness of 1.6 mm. This configuration was assumed to be axially pre-strained by a quantity estimated by an empirical correlation. The arterial wall was assumed to behave as a modified Holzapfel–Ogden material. Perivascular support was modeled as a uniform adventitial pressure of 20 mmHg. A Nelder–Mead optimization algorithm was applied to iterate over the parameters of the material model to minimize the error of Biot’s strain tensor between the benchmark measurement-derived model and the simulation. Each iteration consisted of the solution of three sequential problems: first, the inverse solution of the unloaded configuration for the given diastolic pressure and axial prestrain through a backward displacement algorithm; second, the stretch from diastolic to systolic configuration by the imposition of measurement-derived displacements to produce the benchmark model; and finally, the inflation from diastolic to systolic geometry through incrementing luminal pressure for the simulation. The resulting material parameters were used to produce stress–strain plots, which showed reasonable agreement with experimental biaxial test data from excised tissue. This method was further refined in 2016 by improving the error function and optimization algorithm. The error function was based solely on image-based estimations of strain instead of the benchmark model output. The deterministic Nelder–Mead algorithm was complemented with a stochastic Monte Carlo algorithm for the iterative generation of parameters to avoid convergence to local minima [125]. The improved method was applied to three clinical ultrasound datasets from a healthy adult volunteer, a patient with peripheral arterial occlusion, and an AAA patient. Results predicted stiffer material behavior of the arterial wall for diseased individuals when compared to results on healthy volunteers.

Pourmodheji et al. collected cine and PC MRI images, and intracardiac catheterization pressures from a pediatric patient with pulmonary hypertension and a cardiac transplant control subject. A 3D model of the main pulmonary artery with its proximal left and right branches was created at the diastolic configuration. The material model was assumed as a homogeneous constrained mixture of elastin fibers, four families of collagen fibers, and an incompressible continuum of smooth muscle cells. The constrained mixture theory was applied to prescribe pre-stretches to each constituent to balance the diastolic pressure load. An L-BFGS optimization algorithm was applied to iterate over the material parameters to minimize the cumulative error to the measured pressure–area curve at the main pulmonary artery. The model suggests that pulmonary hypertension-induced remodeling led to the stiffening of elastin fibers and wall thickening.

All of the above models assume that arteries are uniformly loaded at the luminal and adventitial surfaces. In the lumen, arterial tissue is subjected to blood pressure; however, loads and reactions on the adventitial surface are typically complex. Without appropriate adventitial boundary conditions, the deformation of a pressurized blood vessel at systole results in a homogeneously deformed configuration following the principle of minimal strain energy [14,115]. However, different image-based in vivo analyses have shown that large vessels may undergo heterogeneous deformations from diastole to systole, an effect that is not reproduced on standard in vitro pressurization setups or in silico experiments without appropriate adventitial boundary conditions [13,115,290].

These observations supported the hypothesis that the interaction of healthy blood vessels with diverse perivascular structures may induce the in vivo deformational heterogeneity [14,166]. To address this, Bracamonte et al. proposed a heterogeneous elastic foundation approach, consisting of the attachment of static linear springs of heterogeneous stiffness to the adventitial surface of arterial models. The distribution of stiffness of the elastic boundary was discretized to piece-wise constant regions and fitted through an iterative inverse algorithm to reproduce the heterogeneous deformation of the vessel [14]. For this study, retrospective cine and 2D DENSE MRI data were collected at the infrarenal abdominal aorta from nine healthy adult volunteers of diverse ages. DENSE MRI data were processed to obtain the spatial distribution of the diastole-to-systole displacement and then interpolated onto a FEM mesh built from the segmented diastolic configuration. The material was assumed to follow the Fung material model at a plane-strain state with the diastolic configuration as the unloaded stress-free reference. The Powell optimization algorithm was employed to iterate over the material parameters and elastic boundary stiffness distribution to minimize the least-squared error of the nodal displacement. Estimated material parameters reproduced the stiffening effect of aging. The elastic boundary stiffness distribution was independent of discretization and consistent among patients. Notably, it showed good agreement with the location of known anatomical features of the perivascular space, such that the vicinity to the vertebrae corresponded to the stiffest boundary, whereas the region adjacent to the peritoneal cavity resulted in the most compliant boundary.

**Figure 13.**
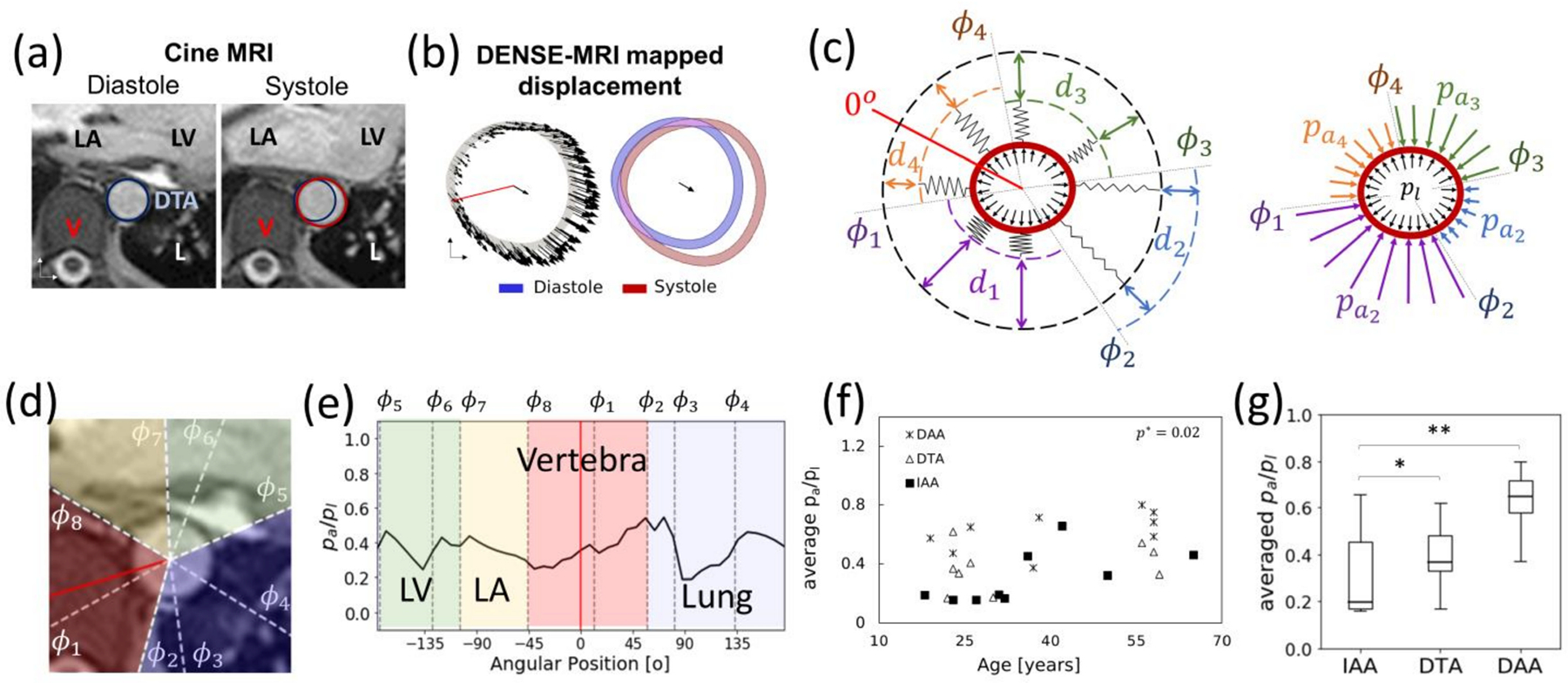
Inverse analysis of perivascular interactions at the descending thoracic aorta (DTA), based on results from Bracamonte et al., 2021 [150]. (**a**) Surroundings of the DTA. (**b**) DENSE MRI-derived displacement quiver representation and mapping into deformed (systolic) configuration. (**c**) Moving elastic foundation implementation and equivalent adventitial load distribution. (**d**) Patient-specific elastic boundary, and (**e**) adventitial load distribution. (**f**) Adventitial load increments with age. (**g**) Average adventitial load at different aortic locations (* *p*-value < 0.05; ** *p*-value < 0.01). Symbols: *ϕ* elastic boundary region angular delimiter, *d* moving elastic boundary displacement, *p*_*a*_ adventitial force per unit area, *p*_*l*_ luminal pressure increment, LA left atrium, LV left ventricle, V vertebra, L lung, IAA Infrarenal abdominal aorta, DTA descending thoracic aorta, DAA distal aortic arch. Red line (0°) is the angular reference selected as the closest location of the vertebra to the aortic wall.

The authors found that this approach properly captured the mechanics of the infrarenal aorta but failed to reproduce displacement measurements of the descending thoracic aorta, where the aortic wall shows both distention due to pressurization and bulk motion (Figure 13a,b). This bulk motion was hypothesized to be driven by the interactions with the adjacent beating heart. These interactions were modeled by incorporating a moving elastic foundation boundary approach [280]. This was implemented by attaching linear springs of homogeneous effective stiffness to the adventitial surface of the 2D aortic model, which was then allowed to displace radially (either inwards or outwards) to best reproduce the target bulk motion and heterogeneous wall deformation upon luminal pressurization (Figure 13c). The method was applied to a collection of retrospective cine and 2D DENSE MRI data at the infrarenal abdominal aorta, descending thoracic aorta, and descending aortic arch from 27 healthy adult volunteers of diverse, ranging from 19 to 65 years of age. A similar optimization algorithm was applied, although in this new model, the fitted elastic boundary parameters were the material model constants and spring displacement distribution, which translated directly to adventitial load distribution (Figure 13c). A parametric study was performed to study the effect of the moving elastic boundary parameters on the resulting estimations of distributed adventitial loads, which revealed that averaged adventitial load and adventitial load distributions were seemingly independent of elastic boundary parameters within the range that yielded physiologically meaningful results [280]. The proposed method converged to elastic regions that were located around relevant anatomical features (Figure 13d), and peak loads were found at locations where the heart pushes the aorta against the vertebrae (Figure 13e). Results suggest that adventitial load increases with age (Figure 13f), and that the thoracic aorta carries a larger adventitial surface load than the abdominal aorta, most likely due to the interactions with the beating heart (Figure 13g) [150].

#### 6.4.1. Aneurysms

Aneurysms are enlarged blood vessels caused by the remodeling of its wall. When local wall stress exceeds wall strength, rupture occurs, which carries significant morbidity and mortality. Brain and aortic aneurysms are the common manifestations of this disease. Aortic aneurysms have an incidence of 5 to 10 cases per 100,000 and are responsible for approximately 15,000 deaths per year just in the United States [291,292]. Maximum aneurysm diameter and expansion rate are currently the main criteria for diagnostics and risk assessment [293]. Notably, though rupture risk increases with a maximum diameter on average for the entire population, diameter alone struggles to predict rupture for any given individual. Thus, further research is ongoing to develop more reliable metrics for predicting rupture based on biomechanics [13].

**Figure 14.**
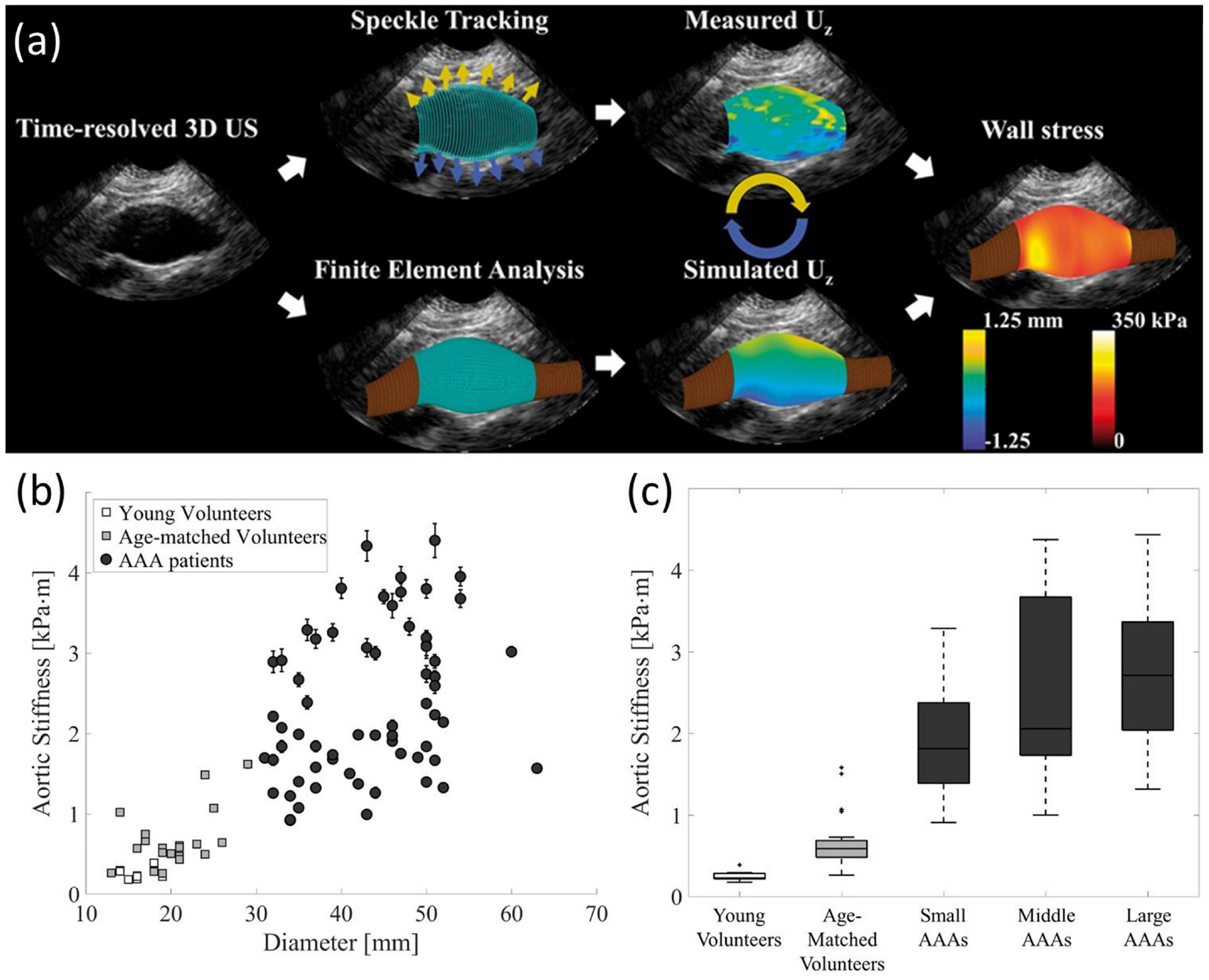
Inverse analysis of abdominal aneurysm mechanics (Reprinted/adapted with permission from Ref. [283]. 2018, Oxford University Press). (**a**) Patient-specific data processing algorithm and typical outputs. (**b**) Aortic stiffness versus maximum aortic diameter for healthy volunteers (gray squares) and AAA patients (black circles). (**c**) Population-based statistics of aortic stiffness in a box and whisker plot with dots representing outliers. Results suggest that most wall stiffening occurs at early stages of the disease when the aneurysm diameter is still relatively small.

For example, Karatolios et al. applied the inverse modeling approach of Wittek et al., (2013) to study the strain distribution in two abdominal aortic aneurysms (AAA) of two adults and the abdominal aorta of six healthy controls [164]. Results suggested that peak strains in AAAs are time-delayed (in late systole) with respect to their occurrence in healthy aortas. This work was followed by an extensive retrospective study published by van Disseldorp et al., in 2016, which gathered information from 40 AAAs patients that underwent CT scans and 4D Ultrasound with speckle tracking [282]. Patient-specific 3D models of the abdominal aneurysm were generated from the CT scans with a fixed wall thickness of 2 mm for all cases. The arterial wall was assumed to behave as a neo-Hookean material. The shear modulus was estimated iteratively to minimize the diastole-to-systole nodal displacement between forward FEM simulations and speckle-tracking derived measurements (Figure 14a). The error function was designed ad hoc so that regions with more precise and reliable measurements carried more weight when calculating error. For each iteration, the pressurization from diastole-to-systole was preceded by the estimation of the diastolic stress applying the backward increment algorithm. Systolic pressure was assumed to be 140 mmHg for all cases and the reference geometry built from CT scans was assumed to be at a mean arterial pressure of 105 mmHg. Interestingly, results from the study suggested that aneurysms with larger diameters tend to be stiffer. An extension of this work was published in 2019 by van Disseldorp et al., with a comparative study of material properties from 30 healthy volunteers and 65 AAA patients using 4D Ultrasound datasets [283]. Healthy cases were grouped by age, whereas AAA patients were grouped by aneurysm diameter. Segmentation and parameter estimation followed the same methodology as previously; however, patient-specific diastolic and systolic pressures were measured from a sphygmomanometer and used as boundary conditions for the backward increment method and forward simulations. The analysis showed a significant difference in stiffness between age-matched healthy volunteers and AAA patients even at the early stages of the disease (Figure 14b). The study suggests that most of the stiffening occurred at the onset of the disease with slight further increases as the aneurysm grows (Figure 14c). Additionally, a significant correlation between peak stress and aneurysm size was found, which is consistent with the general correlation of aneurysm size with wall rupture.

Krishnan et al., performed inverse model analyses on ascending thoracic aorta aneurysms (aTAA) [286]. These authors collected CT angiography and DENSE MRI sequences from four patients. Three-dimensional models of aortic aneurysms were built from the CT scans at systolic configuration. The Ogden isotropic hyperelastic constitutive equation was selected as the material model. They applied an iterative updating algorithm to find the set of material parameters that minimized the least-square error of simulated strains against DENSE MRI-derived estimations. The iterative algorithm consisted of three steps: first, a deflation step to 0 mm Hg (assume to be the zero-stress reference), followed by the inflation to the assumed 120 mm Hg at systole, and finally, the deflation to diastolic pressure of 80 mm Hg. This study revealed that the estimated peak principal stress is circumferential and about 25% greater than the average stress in aTAAs and located in the inner and outer curvature of the arch towards the pulmonary artery.

Liu et al. explored new methods to reduce the computational cost of inverse analyses while studying the mechanical properties of aTAAs. First, they investigated a method based on the computation of wall stress by solving a simplified statically determinate problem to obtain an “almost true” stress field [104]. They collected retrospective CT angiography from 4 patients with aTAA who went through surgical repair with tissue excision used for ex vivo biaxial testing. The geometry was built at the systolic configuration and assumed to be loaded at 120 mmHg. The material was modeled with a Holzapfel-Ogden constitutive equation. The backward displacement algorithm was used on each iteration to calculate the unloaded configuration assumed to be stress-free. An iterative inverse method was applied to obtain an estimation of material parameters using a constrained gradient-free trust-region optimization algorithm. Each iteration consisted of two steps: first, computing an almost true stress field from the in vivo geometries and loading conditions by using the Laplace law for statically determinate stiff thin-wall vessels; and second, calculating the stress distribution with the updated material parameters. The target function for the optimization algorithm was defined as the least-squared error of the simulated to almost true stress. Constraints consisted of upper and lower limits for material parameters extracted from the literature. Estimated material properties showed good agreement with results from patient-specific mechanical tests from excised tissue while decreasing the computational cost relative to regular iterative inverse approaches. Subsequently, Liu et al. used the same database and material model to explore the effectiveness of the multi-resolution direct search method as the optimization algorithm [113]. This algorithm works by decomposing the search for the optimal material parameters with a multi-scale representation of the parameter hyperspace. The target function to be minimized was defined in terms of the distance between surface nodes and the location of the segmented surface at systole. The converged material properties successfully reproduced the strain energy curves from biaxial testing while considerably reducing the computational cost of the inverse approach.

All these studies assumed material homogeneity of the aortic wall, which is a major limitation for the study of aneurysms. In vitro mechanical tests and histology analyses have been performed on aneurysms from human cadavers revealing both structural and mechanical heterogeneity [294,295]. Farzaneh et al. studied material heterogeneity on three aTAA patients from which CT scans were collected [112]. Medical images were used to build 3D models of the aneurysms at diastole and systole, and these models were used to estimate the local strain state. Each element on the wall surface was assumed to be part of an ellipsoid sharing the center to the cross-section of the vessel and was assumed to behave as a linearly elastic material. The stiffness was directly calculated element-wise from local balance equations. Their results suggested that diseased tissue was stiffer in the bulging part of the aneurysm and generally stiffer than the adjacent non-aneurystic tissue. Giuseppe et al. further applied this methodology to a cohort of 30 aTAA patients, 12 with bicuspid aortic valves, and the remaining with normal tricuspid valves [281]. Wall stiffness distribution was heterogeneous for each individual, however, regional differences appeared to be marginal within the cohort due to interindividual variability. Notably, this study found no significant differences in stiffness nor its distribution between the bicuspid and tricuspid valve groups, suggesting that no distinction should be made in the surgical management of aneurysms between these groups.

#### 6.4.2. Atherosclerotic Plaques

Atherosclerosis is a chronic inflammatory disease that manifests as the hardening and occlusion of arteries due to the build-up of plaque on the lumen of the arterial wall. Atherosclerotic plaque is a mixture of fatty substances, cholesterol, calcium, and cellular waste, usually enclosed in a fibrous cap. Atherosclerotic lesions are generated at specific regions of the arterial tree, mostly in the vicinity of branch points, the outer wall of bifurcations, and the inner wall of curves [157]. Among many possible associated complications, plaque can break and detach, generating thrombosis, acute myocardial infarction, and stroke. Thus, the in vivo evaluation of the mechanical properties of atherosclerotic plaques and their mechanical environment could support the assessment of risk associated with plaque rupture. One of the earliest inverse analyses of atherosclerotic plaques was proposed by Liu et al., in 2012 [286]. This study was performed on 12 patients with carotid artery atherosclerosis. For each patient, a set of cine MRI, 3D multi-contrast MRI, and sphygmomanometry were collected. Two-dimensional models of the diseased sections were built from MRI images at diastole, including lipid pools resolved by multi-contrast MRI. The arterial wall and plaque were assumed uniform and to behave as a Mooney–Rivlin hyperelastic material, while the lipid pools were assumed to be isotropic linear elastic. An L-BFGS-B optimization method was applied to fit the material properties of the wall plaque until the error between the simulated and measured diastole-to-systole area change was minimized. Each iteration included the estimation of the unloaded configuration by the shrink-and-fit algorithm, and a forward FEM problem for the inflation from the unloaded configuration to the systolic configuration applying uniform luminal pressurization. The authors found the estimations of material stiffness show reasonable agreement with reported data from experimental studies. An analysis of stress distribution indicated that, for all cases, peak stress was located at the thin cap covering the lipid core. This study was further refined by Wang et al., in 2017 [285], with similar imaging and functional data acquired for 8 patients with carotid atherosclerosis with follow-up tests after 18 months. The material models, optimization algorithm, target function, and iteration steps were the same as previously; however, a total of eight slices were analyzed from each carotid artery and modeled as a 3D thin layer so that axial prestretch could be included in the estimation of the unloaded configuration. Results revealed high patient-to-patient variability on plaque stiffness, which was significantly larger in the hypertensive cases. The authors also found that estimations of material properties of the plaque can significantly change over time, with stiffness increments being the most common scenario. Huang et al. further explored these results with FSI simulations based on patient-specific estimations of atherosclerotic tissues with patient-specific measurements of pressure gradients by applanation tonometry and confirmed that flow and pressure-induced stresses peak at the fibrous cap that covers the lipid core, which could offer support to explain the main mechanisms of plaque rupture [296].

The main limitation of previous studies is that the current resolution of non-invasive imaging techniques is insufficient to resolve the displacement of atheroma plaques in small vessels such as the carotid artery. To overcome this, Maso Talou et al. utilized intravascular ultrasound technology [284]. This work analyzed data from 4 atherosclerotic lesions which were modeled as 3D thin cross-sectional slices. Each model was single-layered and divided into six circumferential sections, each portion being assumed materially homogeneous and following the Neo–Hookean hyperelastic material model. Perivascular tethering was modeled as a homogeneous elastic media of fixed stiffness. Kalman filters were used to estimate material parameters for each section while minimizing the diastolic-to-systolic displacements. Each iteration included the estimation of diastolic stress distribution by a backward increment method assuming a pressure load of 80 mmHg and population average-based axial stretch. From this preloaded state, a forward inflation problem to systolic pressure was then solved. Parallelization techniques were employed to reduce computer processing times achieving convergence between 12 h and three days. Sensitivity of the results to numerical and model parameters was carried out, finding that perivascular elastic properties have a significant effect on material parameter predictions. The estimated material parameters agreed with the magnitudes reported from available experimental data.

### 6.5. Hemodynamics

In general, computational modeling of hemodynamics is more resource-consuming than tissue mechanics, as simulations need to account for transient effects and deal with the difficulties introduced by the non-linearities of convection and momentum dissipation. This makes the application of inverse modeling to hemodynamics a challenging task.

The use of simplified 0D (lumped) and 1D models can significantly reduce the computational cost. These simplified models have been used on a patient-specific basis and implemented onto inverse modeling approaches to provide useful systemic information about flow distribution, vascular resistance, and the systemic effect of drug treatments [297,298]. However, these approaches cannot exploit the detailed features offered by modern image-based kinematics as they only deal with 2D integrated or averaged metrics. Furthermore, despite all assumptions and simplifications, inverse approaches to lumped and 1D models are still prone to solution multiplicity [119]. With our focus on inverse modeling based on image-based kinematics, these approaches employing 1D simplified models fall outside the scope of this review.

To deal with the computational expense of the forward problem on inverse hemodynamics, Lassila et al. proposed a method for parametrizing the Navier–Stokes equations and patient-specific geometries to reduce the basis of the partial differential equations. The parameterized model is iteratively solved until the algorithm is close to the final solution. At this point, the inverse method then switches to the solution of the full-forward problem using FVM. This method was tested using deterministic and Bayesian optimization algorithms showing promising results on the solution of test cases involving rigid-wall and FSI simulations [103]. Herein, we review some of the existing research on inverse hemodynamics separating approaches that assume rigid-wall flow boundaries from those using FSI approaches.

#### 6.5.1. Rigid Wall Models

Romarowski et al. applied an iterative inverse method for the hemodynamic study of three descending thoracic aortic aneurysms. CT scans were used to build the 3D models that included the ascending and descending aorta [118]. PC MRI sequences were collected at the ascending aorta (above the aortic bulb), the suprarenal abdominal aorta, and all three branches of the aortic arch. Diastolic and systolic pressures were collected from sphygmomanometry. The authors observed that balances with the inlet and outlet flow rates measured with PC MRI did not comply with the conservation of mass principle. The forward problem was defined by applying the PC MRI-derived velocity distribution in the ascending aorta as an inlet boundary condition. At all four outlets, a surrogate three element Windkessel model of unknown parameters was imposed as a boundary condition, while blood was assumed to be an incompressible Newtonian fluid. The forward problem was solved by a FEM solver. An optimization algorithm was applied to minimize the least-squared error of the measured blood flow at the outlets to simulation estimates, by fitting the surrogate model parameters. The authors highlight that this weak approach allows distributing the error related to measurement noise while enforcing mass conservation. Similarly, Gaidzik et al. used PC MRI data from a healthy volunteer to find the pressure gradient distribution in the circle of Willis, an important cerebral arterial system [299]. In this work, Kalman filters are iterated over pressure boundary conditions to adjust the simulated flows to PC MRI measurements with an FVM solver for the forward problem. Noise-to-signal ratios were used to incorporate the measurement uncertainty into the data analysis. The authors highlight that the outputs of the inverse methods yield smaller uncertainties than CFD or 4D flow MRI data analysis alone.

Rispoli et al. proposed a modification to the implementation of FVM for fluid dynamics problems, to introduce the minimization of simulated nodal velocity components to 4D flow data measurements in the linearized SIMPLER algorithm [300]. The method required the smoothing and interpolation of coarse 4D flow MRI data to the FVM mesh. 4D flow MRI-derived velocities were directly used as inlet and outlet boundary conditions. The minimization problem and FVM solution were solved simultaneously using a version of the iterative Runge–Kutta algorithm. This method allowed the simultaneous solution of the simulation and inverse problems, thus reducing the computational expense. As a proof of concept, the method was applied to anatomy and 4D flow MRI scans of a healthy human carotid artery. The method was incorporated into a custom-made solver that required special discretization into a structured mesh in the Cartesian space. Töger et al. further developed this approach by incorporating the nodal velocity error minimization approach into a discontinuous Galerkin FEM formulation, allowing the solution of unstructured meshes [122]. The method was validated to in vitro measurements with laser particle imaging velocimetry in a pulsating flow loop with an abrupt change of cross-sectional area to induce complex flow patterns. Then, the method was applied to a healthy-human proximal cerebral artery. CT angiography was used to build the 3D anatomic model, 4D flow data were collected at a resolution of 0.7 mm voxel size with a 7 T scan, and PC MRI scans were collected at inlet and outlet planes with a resolution of 0.5 mm/px. Moreover, 4D flow data were spatially and temporally smoothed and interpolated into the FEM mesh, while PC MRI data were integrated to enforce inlet and outlet transient plug flow as boundary conditions. The method showed errors below 1% on velocity distribution for in vitro validation tests, and the proof of concept on in vivo datasets demonstrated the potential of the proposed methodology for future human studies.

#### 6.5.2. Fluid-Structure Interaction (FSI) Models

Fluid–structure interaction simulation is itself a complex, resource-consuming process, and its incorporation with inverse models is challenging. Some of the early work by Moireau, Chapelle, D’Elia, Perego, among others, set the bases for inverse modeling of FSI by calibrating models to in vitro experiments and synthetic datasets [301–303].

In 2014, Bertoglio et al. proposed the use of Kalman Filters to estimate the material properties of several regions of the aorta from inverse FSI [304]. Available clinical data included SSFP MRI, intravascular pressure measurements at the ascending, thoracic, and abdominal aorta, and PC MRI measurements at four planes along the aorta. The aorta was divided into four sections each one assumed to follow the Mooney–Rivlin material model. The arbitrary Lagrangian–Eulerian algorithm was implemented to couple fluid and wall mechanics. Kalman filter optimization was used to minimize an error function based on all available clinical measurements weighted by the associated uncertainty while fitting the regional material parameters. Results reproduced the expected stiffness distribution, with stiffer distal descending aorta.

Zambrano et al. proposed an iterative inverse method for the study of the pulmonary artery [305]. Intravascular pressure measurements, PC MRI at the main branches of the pulmonary artery, and cine MRI were collected from a pulmonary hypertensive adult patient and a healthy volunteer with no reported cardiovascular disease. A 3D model from the main pulmonary artery (MPA) down to the 4th branch generation was built from MRI images at the end-diastole configuration, which was considered stress-free. MRI-derived diameter changes were calculated at the main pulmonary artery and coupled to pressure measurements.

**Figure 15.**
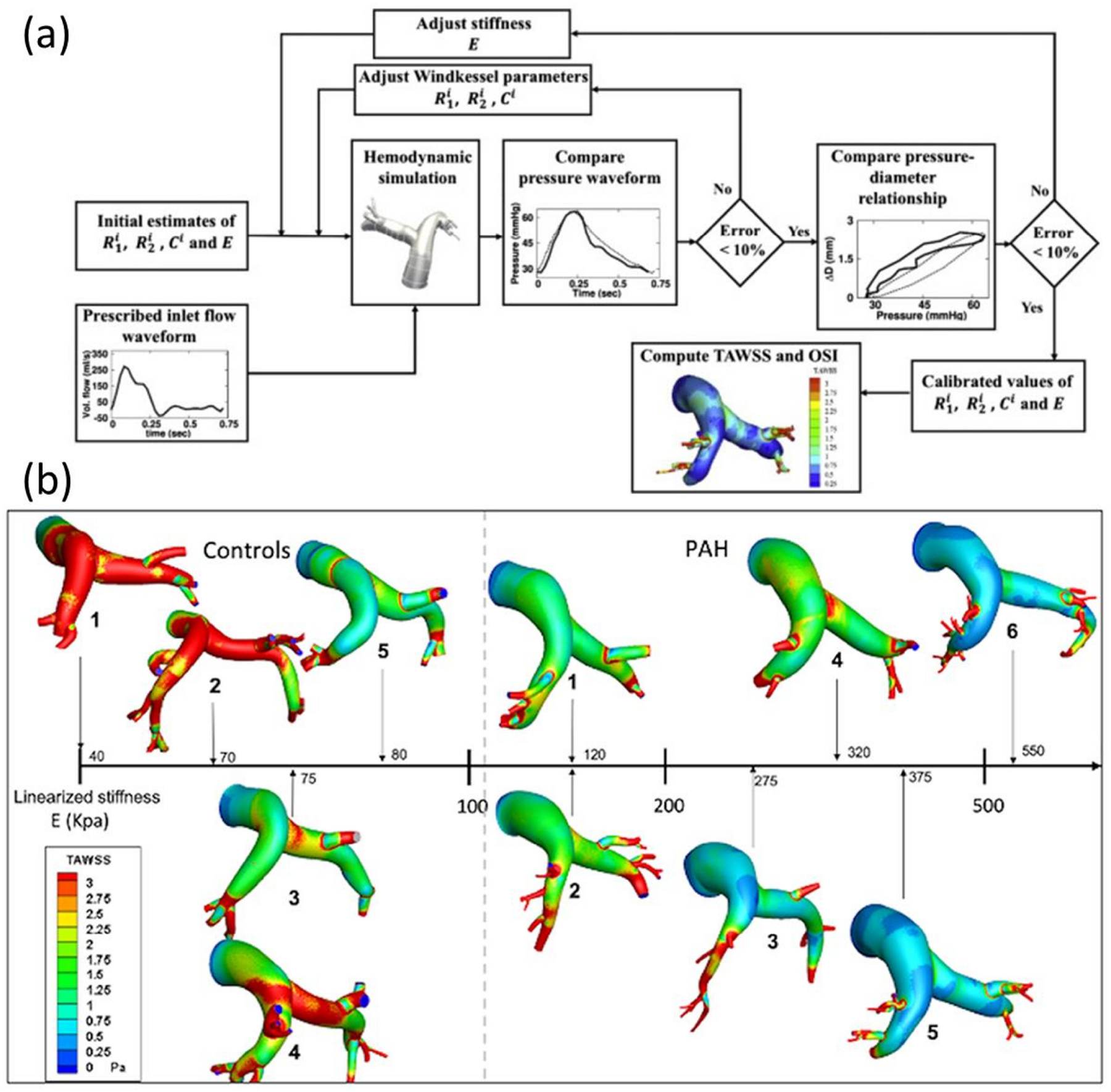
Iterative inverse method and results for the study of pulmonary artery hypertension. (**a**) Double optimization loop for the inverse resolution of distal vasculature Winkesel model parameters and wall elastic modulus from phase contrast MRI data. (Reprinted/adapted with permission from Ref. [305]. 2018, Elsevier) (**b**) Biomechanical parameters of pulmonary artery from healthy individuals and pulmonary artery hypertension patients. Models are organized from left to right according to the wall elastic modulus (stiffness) scale, colormaps show the time-averaged wall shear stress (TAWSS) distribution (Reprinted/adapted with permission from Ref. [306]. 2021, Zambrano et al.; open access).

The arterial wall was assumed homogeneous and isotropic linear elastic throughout the entire domain. The fluid–structure interactions were modeled with the coupled momentum method. Boundary conditions consisted of PC MRI-derived inlet flow and three-parameter Windkessel models in the outlets. The elastic modulus of the wall and Windkessel boundary parameters were calibrated by iterating in two nested loops. In the inner loop, the Windkessel parameters were adjusted until the error to the measured pressure waveform is minimized, while the outer loop adjusted the elastic modulus until the error to the measured pressure-area curve is minimized (Figure 15a). On each iteration, the forward problem was solved until solution periodicity was confirmed. The proposed model was able to reproduce the expected increase in arterial stiffness and vascular flow resistance in the hypertensive patient. In a follow-up study, the methodology was applied to a cohort of six individuals with pulmonary artery hypertension and five healthy volunteers [306]. A statistical analysis of the results revealed that the hypertensive group showed significantly larger wall stiffness, regurgitant flow, and distal vascular resistance, with significantly smaller time-averaged wall shear stress (Figure 15b). Interestingly, a linear correlation between the estimated wall elastic modulus and the magnitude of retrograde flow volume was found, which further supports the hypothesized relation between irregular flow patterns and the pathological remodeling of vascular tissue.

### 6.6. Summary

In Table 9 we summarize the highlights of inverse analyses for cardiovascular mechanics applications and notable results.

## 7. Closing Remarks

Inverse modeling is an analysis tool that can provide detailed information about domain properties and loading conditions using kinematic measurements as inputs. When applied to collected data from controlled in vitro experiments it can provide dynamic information with high levels of accuracy and reliability. In biomedical research, inverse modeling has been coupled with microscope-based imaging techniques to yield relevant information on the response of cardiovascular and engineered tissue to mechanical stimuli at the cellular level. These contributions hold relevant scientific value in the fields of mechanobiology and tissue engineering, however, the extrapolation of these results to patient-specific cases is limited.

There is great interest in the development of reliable patient-specific non-invasive medical tools to assess the onset and progression of cardiovascular disease. This has led to significant advances in non-invasive medical imaging, including improvements in resolution, scan time, operational costs, availability, and the ability to quantify detailed regional kinematic information. Inverse biomechanical analyses can exploit this available clinical data to provide patient-specific estimations of dynamic parameters that otherwise require invasive (and potentially risky) procedures, such as vascular catheterization, or cannot be measured at all. Inverse modeling fits dynamical unknowns to kinematic data, which would be simply assumed with fixed values on classical forward modeling approaches. However, inverse modeling cannot entirely substitute measurements of absolute pressure (required to define the loading boundary conditions); instead, this technique can be used to estimate other relevant biomarkers defined in terms of pressure or load differences, such as vascular flow resistance. As highlighted in this review of the clinical applications of these methodologies, inverse analyses can estimate stiffness for healthy and diseased cardiac and vascular tissues, identify and delineate pathological lesions, resolve tissue composition, and quantify mechanical loads and stresses during in vivo function. Inverse modeling can also provide physiological rationales for empirically derived risk factors, such as aneurysmal diameter and ventricular volume, as well as yield new sets of physiologically meaningful risk markers. In addition, inverse modeling can deliver insights into how biological tissues respond and adapt to pathology and/or therapies through comparative studies, such as regional changes in active contraction within infarcted hearts or tissue growth and remodeling in aneurysmal arteries.

Despite all these advantages, the incorporation of patient-specific inverse-modeling in clinical practice still faces several challenges, including the presence of multiple solutions, uncertainty regarding patient-specific stress-free reference configurations, computational costs, and the lack of required clinical and imaging data. The multiplicity of solutions is a common challenge to any inverse problem, and the solution set can be reduced by constraining the optimization parameters within ranges of expected values, incorporating regularization functionals, sampling stochastic parameters, designing special optimization target functions, and, for the specific case of Bayesian approaches, providing probability distributions of parameters from previous experiences.

A step towards resolving patient-specific stress-free references for tissue mechanics is the inverse solution of unloaded configurations through direct and iterative methods. However, it is generally accepted that unloaded blood vessels are not truly stress-free due to the existence of residual stress/strains which are influenced by the heterogeneous growth and continuous remodeling of the tissue, including the prestretch of key extracellular matrix components such as collagen. This issue could potentially be addressed by the implementation of a constrained mixture theory and the in vivo resolution of tissue microstructure via medical imaging.

The computational cost of iterative inverse methods is often addressed by simplifications of the forward problem, the use of surrogate models for early optimization stages, utilization of more efficient iterative optimization methods, and the use of parallel computing. Furthermore, the ongoing increase of computational power may allow the solution of complex problems that escapes the reach of current technology.

Similarly, it is reasonable to expect that medical imaging technology will continue to evolve, making them more readily available in healthcare practice. The development of data-driven techniques for the support of clinical decision making and treatment planning could also motivate the implementation of image-based kinematics in routine health care.

Inverse modeling is just one of many patient-specific techniques that have been proposed as a useful support for clinical practice. Machine learning has been increasingly explored in the last two decades for incorporation into the new field of precision medicine [307]. This technique consists of training decision-making algorithms with annotated large datasets, which when combined with the application of statistical principles, can return valuable evidence-based information from raw clinical data [308]. The main advantage of machine learning techniques is that once the algorithm has been trained, results can be obtained in short times with low associated computational cost. However, the outcomes are highly dependent on the quality of the annotated dataset used for training, as they are not the result of a physiology-based simulation but on statistical probabilities calculated from collected evidence. Thus, this approach can potentially fail if unique or unexpected conditions are presented.

An additional advantage of simulation-based techniques is their predictive capabilities. Founded on physical and physiological principles, patient-specific inverse problems can be coupled to mechanobiology-inspired growth and remodeling models to potentially predict the progression of diseases and/or the effect of treatments [106]. In conclusion, image-based inverse modeling is a promising quantitative tool to generate and analyze clinically relevant physiological data through a non-invasive approach with the ultimate goal of providing improved patient-specific diagnostic and prognostic assessments of diverse cardiovascular diseases in order to improve outcomes, reduce costs, and increase the quality of life.

## Figures and Tables

**Table 1.** Summary of governing principles of biomechanics.

Section	Highlights
2.	Inverse modeling of the cardiovascular system is usually grounded on classical continuum mechanics theory. The fundamental principles of mass and energy conservation are complemented by constitutive equations that describe the mechanical behavior of the material of interest. Constitutive material models can be either based on empirical evidence (phenomenological) or analytical expressions inspired by theory Once the model is defined through the selection of governing principles and constitutive equations, the problem is particularized by setting the domain of analysis and adequate boundary conditions.
2.1	Cardiovascular tissue is a complex multilayered structure that displays non-linear viscoelastic behavior, residual stress, and active contraction and distention. Structural mechanics of tissue is usually done with a Lagrangian formulation. The theory of finite hyperelasticity is applied to address the non-linear behavior and relatively large deformations. The adequate modeling of the passive behavior of cardiovascular tissue requires accounting for its structural anisotropy and the typical stiffening effect of strain/stretch. Active contraction and distention are the consequence of ion-based chemical signaling that triggers the contraction of actin-myosin sliding filaments, which determines the muscular tone. Active behavior is modeled by either adding an active stress or active strain components to the momentum balance. The additional active stress/strain is assumed to occur along myofiber directions and to depend on the cellular activation status. The geometrical distribution of the activation status can be determined by solving a reaction-diffusion problem. The patient-specific orientation of myofibers can be assessed by diffusion tensor MRI. However, the most common approach is to assume myofibers follow a standard orientation for which several models are available.
2.2	Blood is a suspension of cells in an aqueous solution of proteins and minerals that undergoes a pulsatile flow in vivo. Blood flow mechanics is typically studied with an Eulerian formulation. Assuming Newtonian fluid behavior and laminar flow are reasonable and typical approximations to model the blood flow in large vessels. Transition to turbulence flows may be relevant in the study of stenotic arteries. Phenomenological constitutive equations are available to model the shear-thinning effect on apparent viscosity.
2.3	The function of the cardiovascular system is the result of complex interactions between blood, the actively contractile cardiac tissue, and the compliant vascular walls. The interaction of blood flow and cardiovascular tissue requires specialized numerical formulations. There are several available formulations with different levels of complexity, one of which is the arbitrary Lagrangian–Eulerian algorithm which is complex and computationally expensive.
2.4	Living tissue has the capability to adapt in response to chemical and mechanical stimuli. The constrained mixture theory has been proposed to model the growth and remodeling of living tissue by solving sets of balance equations for each constituent of the tissue under study. The balance equations must be adequately constrained to account for the component-to-component interactions. The constrained mixture theory can introduce models to account for the reconfiguration of constituents under chemical/mechanical stimuli (remodeling).

**Table 2.** Summary of numerical methods.

Section	Highlights
3.	Mechanical analyses of complex biological systems require the application of numerical methods to obtain approximate solutions. There are several numerical methods available to solve the governing principles of continuum mechanics, including popular mesh-based methods such as the finite volume method (FVM) and finite element method (FEM). Mesh-based methods discretize the domain of study on spaces of finite size and iteratively solve the governing equations on each finite space simultaneously. FVM is based on a “strong” formulation that solves exactly the balance equations on the center of each finite volume. FEM is based on a “weak” formulation that assumes the unknown variable to follow a prescribed shape function within each finite element. The method converges to the solution by minimizing the weighted error induced by the discretization and use of the shape functions. FVM and FEM offer equivalent solutions to a variety of Multiphysics problems.

**Table 3.** Summary of inverse methods.

Section	Highlights
4.	Solving an inverse problem consists of using measured effects to estimate the causes. The main difficulties with inverse problems are the possible nonlinearity of the inverse mapping function, the multiplicity of solutions, and the sparsity and noise of the measured effect data.
4.1	The direct solution of inverse problems by the deduction of the inverse mapping function is only possible for simple cases. There are specialized mathematical solutions for specific problems of finite elasticity. Some relevant problems of inverse elasticity that have direct inverse solutions are (1) the solution of material properties from boundary loads and domain displacements. (2) The solution of the unloaded configuration from the applied loads, material properties and deformed configuration. Direct solutions of inverse problems are computationally efficient. However, direct solutions are not generalizable and require continuous smooth functions of the measured input often not compatible with noise and scarce experimental data.
4.2	Inverse problems can also be solved through an iterative weak approach. This consist of iteratively solving a forward simulation problem to minimize an error function between simulation outputs and target measurements while fitting the sets of unknowns. The iterative solution methods of inverse problems are generalizable, can handle noisy and scarce experimental target data, and can operate on top of existing simulation software. However, iterative methods are computationally expensive. The selection of the target function to be minimize needs to be consistent with the nature of the problem and the characteristics of the biomechanical model. The inverse method can be implemented though a variety of optimization methods. For the solutions of biomechanical inverse problems, optimization methods with no differentiation of the target function are preferred. Population-based optimization algorithms can solve global minima of multiparametric functions with an increased toll of computational expense. Statistic-based optimization method can incorporate previously reported data which can reduce convergence time and provide probability distributions of results rather than single deterministic values. Convergence times can be improved and solution multiplicity narrowed by the implementation of solution constraints. Constraints can be based on physical laws and limits or on previous experience. Constraints can also be implemented to promote numerical stability and smoothness of the converged solution.

**Table 4.** Image-based technique for assessment of tissue motion.

Technology	Technique	Principle	Resolution	Applications
US	Speckle Tracking	Acoustic response to the interaction of ultrasound signals with tissue fibers.	Spatial and displacement resolution < 1 mm/pixel Real-time temporal resolution.	Identification of: septal defects, CHD, valve structure. Assessment of cardiac and aortic function.
MRI	Tissue tagging	Local perturbation of myocardium magnetization with selective radiofrequency saturation sequences	Spatial and displacement resolution ~1 mm Tag spacing ~4 mm 25 images per cardiac cycle.	Assessment of cardiac function; motion and deformation of myocardium, skeletal muscle, lung tissue and tongue.
	DENSE MRI	Applied magnetic field gradients produce a phase shift on proton spins proportional to its relative displacement.	Pixel size ~2.5 mm for myocardial motion [149], ~1.3 mm for aortic motion [150] Displacement resolution < 0.1 mm. 30 images per cardiac cycle	Assessment of myocardial and aortic motion, deformation, and function.

**Table 5.** Image-based technique for assessment of blood flow.

Technology	Technique	Principle	Resolution	Applications
US	Echo and Vector Doppler	Measurement of frequency shift of the reflected acoustic wave.	Spatial resolution <1 mm/pixel	Identification of: septal defects, CHD, valve structure. Assessment of cardiac and arterial function. Prenatal care.
MRI	2D PC	Applied magnetic field gradients produce a phase shift on proton spins proportional to its relative velocity.	Pixel size ~1.5 mm 30 images per cardiac cycle	Assessment of cardiac, arterial, and venous flow, cardiac output, regurgitant flow, pulse wave velocity.
	4D flow		Pixel size ~2.5 mm 25 images per cardiac cycle	Same as 2D PC plus measurements of wall shear stress, vorticity and pressure drop.

**Table 6.** Summary of medical imaging-based kinematics.

Section	Highlights
5.	Early assessments of in vivo stiffness of blood vessels relied on measurements of luminal area changes. However, the predictive capabilities of these factors are inconsistent among different arterial locations and pathologies. In vivo medical imaging has evolved to provide not only anatomical geometric information but also detailed kinematic measurements. The accuracy and availability of these techniques are limited by image resolution, signal-to-noise ratio (SNR), the occurrence of artifacts, and practical obstacles related to testing costs and health hazards.
5.1	Ultrasound (US) uses high-frequency (2 to 15 MHz) acoustic waves to create real-time in vivo images of tissues, organs, and blood pools using piezoelectric transducers with lateral resolution of 1 mm/pixel. US is relatively inexpensive, portable, and safe, so it has become a customary tool in many clinical applications. However, the accuracy and reproducibility of US-derived measurements are limited in comparison to MRI-based measurements. Blood flow velocity can be assessed with echo and vector doppler technology. Tissue displacement can be measured using speckle tracking technology, which consists of image tracking the acoustic response of tissue fibers to ultrasound signals.
5.2	Magnetic resonance imaging (MRI) offers superior quantitative utility compared to ultrasound as it can offer higher resolution and accuracy of measurements of anatomical features. MRI generally poses minimal hazard to patients unless they have implanted medical devices/objects or suffer from claustrophobia. However, the technique requires specialized equipment and trained staff, which limits availability compared to US. MRI-based techniques for assessment of tissue kinematics include tissue tagging and DENSE MRI. Tissue tagging is based on image tracking of magnetically induced markers, while DENSE MRI encodes the tissue displacement on the phase of the MR signal. Tissue tagging and DENSE MRI have been used to assess the kinematics of the myocardium. However, the superior resolution and accuracy of DENSE MRI allow the assessment of aortic kinematics. Phase-contrast (PC) MRI is a technique that allows the time-resolved quantification of blood flow velocity in or through a 2D plane by encoding the velocity in the phase of the MRI signal. PC MRI has been generalized to 3D spaces at the expense of decreased spatial and temporal resolution. The resulting technique is called 4D flow MRI. PC MRI and 4D flow MRI have been applied to the study of healthy and pathological hemodynamics of the heart and large arteries and are currently implemented in clinical practice for the assessment of aortic and pulmonary diseases. MRI can provide other complementary information relevant for inverse modeling analyses of the cardiovascular system. Diffusion-tensor MRI can resolve the orientation of tissue fibers based on the principle that the Brownian displacement of water molecules occurs preferentially in the direction of fibers. Gadolinium-enhanced (GE) MRI can be used to resolve the size and severity of cardiac lesions. Since healthy cell membranes are impermeable to gadolinium, this contrast agent occupies a larger volume in injured tissue where cell membrane integrity is compromised. Perfusion stress tests use contrast agents and MRI imaging to assess the severity of coronary artery insufficiency. This is performed by comparing the perfusion of contrast agents in the myocardium at rest and at a stress state (high heart rate).
5.3	Computerized tomography (CT) provides the best resolution among all the medical imaging techniques with pixel sizes around 0.5 mm. The high-resolution CT images can be used to assess cardiovascular kinematics through image tracking of anatomical features. However, this requires the introduction of assumptions of displacement modes. CT scans are based on X-ray technology with inherent ionizing radiation hazards.

**Table 7.** Literature review of iterative inverse models for the analysis of human heart tissue mechanics.

Study	Clinical Data		Forward Problem		Inverse Problem	
Rumindo et al., 2020 [247]	Population	12 H	Reference	End of diastole	Target function	Least squared error to Klotz P-V
	Pathology	None	Passive model	Hom. Guccione		
	Data	Cine MRI	Active model	1 eq. active stress	Opt. algorithm	Nelder Mead.
	Anatomy	LV with RBFO by Rijcken et al.	Boundary	ICP, TF epicardium Constrained base		
Zhang et al., 2020 [17]	Population	1H 5D	Reference	Early diastole	Target function	Volume change error and segment-wise strain error
	Pathology	FMR-CAD	Passive model	Het. Guccione		
	Data	Cine MRI, TT, Stress perfusion MRI, GE MRI, 4D US, Hand cuff pressure	Active model	2 eq. active stress		Non-specified
	Anatomy	BV in 17 AHA regions with RBFO by Bayer et al.	Boundary	ICP, TF epicardium, Constrained base	Opt. algorithm	
Balaban et al., 2018 [141]	Population	1D	Reference	End of diastole	Target function	Deformation gradient error.
	Pathology	LBBB and CI	Passive model	Het. Holzapfel-Ogden		
	Data	4D US, USST, GE MRI, ICP	Active model	None	Opt. algorithm	Sequential quadratic programming with a first-order Tikhonov functional constraint
	Anatomy	LV in 17 AHA regions with RBFO by Bayer et al.	Boundary	ICP, Constrained apex, EF at base.		
Wang et al., 2018 [248]	Population	5H19D	Reference	Diastasis	Target function	Least-squared error to P-V curve.
	Pathology	HFrEF, HFpEF	Passive model	Hom. Guccione		
	Data	Cine MRI, ICP	Active model	None	Opt. algorithm	Non-specified
	Anatomy	LV with RBFO by Nielsen et al.	Boundary	* IPC * Constrained base		
Finsberg et al., 2019 [249]	Population	6H12D	Reference	Unloaded	Target function	Coordinate error for passive properties. P-V curve and strain error for active properties.
	Pathology	PAH	Passive model	Hom. Holzapfel-Ogden		
	Data	Cine MRI, ICP	Active model	1 eq. active strain	Opt. algorithm	Sequential quadratic programming algorithm
	Anatomy	BV with RBFO by Bayer et al.	Boundary	ICP, EF at base, EF pericardium		
Palit et al., 2018 [108]	Population	5H	Reference	Early Diastole	Target function	Least squared error to Klotz P-V
	Pathology	None	Passive model	Hom. Holzapfel-Ogden		
	Data	Cine MRI	Active model	None	Opt. algorithm	Genetic Algorithm
	Anatomy	BV with RBFO by Bayer et al.	Boundary	* Assumed ICP * Constrained base		
Finsberg et al., 2018 [235]	Population	7H 7D	Reference	Unloaded	Target function	Coordinate error for passive properties. P-V curve and strain error for active properties.
	Pathology	LBBB	Passive model	Hom. Holzapfel-Ogden		
	Data	4D US, USST, ICP	Active model	1 eq. active stress, 1 eq. active strain	Opt. algorithm	Sequential quadratic programming algorithm
	Anatomy	LV with RBFO by Bayer et al.	Boundary	ICP, EF at base, EF pericardium		
Asner et al., 2015, 2017 [250,251]	Population	3H 3P	Reference	End of diastole	Target function	P-V curve and nodal displacement error
	Pathology	Dilated cardiomyopathy	Passive model	Hom. Holzapfel-Ogden		
	Data	Cine MRI, TT, PC MRI	Active model	1 eq. active stress	Opt. algorithm	Shamanskii-Newton Raphson algorithm
	Anatomy	LV with fiber orientation from canine histology	Boundary	Weak formulation for volume and displacement		
Nasopoulou et al., 2017 [252]	Population	1H 7D	Reference	Lower ventricular pressure	Target function	Energy balance error and displacement error
	Pathology	Arrythmia	Passive model	Hom. Guccione		
	Data	Cine MRI, ICP	Active model	None	Opt. algorithm	Non-specified
	Anatomy	LV with fiber orientation from canine histology	Boundary	ICP, displacement at apex and base, TF epicardium		
Gao et al., 2017 [253]	Population	27H 11D	Reference	End of diastole	Target function	Volume error and strain error.
	Pathology	Acute myocardial infraction	Passive model	Het. Holzapfel-Ogden		
	Data	Cine MRI, GE MRI, Hand-cuff pressure	Active model	2eq. active stress	Opt. algorithm	Gaussian processes and automatic relevance determination algorithm
	Anatomy	LV in 17 AHA regions with RBFO by Potse et al.	Boundary	ICP, TF epicardium		
Genet et al., 2014 [110]	Population	5H	Reference	Early diastole	Target function	Least-squared-error to normalized Klotz P-V curve
	Pathology	None	Passive model	Hom. Guccione		
	Data	Cine MRI, TT	Active model	Hom. 1eq. active stress	Opt. algorithm	Derivative-free quadratic approximation algorithm
	Anatomy	LV with fiber orientation from canine histology	Boundary	Volume change, TF epicardium, Constrained base.		
Marchesseau et al., 2013 [254]	Population	8H 3D	Reference	End of diastole	Target function	Volume change error
	Pathology	HFrEF	Passive model	Region heterogeneous Mooney-Rivlin		
	Data	Cine MRI, ICP, Electrophysiology	Active model	2 eq. active stress	Opt. algorithm	Kalman filter
	Anatomy	BV divided in 17 regions with RBFO by Bayer et al.	Boundary	ICP, TF epicardium, Constrained base		
Xi et al., 2013, 2011a, 2011b [47,255,256]	Population	1H 2D	Reference	Unloaded	Target function	Nodal coordinate error
	Pathology	HFrEF	Passive model	Hom. Guccione		
	Data	Cine MRI, TT, ICP	Active model	1 eq. active stress	Opt. algorithm	Parameter sweeping
	Anatomy	LV with fiber orientation from canine histology	Boundary	ICP, TF epicardium, Displacement at apex and base.		

Abbreviations and acronyms: Clinical data: AHA, American Heart Association; BV, biventricular; D, diseased; GE, gadolinium enriched; H, healthy; ICP, intracardiac pressure; LV, left ventricle; MRI, magnetic resonance imaging; TT, tissue tagging; RBFO, ruled-based fiber orientation; US, ultrasound; USST, ultrasound speckle tracking. Pathologies: CI, cardiac infraction; FMR CAD: functional mitral regurgitation associated to coronary artery disease; HFpEF, heart failure with preserved ejection fraction; HFrEF, heart failure with reduced ejection fraction; LBBB, left bundle branch block. Forward problem: EF, elastic foundation; eq., equation; Het., heterogeneous; Hom., homogeneous; ICP, intracardiac pressure; TF, traction free.

**Table 8.** Literature review of iterative inverse models for the analysis of human arterial wall mechanics.

Study	Clinical Data		Forward Problem		Inverse Problem	
Bracamonte et al., 2022, 2021, 2020 [14,150,280]	Population	27H	Reference	Diastole	Target function	Least-squared displacement error
	Pathology	None	Passive model	Hom. Fung orthotropic		
	Data	Cine and DENSE MRI	Active model	None	Opt. algorithm	Constrained Powell
	Anatomy	IAA, DTA, DAA	Boundary	LP, Het. EF at adventitia		
Pourmodheji et al., 2021 [5]	Population	2D	Reference	Diastole	Target function	P-V curve error
	Pathology	PAH and CHD	Passive model	Constrained 4-fiber family		
	Data	IVP, Cine MRI, PC MRI	Active model	None	Opt. algorithm	L-BFGS
	Anatomy	Pulmonary Artery	Boundary	LP, TF adventitia		
Giuseppe et al., 2021 [281] Farzaneh et al., 2019 [112]	Population	30D	Reference	Diastole	Target function	Systolic shape.
	Pathology	aATA, w BAV and TAV	Passive model	Het. Linear elastic		
	Data	CT scans	Active model	None	Opt. algorithm	Direct solution
	Anatomy	Thoracic aorta	Boundary	LP and shape change		
Disseldorp et al., 2019, 2016 [282,283]	Population	30H 65D, 40D	Reference	Unloaded	Target function	Displacement error
	Pathology	AAA	Passive model	Hom. Neo-Hookean		
	Data	4D US, ST, CT scan, Hand cuff pressure	Active model	None	Opt. algorithm	Nelder-Mead
	Anatomy	IAA	Boundary	LP, AP		
Maso Talou et al., 2018 [284]	Population	4D	Reference	Unloaded	Target function	
	Pathology	Atherosclerosis	Passive model	Het. Neo-Hookean		Displacement error
	Data	IVUS	Active model	None	Opt. algorithm	Kalman filter
	Anatomy	Carotid artery bifurcation	Boundary	LP, EF at adventitia		
Liu et al., 2018 [113]	Population	4D	Reference	Diastole	Target function	Systolic shape error
	Pathology	aATA	Passive model	Hom. Holzapfel-Ogden		
	Data	CT scans	Active model	None	Opt. algorithm	multi-resolution direct search method
	Anatomy	Ascending Aorta	Boundary	LP, AP		
Wittek et al., 2016 [125]	Population	5H1D	Reference	Axially unloaded	Target function	Displacement error
	Pathology	PAO	Passive model	Hom. Holzapfel-Ogden		
	Data	4D US, ST, Hand cuff pressure	Active model	None	Opt. algorithm	Nelder-Mead with stochastic Montecarlo sampling
	Anatomy	IAA	Boundary	LP, AP		
Wang et al., 2017 [285] Liu et al., 2012 [286]	Population	8D	Reference	Unloaded	Target function	Area change error
	Pathology	Atherosclerosis	Passive model	Mooney-Rivlin		
	Data	Cine MRI, MC MRI, Hand cuff pressure	Active model	None	Opt. algorithm	L-BFGS-B
	Anatomy	Carotid artery bifurcation	Boundary	LP, TF adventitia		
Krishnan et al., 2015 [225]	Population	4D	Reference	Unloaded	Target function	Least-squared strain error
	Pathology	aATA	Passive model	Hom. Holzapfel-Ogden		
	Data	CT scan, DENSE MRI	Active model	None	Opt. algorithm	Non-specified
	Anatomy	Ascending Aorta	Boundary	LP, TF adventitia		
Karatolios et al., 2013 [164]	Population	6H2D	Reference	Axially unloaded	Target function	Displacement error
	Pathology	AAA	Passive model	Hom. Holzapfel-Ogden		
	Data	4D US, ST, Hand cuff pressure	Active model	None	Opt. algorithm	Nelder-Mead
	Anatomy	Abdominal aorta.	Boundary	LP, AP		
Wittek et al., 2013 [115]	Population	5H	Reference	Axially unloaded	Target function	Displacement error
	Pathology	None	Passive model	Hom. Holzapfel-Ogden		
	Data	4D US, ST, Hand cuff pressure	Active model	None	Opt. algorithm	Nelder-Mead
	Anatomy	IAA	Boundary	LP, AP		
Franquet et al., 2013 [114]	Population	2H	Reference	Diastole	Target function	Systolic shape error
	Pathology	None	Passive model	Hom. Linear isotropic		
	Data	Cine MRI, AT pressure	Active model	None	Opt. algorithm	Levenberg–Marquardt
	Anatomy	CCA	Boundary	LP, EF at adventitia		
Masson et al., 2010 [287]	Population	2H	Reference	Cut-open stress-free	Target function	Pressure waveform error
	Pathology	None	Passive model	Hom. Holzapfel-Ogden		
	Data	2D US, AT pressure	Active model	1 eq. active stress Area change,	Opt. algorithm	Levenberg–Marquardt
	Anatomy	CCA (idealized)	Boundary	Non-linear EF at adventitia.		
Taviani et al., 2008 [288]	Population	3H	Reference	Diastole	Target function	Area change error
	Pathology	None	Passive model	Hom. Linear isotropic		
	Data	Cine MRI, AT pressure	Active model	None	Opt. algorithm	Non-specified
	Anatomy	CCA	Boundary	LP, TF adventitia		

Abbreviations and acronyms: Clinical data: AT, applanation tonometry; CCA, common carotid artery; CT, computerized tomography; D, diseased; DAA, distal aortic arch; DTA, descending thoracic artery; H, healthy; IVUS, intravascular ultrasound; MC MRI, Multi-contrast magnetic resonance imaging; PAO, peripheral arterial occlusion; US, ultrasound; USST, ultrasound speckle tracking. Pathologies: AAA, abdominal aortic aneurysm; aATA, ascending aorta thoracic aneurysm; BAV, bicuspid aortic valve; CHD, congenital heart defect; TAV, tricuspid aortic valve. Forward problem: AP, adventitial pressure; EF, elastic foundation; eq., equation; Het., heterogeneous; Hom., homogeneous; LP, luminal pressure; TF, traction-free.

**Table 9.** Summary of medical imaging-based kinematics.

Section	Highlights
6.	The development of patient-specific inverse analyses of cardiovascular mechanics has advanced considerably recently thanks to continuous technological improvements in imaging hardware and software, decreasing cost, increased imaging availability, improvements in image-based kinematics acquisition and postprocessing, simulation engineering, and significant increases in computational power.
6.1	Blood vessels, in particular those of the arterial tree, function under physiological pressure load at all times and are axially pre-stretched; thus, none of the patient-specific configurations resolved by in vivo imaging is truly a stress-free or zero-strain configuration. The unloaded configuration of cardiovascular tissue is not truly stress-free. The residual stress is hypothesized to be the product of heterogeneous growth and remodeling of tissue. For patient-specific analyses, the material properties and zero-stress configuration are unknown. Thus, the solution to this problem requires the specification of at least two deformed and loaded states as input data. Direct methods for the solution of inverse elastostatic problems to determine the unloaded configuration of the heart and arteries have been incorporated into FEM solvers for hyperelastic and fiber-family material models. Several iterative methods for the solution of the unloaded configurations have been proposed. All these methods have in common that a single point or a collection of points on the surface are fixed, while forward inflation problems from unloaded configuration iterations to the known loaded configurations are solved until a convergence criterion is satisfied. Unloaded configuration iterations are estimated either by shrinking the known loaded configuration or by taking “backward” inflation steps. An alternative iterative approach is to solve the strain and stress distribution that balances the applied loads acting on the image-derived anatomic configurations without the resolution of the unloaded geometry.
6.2	The inverse modeling of the heart as a whole is currently unfeasible due to the complexity of the system and computational limitations. An accurate understanding of myocardial mechanics is key for the diagnosis and treatment of diverse cardiac pathologies, and potentially, to predict and stratify the risk of heart failure after infarct. The assumption of material homogeneity is a common and convenient simplification for forward and inverse models. Homogeneous models may be deemed to be adequate for the study of healthy hearts, or when the aim of the analysis is not centered on the study of focalized lesions. Homogeneous models can quantify the stiffening effect of infarct lesions and predict the natural compensation of the active component of the heart to maintain cardiac function after infarction. Modeling of material heterogeneity of the heart can provide better fits to kinematic data, can resolve property changes, and identify the location and severity of myocardial lesions. This comes with an increment of model complexity and computational expense. A common approach is to approximate spatial variations of myocardial properties and microstructure with region-wise heterogeneities. AHA standard division of the left ventricle is often used to define region-wise heterogeneity. Heterogenous models of the myocardium can identify the material properties of the infarcted zone, the border zone, and the unaffected tissue. Heterogeneous models can accurately predict how impaired activation of the myocardium affects the cardiac function in patients with left bundle branch block (LBBB). Inverse analyses with heterogeneous models have been used to predict the effect of ischemia on cardiac function, and its recovery after revascularization treatment.
6.3	Heart valves and leaflets are thin structures with complex motion that are difficult to resolve through in vivo imaging techniques. Owing to this, most studies on these structures are carried out in vitro. Recent developments in US imaging of heart valves are the first steps toward the in vivo inverse modeling of these structures.
6.4	Changes in mechanical properties of arterial walls have been associated with the onset of multiple cardiovascular pathologies and remain an important predictor of cardiovascular morbidity and mortality in clinical practice. The image-based resolution of vascular tissue kinematics is technically challenging due to the relative thinness of vascular walls. Inverse analyses of healthy arteries have been used to assess the stiffening effect of aging and to explore the effect of perivascular interaction on aortic mechanics. Aneurysms are a potentially fatal condition that consist of the enlargement of blood vessels caused by the remodeling of its wall. Aneurysmal rupture risk increases with maximum diameter on average for the entire population, although diameter alone struggles to predict rupture for any given individual. Inverse modeling has been used to obtain heterogeneous maps of mechanical stress and strain in thoracic and abdominal aneurysms and to assess the effect of disease progression on tissue stiffening. Atherosclerosis is a chronic inflammatory disease that manifests as the hardening and occlusion of arteries due to the build-up of plaque on the lumen of the arterial wall. The in vivo evaluation of the mechanical properties of atherosclerotic plaques and their mechanical environment through inverse modeling could support the assessment of risk associated with plaque rupture.
6.5	Computational modeling of hemodynamics is more resource consuming than tissue mechanics. Statistical analyses have shown that outputs of the inverse methods yield smaller uncertainties than CFD or 4D flow MRI data analysis alone. Inverse modeling of the fluid–structure interaction of the blood flow in the pulmonary arteries has been used to identify relevant markers of pulmonary artery hypertension. Among these markers are wall stiffness, wall shear stress and oscillation, pulse wave velocity, and regurgitant flow.
